# Bruno 1/CELF regulates splicing and cytoskeleton dynamics to ensure correct sarcomere assembly in *Drosophila* flight muscles

**DOI:** 10.1371/journal.pbio.3002575

**Published:** 2024-04-29

**Authors:** Elena Nikonova, Jenna DeCata, Marc Canela, Christiane Barz, Alexandra Esser, Jessica Bouterwek, Akanksha Roy, Heidemarie Gensler, Martin Heß, Tobias Straub, Ignasi Forne, Maria L. Spletter

**Affiliations:** 1 Biomedical Center, Department of Physiological Chemistry, Ludwig-Maximilians-Universität München, München, Germany; 2 School of Science and Engineering, Division of Biological and Biomedical Systems, Kansas City, Missouri, United States of America; 3 Faculty of Biology, Universitat de Barcelona, Barcelona, Spain; 4 Muscle Dynamics Group, Max Planck Institute of Biochemistry, München, Germany; 5 Department of Systematic Zoology, Biocenter, Faculty of Biology, Ludwig-Maximilians-Universität München, München, Germany; 6 Biomedical Center, Bioinformatics Core Unit, Ludwig-Maximilians-Universität München, München, Germany; 7 Biomedical Center, Protein Analysis Unit, Ludwig-Maximilians-Universität München, München, Germany; King’s College London, UNITED KINGDOM

## Abstract

Muscles undergo developmental transitions in gene expression and alternative splicing that are necessary to refine sarcomere structure and contractility. CUG-BP and ETR-3-like (CELF) family RNA-binding proteins are important regulators of RNA processing during myogenesis that are misregulated in diseases such as Myotonic Dystrophy Type I (DM1). Here, we report a conserved function for Bruno 1 (Bru1, Arrest), a CELF1/2 family homolog in *Drosophila*, during early muscle myogenesis. Loss of Bru1 in flight muscles results in disorganization of the actin cytoskeleton leading to aberrant myofiber compaction and defects in pre-myofibril formation. Temporally restricted rescue and RNAi knockdown demonstrate that early cytoskeletal defects interfere with subsequent steps in sarcomere growth and maturation. Early defects are distinct from a later requirement for *bru1* to regulate sarcomere assembly dynamics during myofiber maturation. We identify an imbalance in growth in sarcomere length and width during later stages of development as the mechanism driving abnormal radial growth, myofibril fusion, and the formation of hollow myofibrils in *bru1* mutant muscle. Molecularly, we characterize a genome-wide transition from immature to mature sarcomere gene isoform expression in flight muscle development that is blocked in *bru1* mutants. We further demonstrate that temporally restricted Bru1 rescue can partially alleviate hypercontraction in late pupal and adult stages, but it cannot restore myofiber function or correct structural deficits. Our results reveal the conserved nature of CELF function in regulating cytoskeletal dynamics in muscle development and demonstrate that defective RNA processing due to misexpression of CELF proteins causes wide-reaching structural defects and progressive malfunction of affected muscles that cannot be rescued by late-stage gene replacement.

## Abstract 2

The CELF family of RNA binding proteins are important regulators of RNA processing during myogenesis. This study shows that the Drosophila homolog Bruno1 promotes a transition to mature spliceD isoforms, which is necessary for myofibrillogenesis and balanced growth in sarcomere length and width in flight muscles.

## Introduction

Alternative splicing plays a key role in shaping the diverse contractile and morphological characteristics of different striated muscle fiber types [1,2]. For example, the heart expresses short splice isoforms of Titin that contribute to the high passive resting stiffness of cardiomyocytes [3,4], while skeletal muscles with a lower passive resting stiffness express longer and more flexible Titin isoforms [5,6]. Fast and slow muscle fibers express different isoforms of Troponin I (TnI) and Troponin T (TnT), resulting in differences in Ca^2+^ sensitivity and contractile dynamics [7]. The fiber-type-specific expression patterns of hundreds of exons are established during development, with transitions to mature isoforms promoting acquisition of fiber-type characteristic contractile properties [8–10]. Although the functional differences between most splice isoforms are still unknown, misregulation of alternative splicing and isoform expression in muscle diseases such as dilated cardiomyopathies and myotonic dystrophies contributes to contractile dysfunction [11–13], highlighting the importance of RNA regulation to normal muscle function. Even different muscle fiber types in model organisms such as *Drosophila melanogaster* have distinct alternative splicing profiles [14,15], indicating that the regulation of alternative splicing and structural isoform expression plays a conserved role in fine-tuning muscle structure and contractile properties.

CUG-BP- and ETR-3-like factor (CELF) family RNA-binding proteins (also known as Bruno-like proteins) are important regulators of RNA processing. CELF proteins contain 3 highly conserved RNA recognition motif (RRMs) domains that are jointly involved in binding to GU-rich recognition elements in RNA [16,17]. They regulate diverse steps in RNA processing, from alternative splicing to mRNA trafficking, stability, decay, and translation [18–20]. In striated muscles, CELF proteins are involved in regulating developmental transitions in alternative splicing. CELF1 and CELF2 promote embryonic splicing patterns in vertebrate heart and skeletal muscle [21,22], for example, promoting inclusion of cardiac troponin T (cTNT) exon 5 in embryonic heart affecting calcium sensitivity and contractility in mouse and chicken [23]. CELF1/2 levels are down-regulated 10-fold as heart and skeletal muscle mature [21,22], and overexpression of CELF1 during mouse heart development affects nearly 30% of developmental-associated splicing changes, largely promoting reversion to the embryonic splicing pattern [24]. While CELF1/2 are down-regulated in muscle development, Muscleblind-like family proteins MBNL1 and MBNL2 are in contrast up-regulated and promote mature splicing and polyadenylation patterns [21,25,26]. CELF1/2 and MBNL1/2 antagonistically co-regulate the alternative splicing of hundreds of exons in developing muscle [24,27]. The physiological relevance of this regulatory interaction is illustrated by the severity of muscle phenotypes in myotonic dystrophy type I (DM1) patients, where sequestration of MBNL1 through binding to a repeat expansion in the *DMPK* gene results in PKC-mediated stabilization and increased expression of the CELF1 protein [28,29], and a corresponding reversion from mature to embryonic isoform expression patterns [21,25,28,30]. Thus, CELF proteins are a key component of the RNA regulatory network that defines muscle structure and contractile ability during myogenesis.

The conservation of CELF protein function in myogenesis across the animal kingdom provides an opportunity to explore foundational mechanisms of RNA regulation in muscle. In zebrafish, CELF proteins are expressed in the developing mesoderm and Celf1 regulates somite development, binds to untranslated regulatory elements (UREs) and can mediate splicing of a rat α-actinin mini-gene [31–33]. In *C*. *elegans*, ETR-1, a CELF1 homolog, promotes muscle development through the regulation of alternative splicing and alternative 3′ exons [34,35]. We and others have previously shown that Bruno 1 (Bru1, Arrest), a CELF1/2 family homolog in *Drosophila*, acts as a splicing factor during maturation of the indirect flight muscles (IFMs) and regulates growth in sarcomere length and myosin contractility [14,36]. Bruno1 is also known to regulate posterior localization and translation repression of *oskar* and *gurken* mRNAs in *Drosophila* embryos, helping to establish the anterior-posterior axis during embryogenesis [37–41]. In IFM, Bru1 expression is activated by the master regulator of the fibrillar muscle fate Spalt major (Salm), and hundreds of IFM-specific splice events in structural genes are lost after *bru1* RNAi knockdown [14,36]. Although the direct targets of Bru1 and detailed molecular mechanisms that contribute to the Bru1 phenotype are not known, loss of a Bru1-regulated, IFM-specific isoform of Stretchin-Myosin light chain kinase (Strn-Mlck) is sufficient to induce hypercontraction, short sarcomeres, and loss of myofibers [14,42]. Bru1 genetically interacts with RNA-binding protein Rbfox1 in IFM, resulting in complete loss of sarcomeric structure when both proteins are knocked-down and mirroring a regulatory interaction observed in mammals [27,43]. Although Bru1 levels peak early in IFM development and are down-regulated in adult flies [43], all reported phenotypes for Bru1 affect later steps in sarcomere maturation after 48 h after puparium formation (APF) [14,36,43,44]. The question therefore arises if Bru1 has a function during early stages of IFM formation, congruent with the role of CELF1/2 in fetal muscle in vertebrates, or if CELF function in *Drosophila* muscle is mechanistically distinct.

The *Drosophila* IFM are an established and disease-relevant model for exploring basic mechanisms of muscle development and sarcomere assembly. Sarcomere structure is conserved, and in both insects and vertebrates, sarcomeres are built of actin thin filaments anchored at the Z-disc, myosin thick filaments anchored at the M-line, and Titin connecting filaments that span the thin and thick filaments [45–47]. Myosin binding to actin and filament sliding provides contractile force, while Titin influences muscle stiffness, force generation, and sarcomere length [48–51]. Analysis of *Drosophila* models of human diseases, for example, myotonic dystrophy, X-linked centronuclear myopathy, nemaline myopathy, and Duchenne muscular dystrophy, have proven informative and offer relevant insight into disease pathology [13,52,53]. Work in *Drosophila* also provides insight into conserved developmental mechanisms of myogenesis, including myoblast fusion, tendon attachment, sarcomerogenesis, growth, and myofibril maturation [54–56]. The *Drosophila* IFM consist of 6 dorsal-longitudinal (DLM) and 7 dorsal-ventral myofibers (DVM) in each thoracic hemisphere [57,58]. IFM myoblasts proliferate associated with the notum of the wing-disc, and then migrate and fuse to form IFM myotubes [59,60]. IFM myotubes establish tendon connections around 16 to 20 h APF [61], and then compact and undergo myofibrillogenesis around 32 h APF [47,62,63]. Sarcomeres are added to myofibrils as myofibers grow dramatically in length to span the entire thorax by 48 h APF, and from 60 h to 90 h APF sarcomeres grow to their mature size of 3.2 μm in length and 1.2 μm in width [47,64,65]. After 48 h APF, myofibrils undergo a maturation process where a switch in gene expression facilitates establishment of asynchronous and stretch-activation properties of fibrillar IFM [42,66]. This detailed understanding of myogenesis in a conserved genetic model system is a powerful tool that can be applied to understand how RNA regulation impacts sarcomere assembly and maturation.

Here, we report that the early requirement for CELF protein function in myogenesis is conserved in *Drosophila* Bruno 1 (Bru1). We generated a novel CRISPR-mediated mutant in *bru1* that revealed early phenotypes in cytoskeletal organization. Temporally restricted rescue and *bru1* RNAi knockdown demonstrated how initial cytoskeletal defects are propagated and disrupt later steps in sarcomere growth and maturation. We further define a later requirement during myofiber maturation for *bru1* to regulate sarcomere assembly dynamics, where abnormal radial growth in *bru1* mutant muscle promotes myofibril fusion and the formation of hollow myofibrils. Our data moreover identify a previously uncharacterized genome-wide transition from immature to mature sarcomere gene isoform expression in IFM that is blocked in *bru1* mutants. Consistent with the pleiotropic nature of the CELF misregulation phenotype, temporally restricted expression of Bru1 cannot restore myofiber function or correct structural deficits, but it does partially alleviate adult-stage hypercontraction. Our results reveal a conserved role for CELF family proteins to fine-tune sarcomere structure and function, and identify multiple distinct developmental mechanisms that contribute to the *bru1* mutant phenotype in IFM.

## Results

**Fig 1:**
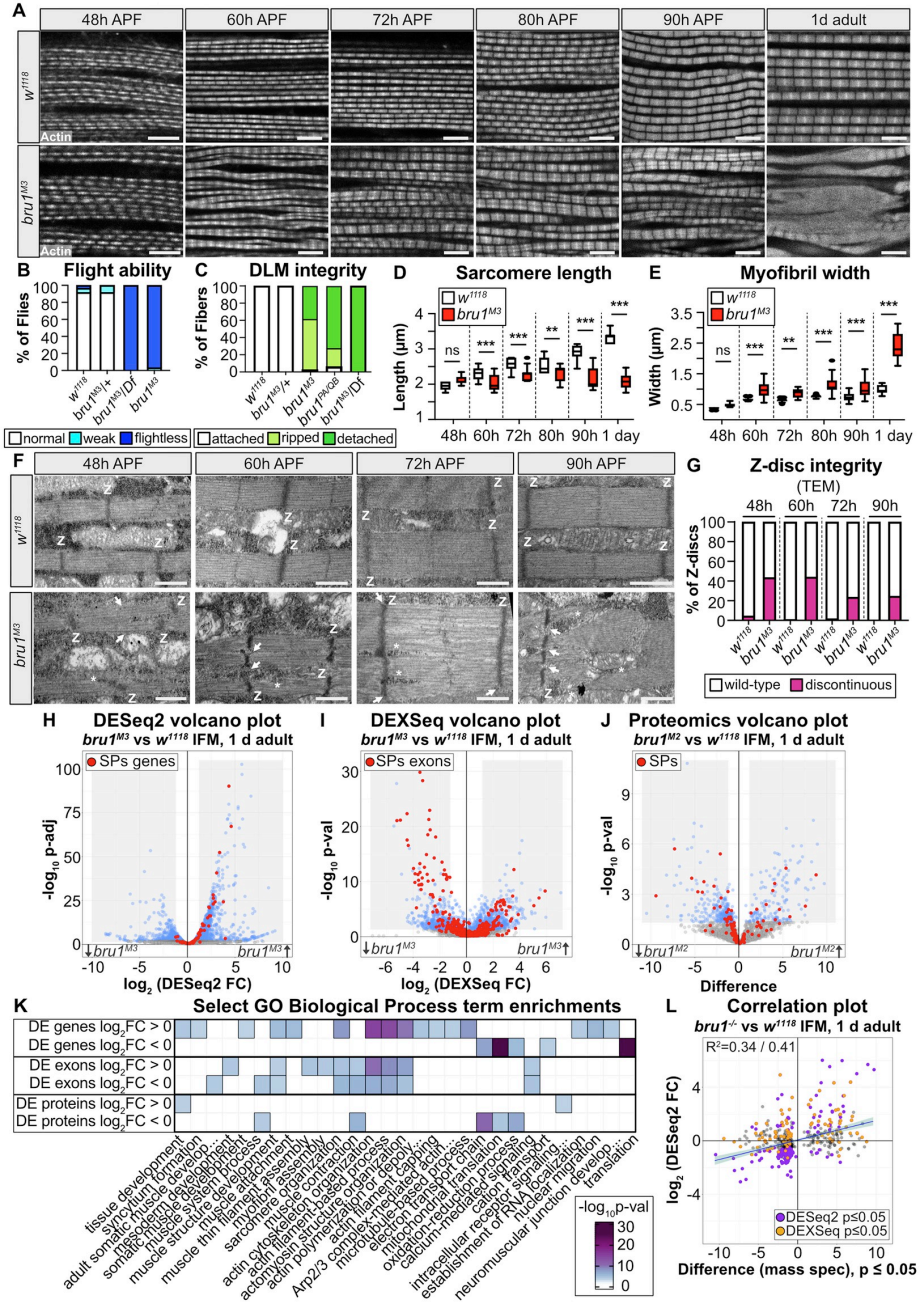
*bru1* mutant flight muscle displays misregulated sarcomere protein expression and progressively severe phenotypes during myofibril maturation. **(A)** Single-plane confocal images from thorax hemi-sections of *w*^*1118*^ and *bru1*^*M3*^ at 48 hour (h), 60 h, 72 h, 80 h, 90 h APF and 1 d adult flies. Phalloidin stained actin, gray; scale bar = 5 μm. **(B)** Quantification of flight ability. *N* > 30 flies for each genotype. **(C)** Quantification of myofiber phenotypes. *N* > 40 myofibers from 10 flies for each genotype. **(D, E)** Quantification of the sarcomere length (D) and myofibril width (E) from (A). Sarcomeres in control *w*^*1118*^ flies grow significantly in length from 2.0 ± 0.1 at 48 h APF to 2.9 ± 0.2 μm at 90 h APF (ANOVA, *p* < 0.001), while sarcomeres in *bru1*^*M3*^ do not (2.1 ± 0.1 to 2.2 ± 0.3 μm; ANOVA, *p* = 0.96). At 60 h APF, *bru1*^*M3*^ myofibrils are significantly wider than in wild type (0.99 ± 0.2 versus 0.66 ± 0.04 μm, ANOVA, *p* < 0.001). *bru1*^*M3*^ myofibrils significantly increase in width from 0.48 ± 0.06 at 48 h APF to 1.03 ± 0.26 μm at 90 h APF (ANOVA, *p* < 0.001). From 80 h APF to 1 d adult, *bru1*^*M3*^ sarcomeres shorten (2.3 ± 0.2 to 2.1 ± 0.2 μm; ANOVA, *p* = 0.009), while *w*^*1118*^ sarcomeres grow (2.5 ± 0.2 to 3.3 ± 0.1 μm; ANOVA, *p* < 0.001). Myofibril width increases more from 80 h APF to 1 d adult in *bru1*^*M3*^ (1.1 ± 0.3 μm to 2.4 ± 0.4 μm; ANOVA, *p* < 0.001) than in *w*^*1118*^ (0.76 ± 0.04 to 1.0 ± 0.1 μm; ANOVA, *p* = 0.002). Boxplots are shown with Tukey whiskers, outlier data points marked as black dots. Significance determined by ANOVA and post hoc Tukey (ns, not significant; **, *p* < 0.01; ***, *p* < 0.001). **(F)** TEM images of *w*^*1118*^ and *bru1*^*M3*^ sarcomere ultrastructure at 48 h, 60 h, 72 h, and 90 h APF. Defects in *bru1*^*M3*^ are already apparent at 48 h APF. Z-discs, “Z”; myofibril splitting and discontinuous Z-discs, white arrows; cytoplasm or mitochondrial inclusions, white asterisks; scale bar = 1 μm. **(G)** Quantification of Z-disc integrity in (F). *N* > 20 single planes for each individual genotype and time point. **(H, I)** mRNA-Seq volcano plots of DESeq2 gene expression (H) and DEXSeq exon use (I) changes in 1 d adult *bru1*^*M3-/-*^ versus *w*^*1118*^ IFM. SPs are notably affected (red dots). Gray boxes denote a threshold of abs(log_2_ fold-change) ≥ 1 and *p* ≤ 0.05, with significant events colored blue. **(J)** Volcano plot of peptide group expression (J) changes in *bru1*^*-/-*^ IFM from 1 d adults. Gray boxes denote a threshold of abs(Difference) ≥ 1 and *p* ≤ 0.05, significant peptides are colored blue. **(K)** Heatmap of select significantly enriched biological process GO terms in the DE genes, exons and proteins. **(L)** Dot plot of the correlation between significantly DE peptide groups and their corresponding mRNA expression level in *bru1*^*-/-*^ versus w^1118^ IFM. Proteins with a significantly DE exon (DEXSeq) are colored orange, and those significantly DE at the gene level (DESeq2) are colored purple. The Pearson’s/Spearman’s correlation coefficients (top left corner) and regression line (blue) indicate a weak but positive correlation. Underlying data can be found in S1 Table and Fig 1 Source Data files as listed in S6 Table. APF, after puparium formation; DE, differentially expressed; GO, gene ontology; IFM, indirect flight muscle; SP, sarcomere protein; TEM, transmission electron microscopy.

To investigate the function of Bru1 in *Drosophila* IFM development, we generated a new CRISPR allele that we refer to as *bru1*^*M3*^. *bru1*^*M3*^ is a truncation allele resulting from the integration of a splice-trap cassette upstream of *bru1* exon 18 (S1A and S1B Fig) that results in a near complete loss of detectable *bru1* mRNA and protein expression (S7A, S7C and S7C’ Fig). The splicing of *bru1* transcripts is redirected into the splice acceptor of the cassette instead of into exon 18, generating an early termination that effectively deletes the most C-terminal 88 amino acids in RRM3 of all *bru1* isoforms (S1C and S1D Fig). Based on phenotypes reported for the *aret*^*QB72*^ allele (EMS-induced stop at position 404) [14,38,67], as well as point mutations in RRM3 at positions 521 and 523 [39], *bru1*^*M3*^ is predicted to be a phenotypic null allele. Like other *bru1* alleles [38,67], *bru1*^*M3*^ is male and female sterile. Consistent with the reported adult IFM phenotype of *bru1* RNAi knockdown (*bru1-IR*) [14,36] and *bru1* alleles *bru1*^*M2*^ [43], *bru1*^*M1*^ [13], *bru1*^*QB72*^, *bru1*^*PD41*^, and *bru1*^*PA62*^ [14], we found that *bru1*^*M3*^ mutants are flightless and display a loss of myofibril and sarcomere architecture (Fig 1A–1C). We further confirmed the specificity of this phenotype over deficiency Df(2L)BSC407, which covers the *bru1* locus (Fig 1B and 1C, S1E, S1E’, S1F and S1F’ Fig). Together, these findings validate the nature and specificity of the *bru1*^*M3*^ allele and provide independent confirmation of a function for Bru1 in IFM development.

### Growth in sarcomere width and length is imbalanced in *bru1*^*M3*^ mutant IFM

We used the *bru1*^*M3*^ allele to perform a detailed analysis of IFM development from 48 h APF through 1 d adult in thorax hemi-section preparations. We reasoned that a phenotypic null mutant might produce a stronger phenotype than the *bru1-IR* knockdown used in previous studies [14,36]. *bru1*^*M3*^ mutant myofibrils are severely degraded in 1 d adult, but are structurally intact until 90 h APF. However, already at 60 h APF mutant myofibrils are split and irregular, and neighboring myofibrils appear partially aligned (Fig 1A). From 48 to 90 h APF, sarcomeres in control *w*^*1118*^ flies grow significantly in length, but sarcomeres in *bru1*^*M3*^ do not (Fig 1A and 1D), indicating that growth in sarcomere length in *bru1*^*M3*^ IFM is arrested after 48 h APF. By contrast, already at 60 h APF in *bru1*^*M3*^, myofibrils are significantly wider than in wild type, and myofibril width in *bru1*^*M3*^ IFM continues to increase significantly from 48 h to 90 h APF (Fig 1E). This suggests that although *bru1*^*M3*^ sarcomeres do not grow in length after 48 h APF, they do grow in width.

To better understand the myofibril growth defects in *bru1*^*M3*^ mutant IFM between 48 h to 90 h APF, we performed an ultrastructural time course analysis using transmission electron microscopy (TEM). Sarcomeres in *bru1*^*M3*^ IFM were significantly shorter and thicker than *w*^*1118*^ sarcomeres at 60 h, 72 h, and 90 h APF (Fig 1F, S1G and S1H Fig), confirming our observations from thorax hemi-sections (Fig 1A, 1D and 1E). Notably, already at 48 h APF, split myofibrils as well as discontinuous and misaligned z-discs were evident in *bru1*^*M3*^ IFM (Fig 1F and 1G, S1I and S1J Fig), indicating that myofibril defects in *bru1* mutants arise earlier than previously reported [14,36]. Myofibril splitting and z-disc defects were also detected in *bru1*^*M3*^ at the 60 h, 72 h, and 90 h time points (Fig 1F and 1G, S1I and S1J Fig). Peculiarly, starting from 60 h APF, we could identify cytoplasmic components and even mitochondria trapped in the middle of otherwise continuous myofibrils (Fig 1F), suggestive of an underlying defect in radial growth or myofibril fusion. This data thus reveals 2 novel aspects of the *bru1*^*M3*^ phenotype in IFM that we explore in more detail below: (1) during myofibril maturation in the absence of functional Bru1, there is an imbalance between growth in sarcomere length and width; and (2) defects in *bru1*^*M3*^ mutant myofibril structure are already evident at 48 h APF.

### Misregulation of gene expression and splicing lead to protein expression defects in *bru1*^*M3*^ muscle

We next investigated the molecular phenotype underlying the myofibril defects observed in *bru1*^*M3*^ IFM. We performed mRNA-Seq and whole proteome mass spectrometry on IFM dissected from 0- to 24-hour-old (1 d adult) wild-type *w*^*1118*^ or *bru1* mutant flies, to evaluate changes on both the RNA and protein levels (Fig 1H–1J and S1 Table). A differential expression analysis with DESeq2 revealed hundreds of significant changes in gene expression in the mRNA-Seq data (Fig 1H). Up-regulated genes were enriched for biological process gene ontology (GO) terms such as “muscle attachment,” “sarcomere organization,” “actin cytoskeletal organization,” “actin filament capping,” and “establishment of RNA localization” (Fig 1K). Down-regulated genes were in contrast enriched for terms such as “translation,” “cation transport,” and “oxidation-reduction process.” Using DEXSeq, we further detected hundreds of significant changes in exon use in *bru1*^*M3*^ versus *w*^*1118*^ IFM (Fig 1I), reflecting changes in alternative splicing as well as alternative promoter use. Notably, both up-regulated and down-regulated exons were enriched for GO terms such as “sarcomere organization,” “actin cytoskeleton organization,” “muscle contraction,” and “calcium-mediated signaling” (Fig 1K). This likely reflects isoform switches in structural genes, as for example sarcomere proteins (SPs) display both up- and down-regulated exons (Fig 1I), which we investigate in more detail below. Interestingly, on the gene level, SPs are mostly up-regulated in *bru1*^*M3*^ versus *w*^*1118*^ IFM, potentially reflecting transcriptional compensation in response to changes in isoform use (Fig 1H). We conclude that loss of Bru1 function leads to changes in both gene expression and alternative splicing.

We complimented our transcriptomic data with proteomics from 1 d adult IFM to evaluate if mRNA-level changes translate to altered protein expression. We grouped detected peptides into protein groups, such that peptides from the same gene that are unique to different protein isoforms form distinct protein groups. Analysis of the proteomics data revealed significant changes in the expression of hundreds of protein groups, with a bias toward down-regulation (Fig 1J). Down-regulated proteins were enriched in GO terms such as “muscle system process,” “muscle contraction,” “electron transport chain,” and “oxidation-reduction process,” while up-regulated proteins were enriched in “tissue development” and “intracellular receptor signaling process” (Fig 1K). As in the exon use analysis, we see up- and down-regulation of different sets of protein groups from SPs (Fig 1J). We then tested if there is a relationship between the changes in gene expression at the mRNA and protein level, and observed a weak but positive correlation for all significantly changed protein groups (Pearson’s R^2^ = 0.34, Spearman’s R^2^ = 0.41) (Fig 1L). Interestingly, we saw that both changes in gene expression as well as changes in exon use correlated with differential expression on the protein level (Fig 1L and S1K Fig). We also noted that different categories of genes show distinct patterns of regulation on the mRNA and protein level. Cytoskeletal genes such as SPs, genes involved in actin cytoskeleton organization, and microtubule-associated genes tend to be up-regulated at the gene level, but show both up- and down-regulation in the DEXSeq and proteomics data (S2A Fig). Mitochondrial and fibrillar core genes [14], by contrast, have up- and down-regulated exons, but are down-regulated on both the gene and protein level (S2A Fig). We conclude that Bru1-mediated changes to the IFM transcriptome are complex and indeed alter both protein and protein isoform expression.

### Protein-level misexpression reflects alternative splicing changes in *bru1*^*M3*^ IFM

**Fig 2:**
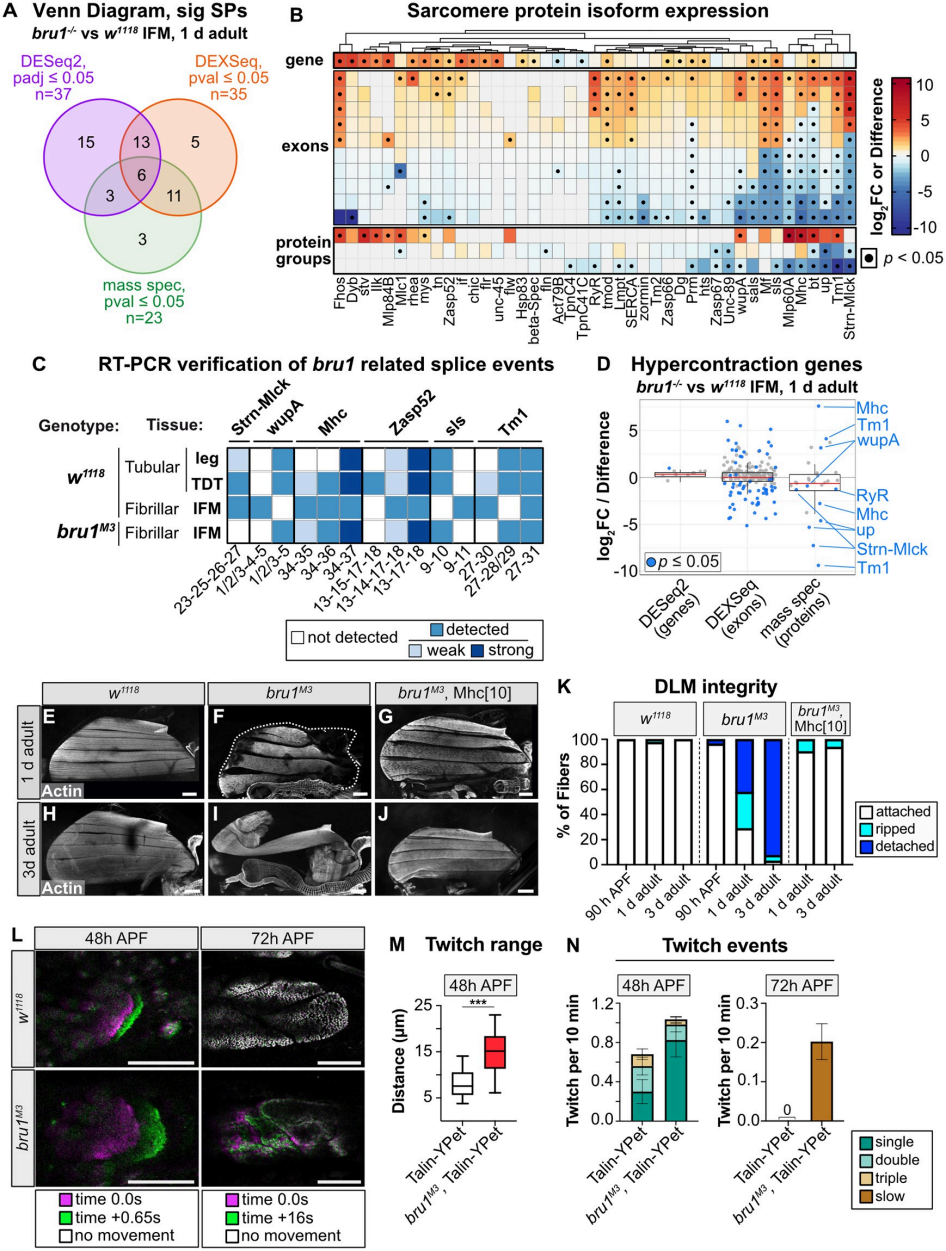
Misexpression of muscle-type-specific protein isoforms results in hypercontraction and abnormal contractile dynamics in *bru1*^*-/-*^ IFM. **(A)** Venn diagram of the overlap between SPs differentially expressed at the gene level (purple), the protein level (green) or that have differentially used exons (orange). **(B)** Hierarchical clustering and heat map of the coordinated changes in SP gene expression (top, DESeq2 log_2_FC), exon use (middle, top 10 DEXSeq log_2_FC values), and protein expression (bottom, Perseus Difference of the top 3 protein groups). Black dot denotes *p* ≤ 0.05. **(C)** Summary heatmap of semi-quantitative RT-PCR results (S2 Fig) verifying muscle-type specificity of select alternative splice events in *Strn-Mlck*, *wupA*, *Mhc*, *Zasp52*, *sls*, and *Tm1* in wild-type fly muscles, and loss or gain of those events in *bru1*^*M3*^ IFM (not detected by PCR, white; detected by PCR, blue; weak band, light blue; strong band, dark blue). **(D)** Boxplot of gene, exon, and protein-level expression changes in genes with associated hypercontraction phenotypes (S1 and S2 Tables). Significantly DE proteins in *bru1*^*M3*^ IFM are labeled; blue dot denotes *p* ≤ 0.05. **(E–J)** Confocal Z-stack images of DLM myofibers in w^*1118*^, *bru1*^*M3*^ and *bru1*^*M3*^, Mhc [10] from 1 d and 3 d adults. Myofiber loss and hypercontraction in *bru1*^*-/-*^ IFM is alleviated in the Mhc [10] background (G, J). Thorax boundaries in (F), dashed line; phalloidin stained actin, gray; scale bar = 100 μm. **(K)** Quantification of myofiber tearing and detachment phenotypes from (E–J) at 90 h APF, 1 d and 3 d adults. *N* > 40 myofibers from at least 10 flies for each individual genotype and time point. **(L)** Snapshots from live movies of Talin-YPet labeled DLMs at 48 h and 72 h APF from *w*^*1118*^ and *bru1*^*M3*^ animals. Time 0.0 (magenta) is overlaid with time +0.65 s (green; at 48 h APF) or +16 s (green; at 72 h APF). A complete overlap (white) depicts no movement. Scale bar = 50 μm. **(M)** Quantification of distance of maximum myofiber extension at 48 h APF in control and *bru1*^*M3*^. Boxplots are shown with Tukey whiskers, significance by unpaired *t* test (***, *p* < 0.001). **(N)** Quantification of spontaneous contraction events per fiber per 10 min in control and *bru1*^*M3*^. At 72 h APF, *bru1*^*M3*^ DLM fibers continue to undergo slow, unidirectional extension. *N* > 50 fibers/10 animals for each genotype and time point. Error bars = SEM. Underlying data can be found in S2 Table and Fig 2 Source Data files as listed in S6 Table. APF, after puparium formation; DE, differentially expressed; DLM, dorsal-longitudinal myofiber; IFM, indirect flight muscle; SP, sarcomere protein.

Reasoning that the strong changes in structural gene expression at both the mRNA and protein level might contribute to the myofibril defects in *bru1*^*M3*^ IFM, we analyzed sarcomere protein expression in greater detail. We noted that while 39% (9 of 23) of significantly differentially expressed (DE) sarcomere proteins were differentially regulated on the mRNA level (compared to 36% (184 of 514) of all significantly DE proteins), 74% (17 of 23) displayed significantly differential exon use (compared to 84 of 514 or 16% of all proteins) (Fig 2A and S2A Fig). We therefore compared changes in gene expression, exon use, and protein isoform expression in SPs (Fig 2B and S2 Table). We found a clear correspondence between changes in mRNA expression or splicing and altered protein expression level. For some SPs, for example Fhos, Ilk, or Mlp84B, gene expression changes match observed protein-level changes (Fig 2B). For other SPs, for example Mlp60A, wupA, Mhc, bt and Tm1, we see a clear switch in isoform expression, where changes in exon use match observed protein isoform changes (Fig 2B). This data also illustrates the breadth of sarcomere assembly processes impacted in *bru1* mutant muscle, as misexpressed SPs include previously identified regulators of thin and thick filament growth, structural components of the z-disc and M-line, components of the integrin and cell adhesion machinery, and regulators of actomyosin interactions [68].

To independently confirm changes in alternative splicing and protein isoform expression in *bru1*^*M3*^ IFM, we used GFP-tagged reporters and RT-PCR to test a panel of events in *Strn-Mlck*, *wupA*, *Mhc*, *Zasp52*, *sls*, and *Tm1* (Fig 2C). We confirmed the loss of expression of *Strn-Mlck* isoform R (exons 23 and 25) at both the mRNA and protein level in *bru1*^*M3*^ IFM (Fig 2C, S2C, S2F, S2G, S2G’, S2H and S2H’ Fig), which was previously shown to result in IFM hypercontraction [14]. In *wupA*, which encodes Troponin I [69], we confirmed an isoform switch from the IFM- to the tubular-specific termination (exon 4) at both the mRNA and protein levels (Fig 2C, S2D, S2I, S2J, S2J’, S2K and S2K’ Fig). We found that an alternative termination of *Mhc* that is used at early stages of IFM development and in tubular muscle (exon 37) [44,66] is expressed at increased levels in 1 d adult *bru1*^*M3*^ IFM at both mRNA and protein levels (Fig 2C, S2E, S2L, S2M, S2M’, S2N and S2N’ Fig). Interestingly, this Mhc isoform is incorporated uniformly across the width of the myofibril in *bru1*^*M3*^, instead of restricted to the center of the myofibrils as observed in control IFM (S2M, S2M’, S2N and S2N’ Fig). We further could confirm a switch in exon use in *Zasp52* exon 14/15, *sls* exon 10 and *Tm1* exon 28/29 and 30 (Fig 2C, S2O, S2P and S2Q Fig). This independent dataset validates our transcriptome and proteome analyses and confirms that Bru1 regulates alternative splicing and protein isoform expression of sarcomere proteins in IFM.

### *bru1*^*M3*^ mutant myofibers experience abnormal contractility throughout development

Several of the proteins we verified to have altered isoform expression in *bru1*^*M3*^ mutants share a function in regulating actomyosin interactions, suggesting that normal contractile dynamics may be altered after loss of Bru1. Specifically, when we curated a list of genes with reported hypercontraction phenotypes in fly muscle, we noticed significant changes in alternative splicing and protein isoform expression in Mhc, Tm1, wupA, RyR, Strn-Mlck, and up (TnT) (Fig 2D, S2H’, S2K’ and S2N’ Fig). Misregulated actomyosin interactions can lead to hypercontraction, a condition characterized by aberrant contractility and short, thick sarcomeres [70–72]. In our time course analysis, we noticed a dramatic increase in the severity of the *bru1*^*M3*^ phenotype at late stages of pupal development (Fig 1A, 1D and 1E). From 80 h APF to 1 d adult, *w*^*1118*^ sarcomeres grow in length while sarcomeres in *bru1*^*M3*^ shorten significantly and more than double in width (Fig 1D and 1E). Additionally, while IFM myofibers are attached at 90 h APF in *bru1*^*M3*^ mutants, at 1 day 71% and by 3 days 97% of myofibers are torn or completely detached (Fig 2E, 2F, 2H, 2I and 2K). A hypercontraction phenotype can be rescued by minimizing actomyosin forces, and we confirmed that *bru1*^*M3*^ myofibers remain attached in an *Mhc*^*10*^ mutant background in both 1- and 3-day adults (Fig 2G, 2J and 2K). This verifies that myofiber loss is indeed myosin-activity dependent, consistent with previous RNAi results [14,36]. We conclude that from 80 h APF, *bru1*^*M3*^ myofibrils experience hypercontraction as characterized by progressive shortening and thickening of sarcomeres and eventual myofiber loss in adult flies.

Actomyosin-dependent tension also plays an important role during early stages of IFM development to organize and refine sarcomere structure [73,74]. Spontaneous contractions, or twitches, are evident at 34 h APF shortly after sarcomere assembly, reach peak intensity around 48 h APF, and are suppressed by 72 h APF as IFM myofibrils develop stretch-activation properties [42]. To determine if actomyosin contractility is disrupted in *bru1*^*M3*^ mutants during IFM development, we performed live imaging of IFM labeled with Talin-YPet. At 48 h APF, we could detect twitch events in both *bru1*^*M3*^ and control *w*^*1118*^ myofibers (Fig 2L and 2N and S1 Movie). Strikingly, contractions in *bru1*^*M3*^ myofibers were stronger than in *w*^*1118*^ myofibers, resulting in a greater displacement of the myofiber tip (Fig 2M), and occurred more frequently (Fig 2N). At 72 h APF, we were unable to detect any movement in *w*^*1118*^ myofibers (Fig 2L and 2N). Unexpectedly, in 17 of 89 *bru1*^*M3*^ myofibers (19%), we observed a slow twitch movement (Fig 2L and 2N and S2 Movie). These fibers slowly contracted over a period of 16 s, as compared to less than 1 s at 48 h APF, and did not efficiently reextend. We conclude that actomyosin interactions are atypical throughout IFM development in *bru1* mutants, with abnormal contractility first contributing to sarcomere organization defects and then resulting in hypercontraction in late pupa and adult flies.

### Formation of hollow myofibrils is driven by misregulated radial growth and myofibril fusion

**Fig 3:**
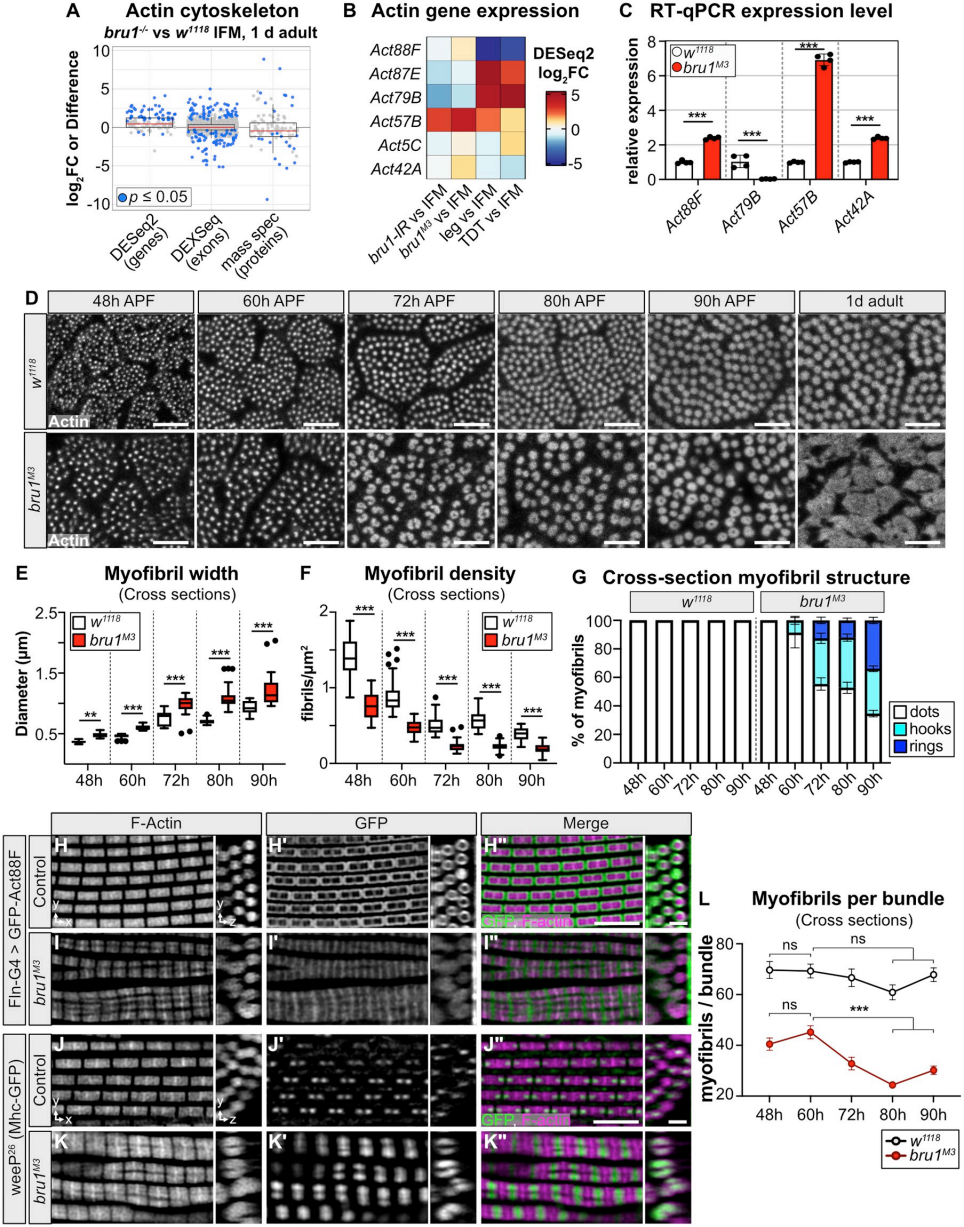
Hollow myofibril formation in *bru1* mutants is an active process resulting from defective expression and splicing of cytoskeletal genes and aberrant sarcomere growth. **(A)** Boxplot of gene, exon, and protein level expression changes between 1 d adult *bru1*^*-/-*^ and w^1118^ IFM in genes from GO term “actin cytoskeleton.” Blue dot denotes *p* ≤ 0.05. **(B)** Heatmap of *actin* gene expression in *bru1-IR*, *bru1*^*M3*^, leg and TDT as compared to wild-type IFM. **(C)** RT-qPCR verification of *Actin* gene expression levels between *bru1*^*M3*^ and wild-type *w*^*1118*^ IFM. **(D)** Confocal micrographs of DLM myofibril cross-sections in *w*^*1118*^ and *bru1*^*M3*^ at 48 h, 60 h, 72 h, 80 h, 90 h APF, and 1 d adult. Phalloidin stained actin, gray; scale bar = 5 μm. **(E, F)** Quantification of myofibril width (E) and density (F) in (D). *bru1*^*M3*^ mutant myofibrils are significantly wider than in wild type at 48 h APF (0.48 ± 0.04 μm versus 0.37 ± 0.02 μm, ANOVA *p* = 0.001), and there are fewer myofibrils (0.76 ± 0.02 μm versus 1.4 ± 0.2 myofibrils per μm^2^, ANOVA *p* < 0.001). Boxplots are shown with Tukey whiskers, outlier data points marked as black dots. Significance determined by ANOVA with post hoc Tukey (**, *p* < 0.01; ***, *p* < 0.001). **(G)** Quantification of myofibril structural morphology in (D). *bru1*^*M3*^ myofibrils progressively form hook and ring structures starting from 60 h APF. *N* > 10 animals per genotype and time point. Error bars = SEM. **(H–I”)** Deconvoluted confocal images at 90 h APF of *Fln*-Gal4 driven UASp-GFP-Actin88F incorporation into control (H-H”) and *bru1*^*M3*^ (I-I”) sarcomeres. Both xy- and zy-projections are shown. *Fln*-Gal4 expression from approximately 56 h APF results in a box-like pattern of GFP-Act88F incorporation (H’-H”) into growing wild-type sarcomeres, which is abnormal in *bru1*^*M3*^ (I’-I”). GFP, green; phalloidin stained actin, magenta; scale bar = 5 μm. **(J–K”)** Deconvoluted confocal images at 90 h APF of Mhc-weeP26-GFP incorporation into control (J-J”) and *bru1*^*M3*^ (K-K”) sarcomeres. Expression of GFP-labeled Mhc isoforms containing exon 37 is restricted to early developmental stages in IFM, resulting in a dot-like pattern flanking the M-line in wild-type sarcomeres (J’-J”) which is disrupted in *bru1*^*M3*^ (K’-K”). weeP26-GFP, green; phalloidin stained actin, magenta; scale bar = 5 μm. **(L)** Quantification of myofibril number per fiber bundle in *w*^*1118*^ and *bru1*^*M3*^ at 48 h, 60 h, 72 h, 80 h, and 90 h APF. There is a significant reduction from 60 h to 80 h APF in *bru1*^*M3*^ (45 ± 9 to 24 ± 5 myofibrils per bundle, ANOVA, *p* < 0.001) but not in *w*^*1118*^ IFM (69 ± 15 to 68 ± 14 myofibrils per bundle, ANOVA, *p* = 0.30). Plot represents the mean ± SEM. Significance determined by ANOVA and post hoc Tukey (ns, not significant; **, *p* < 0.01). *N* > 8 animals per genotype and time point. Underlying data can be found in Fig 3 Source Data and the RNA-Seq data tables as listed in S6 Table. APF, after puparium formation; DLM, dorsal-longitudinal myofiber; GO, gene ontology; IFM, indirect flight muscle.

Another subset of SPs misexpressed in *bru1*^*M3*^ IFM, including *Fhos*, *sals*, *sls*, *Mlp84B*, *Unc-89*, *tmod*, and *Zasp52*, are known regulators of sarcomere growth and integrity [68]. These genes caught our attention, as we observed splitting and an abnormal balance between growth in length and width in *bru1*^*M3*^ sarcomeres (Fig 1A and 1F). We also noticed that not just SPs, but more broadly actin cytoskeleton and microtubule-associated genes, are misregulated in *bru1*^*M3*^ (Fig 3A and S2A Fig). When we examined actin gene expression, we found altered expression ratios in our mRNA-Seq data that we could further confirm by RT-qPCR (Fig 3B and 3C, and S3A Fig), including a dramatic up-regulation of cardiac actin Act57B. Previous studies have shown that actin genes have differential abilities to integrate into the growing sarcomere and misexpression of cardiac Act57B disrupts IFM function [75,76]. We reasoned that the altered ratios in actin gene expression in *bru1*^*M3*^ IFM, together with changes in key cytoskeletal regulators, might lead to aberrant sarcomere growth.

To evaluate defects in lateral or radial sarcomere growth in greater detail, we performed a cross-section time course analysis of *bru1*^*M3*^ IFM development. In wild-type *w*^*1118*^ IFM, myofibrils are organized into distinct bundles and grow uniformly in the radial direction from 48 h to 1 d adult, maintaining a consistent, circular appearance in cross-section (Fig 3D). Myofibrils in *bru1*^*M3*^ flies are also organized into bundles, but already at 48 h APF, mutant myofibrils are significantly wider than in wild type (Fig 3D and 3E), and there are significantly fewer myofibrils (Fig 3F). Strikingly, although *bru1*^*M3*^ myofibrils appear uniform and circular at 48 h APF, by 60 h APF there is variability among the sizes of individual myofibrils and some appear more oval or hook-like than circular (Fig 3D and 3G). As the myofibrils develop from 60 h to 90 h APF, this hooked phenotype becomes more pronounced, affecting 8.7 ± 2.8% of myofibrils at 60 h APF and 31% to 35% of myofibrils at 72 h, 80 h, and 90 h APF (Fig 3G). Concurrently, we see the progressive development of rings, or hollow myofibrils, affecting 12.6 ± 2.2% of myofibrils at 72 h APF and 33.9 ± 2.2% of all myofibrils at 90 h APF (Fig 3D and 3G). In 1 d adults, *bru1*^*M3*^ myofibrils are highly irregular and display a strong atrophic phenotype. Taken together, this analysis reveals a distinct temporal progression in the appearance of myofibril defects, suggesting atypical and irregular radial growth during myofibril maturation. Moreover, such growth could provide a mechanism whereby cytoplasmic components or even organelles become trapped in the middle of myofibrils, as we observed in our TEM data (Fig 1F).

To test if sarcomere growth is indeed aberrant in *bru1*^*M3*^ IFM, we adapted an approach to visualize thin filament growth dynamics using temporally restricted expression of a GFP-tagged actin [65]. Flight muscles predominantly express Act88F, which is incorporated into thin filaments both laterally and radially as sarcomeres grow [65,77]. We expressed UAS-GFP-Act88F starting around 56 h APF using Flightin-Gal4 (Fln-G4), which allowed us to monitor actin incorporation over the critical time period for hollow myofibril formation. In control sarcomeres from 90 h APF flies, we observed a box-like labeling pattern, reflecting GFP-Act88F integration both laterally as well as at the plus and minus ends of the thin filament (Fig 3H, 3H’ and 3H”, and S3B–S3B”‘ Fig and S3 Movie). In *bru1*^*M3*^ mutants, this pattern is significantly altered. GFP-Act88F is incorporated weakly at the z-disc and strongly and uniformly across the M-line (Fig 3I, 3I’ and 3I”, and S3C–S3C”‘ Fig and S4 Movie). This is striking, as sarcomeres do not grow appreciably in length in *bru1*^*M3*^ mutants, thus indicating a defect either in thin filament stability or capping protein function. Moreover, the strong lateral incorporation of GFP-Act88F coupled with exclusion from the central core of the sarcomere observed in control flies is not evident in *bru1*^*M3*^ (Fig 3H” and 3I”), even though mutant sarcomeres exhibit excessive radial growth from 60 h to 90 h APF (Fig 3D and 3E). We interpret this to reflect loose packing of the thin filament lattice allowing actin integration across the sarcomere, as well as radial integration of other unlabeled actin isoforms. These data reveal a clear defect in thin filament growth dynamics, and identify separable phenotypes in processes controlling growth in sarcomere length and width.

We next evaluated the localization of Mhc^Wee-P26^-GFP, to test if thick filament growth is also disrupted in *bru1*^*M3*^ myofibrils. In control sarcomeres, Mhc^Wee-P26^-GFP is localized in 2 central dots flanking the M-line (Fig 3J, 3J’ and 3J”, S3D and S3E–S3E”” Fig and S5 Movie), because a developmental switch in Mhc isoform expression results in incorporation of an unlabeled Mhc isoform after 48 h APF [66]. This isoform switch is partially impaired in *bru1*^*M3*^ IFM, so Mhc^Wee-P26^-GFP is continuously expressed [44], resulting in GFP incorporation across the thick filament (Fig 3K, 3K’ and 3K”, and S3F Fig). Interestingly, Mhc^Wee-P26^-GFP can be seen to form an irregular and asymmetric hook-like pattern at the M-line in *bru1*^*M3*^ sarcomeres (Fig 3K”, S3F and S3G–S3G”” Fig and S6 Movie), indicative of M-line misalignment or instability. We also observed myofibrils with 2 or more seemingly distinct Mhc^Wee-P26^-GFP dots (Fig 3K” and S6 Movie), suggesting that large, irregular myofibrils may result from myofibril fusion. Logically, neighboring myofibrils undergoing abnormal radial growth might grow into one another and fuse, contributing to the formation of hollow myofibril structures. To test this, we calculated the number of myofibrils per bundle in our cross-section data (Fig 3D) and found a significant reduction from 60 h to 80 h APF in *bru1*^*M3*^ but not in *w*^*1118*^ IFM (Fig 3L). Interestingly, this partial fusion of myofibrils does not reflect a switch in the fiber-type fate of the IFM, as the majority of tubular genes are not misexpressed in *bru1*^*M3*^ IFM (S3H Fig). Similarly, while there is misuse of tubular exons in *bru1*^*M3*^ IFM (S2D, S2E and S2O–S2Q Fig), many tubular exons are not strongly affected (S3H Fig), indicating that loss of Bru1 does not result in a complete switch in fiber-type-specific splicing, but rather loss of IFM-specific splice events. Taken together, our data support a mechanism whereby altered expression ratios of actins and cytoskeletal regulators impairs sarcomere growth in length, but concurrently promotes aberrant radial growth leading to myofibril fusion and the formation of hollow myofibrils during myofibril maturation in *bru1*^*M3*^ IFM.

### Developmental up-regulation of fibrillar genes is impaired in *bru1-IR* IFM

**Fig 4:**
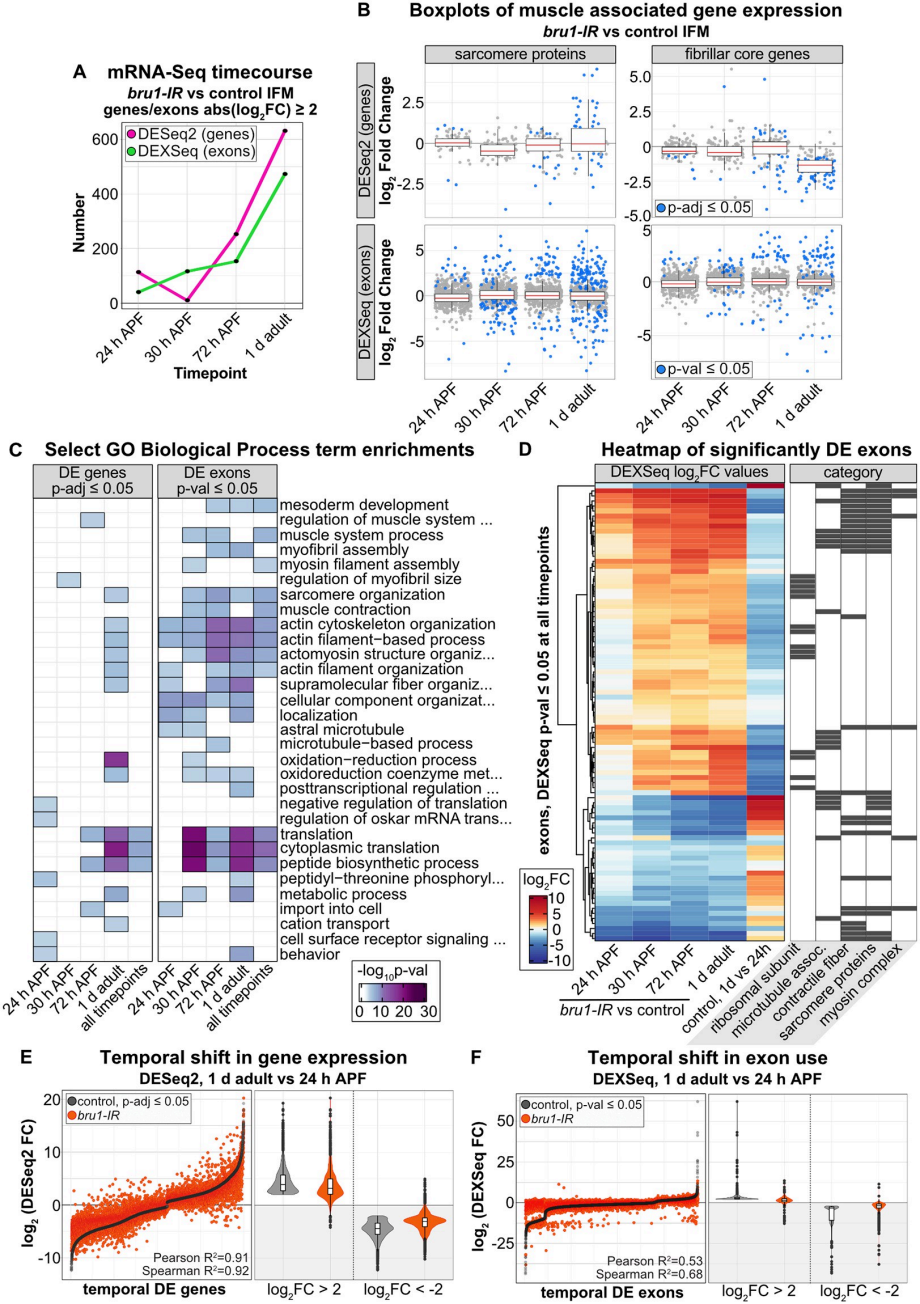
Knockdown of *bru1* disrupts temporal dynamics of gene expression and alternative splicing necessary for maturation of flight muscle. **(A)** Plot of the number of genes (magenta) and exons (green) significantly differentially expressed (*p* ≤ 0.05, abs(log_2_FC) ≥ 2) in *bru1-IR* versus control IFM at 24 h, 30 h, 72 h APF, and in 1 d adult. **(B)** Boxplot of changes in gene expression (DESeq2) and exon use (DEXSeq) in sarcomere proteins and fibrillar muscle genes across the *bru1-IR* IFM time course. Blue dot denotes *p* ≤ 0.05. **(C)** Heatmap of select Biological Process GO term enrichments in significantly regulated genes (DESeq2, p-adj ≤ 0.05) and exons (DEXSeq, p-val ≤ 0.05) in *bru1-IR* IFM at 24 h, 30 h, 72 h APF, or in 1 d adult, or at all 4 time points. **(D)** Heatmap (left) of all exons significantly DE (DEXSeq, p-val ≤ 0.05) at all time points in *bru1-IR* versus control IFM. The fifth column shows the temporal change in use of the same exons in *w*^*1118*^ IFM from 24 h APF to 1 d adult. Exons are identified as belonging to ribosomal subunit, microtubule associated, contractile fiber, sarcomere proteins, or myosin complex gene categories (right, black boxes). **(E)** Left: Plot of the log_2_FC values of all genes differentially expressed (DESeq2, p-adj ≤ 0.05) in *w*^*1118*^ IFM (black dots) from 24 h APF to 1 d adult, ordered by control log_2_FC, and the corresponding change in the same genes in *bru1-IR* IFM (orange dots). Right: Violin plots comparing control (gray) and *bru1-IR* (orange) expression of strongly up-regulated (log_2_FC ≥ 2) and down-regulated (log_2_FC ≤ -2) temporal-switch genes. **(F)** Left: Plot of the log_2_FC value of all exons differentially expressed (DEXSeq, p-val ≤ 0.05) in *w*^*1118*^ IFM (black dots) from 24 h APF to 1 d adult, and the corresponding change in those same exons in *bru1-IR* IFM (orange dots). Right: Violin plots comparing exon use of strongly up-regulated (log_2_FC ≥ 2) and down-regulated (log_2_FC ≤ -2) temporal-switch exons in control (gray) and *bru1-IR* (orange) IFM. Underlying data can be found in S3 Table and the RNA-Seq data tables as listed in S6 Table. APF, after puparium formation; GO, gene ontology; IFM, indirect flight muscle.

While investigating fiber-type-specific exon misregulation, we noticed that genes and exons that are normally expressed preferentially in mature IFM are down-regulated in *bru1*^*M3*^ IFM (S3I Fig). As CELF1 is known to promote embryonic splice events in vertebrate muscle [78], we wondered if developmentally regulated transitions in gene expression and splicing are also disrupted in IFM lacking Bru1. To explore temporal-dependent dynamics in gene expression and exon use, we analyzed an mRNA-Seq time course in control and *bru1-IR* muscle at 24 h, 30 h, and 72 h APF and 1 d adult. We confirmed via qPCR that *bru1-IR* (Mef2-Gal4 driven RNAi against *bru1*) results in a significant, 56-fold decrease in *bru1* mRNA levels in IFM (S7A Fig). We noted that at all 4 time points, we could detect significant changes in gene expression and exon use in *bru1-IR* IFM (Fig 4A and S4A Fig and S3 Table). There was a dramatic increase in the number and magnitude of changes at both the gene and exon level in *bru1-IR* at 72 h APF and 1 d adult (Fig 4A and S4A Fig), which mirrors the increase in severity of cellular phenotypes in *bru1*^*M3*^ IFM from 80 h APF to 1 d adult (Figs 1A, 1F, 3D and 3G). These results show that the number and severity of gene and exon regulatory defects progressively increases in *bru1-IR* IFM as development proceeds.

*Drosophila* IFM has been shown to undergo a developmental transition in gene expression that is necessary to establish the stretch-activation mechanism characteristic of mature flight muscle [42]. We next evaluated if this transition is disrupted in *bru1-IR* IFM. We evaluated specific subsets of genes previously shown to change across IFM development, including SPs, fibrillar muscle genes, and actin cytoskeleton genes. While we saw down-regulation of individual SPs and cytoskeleton genes, almost all core fibrillar muscle genes are strongly down-regulated in 1 d adult *bru1-IR* IFM (Fig 4B, S4B and S4C Fig). This is also reflected in GO term enrichments across the time course. On the gene expression level, at pupal time points we found enrichment for terms such as “negative regulation of translation,” “cell surface receptor signaling pathway,” and “behavior” at 24 h APF, “regulation of myofibril size” at 30 h APF, and “regulation of muscle system development” and “translation” at 72 h APF (Fig 4C). In 1 d adult bru1-IR IFM, we saw enrichment for cytoskeletal and muscle terms such as “sarcomere organization,” “actin cytoskeleton organization,” and “actomyosin structure organization,” as well as terms related to metabolism and translation such as “oxidation-reduction process,” “cytoplasmic translation,” and “metabolic process.” To test genome-wide if this switch in gene expression during IFM maturation is disrupted, we identified all genes in our mRNA-Seq time course that are significantly regulated between 24 h APF and 1 d adult in control IFM (adjusted *p*-value <0.05), and plotted their temporal change in expression in *bru1-IR* IFM (Fig 4E). We found that developmental changes in gene expression in *bru1-IR* IFM are correlated with those observed in control IFM (correlation value = 0.9107848), suggesting that *bru1-IR* IFM does undergo a temporal switch in gene expression. However, the magnitude of up- or down-regulation is often reduced in *bru1-IR* IFM, and a subset of genes shows regulation in the opposite direction between 24 h APF to 1 d adult (Fig 4E and S4A Fig), suggesting the switch is not as clean as in control IFM. We verified this switch in expression of Mlp60A and Mlp84B, which show strong up-regulation at the gene level in *bru1-IR* IFM from 72 h APF to 1 d adult (S5A Fig), using GFP fosmid reporters (S5F and S5G Fig). We conclude that loss of Bru1 selectively impairs up-regulation of fibrillar muscle genes, but not the entire maturation program during IFM development.

### A developmental switch to mature splice isoforms is blocked in IFM lacking Bru1

Based on the developmental transition in gene expression, we hypothesized that a similar transition in alternative splicing exists in wild-type IFM. We next investigated if such a splicing transition exists, and if it is disrupted in IFM lacking Bru1. The Biological Process GO terms enriched in the hundreds of DE exons at all 4 time points in our mRNA-Seq time course (Fig 4A) included “actin cytoskeleton organization,” “actin filament-based process,” “actomyosin structure organization,” “sarcomere organization,” and “cytoplasmic translation” (Fig 4C). We therefore started by looking at exon use dynamics in core fibrillar muscle genes and SPs, as several mature, IFM-specific protein isoforms have been reported in these categories [14,66,79,80]. In total, at 24 h, 30 h, or 72 h APF or at 1 d adult, we saw significant changes in 222 exons from 73 core fibrillar muscle genes and 413 exons from 56 SPs in *bru1-IR* IFM (S4D Fig). Strikingly, exons that were up-regulated in *bru1-IR* IFM were down-regulated in control IFM between 24 h APF to 1 d adult, while exons that were down-regulated in *bru1-IR* IFM were up-regulated in control IFM between 24 h APF to 1 d adult.

To expand beyond SPs and fibrillar genes, we next identified a set of 91 exons that are significantly misregulated in at least 3 of 4 time points in *bru1-IR* IFM. These exons became more strongly misregulated as development proceeds and belonged to genes encoding ribosomal subunits, microtubule-associated genes, SPs, contractile fiber, and myosin complex genes (Fig 4D). When we looked at the temporal change in use of these exons in control muscle from 24 h APF to 1 d adult, we found that all of the exons up-regulated in *bru1-IR* IFM are normally down-regulated in 1 d adult muscle. Likewise, the exons down-regulated in *bru1-IR* IFM are normally up-regulated between 24 h APF and 1 d adult in control IFM (Fig 4D). We then identified all exons in our mRNA-Seq time course that are significantly regulated between 24 h APF and 1 d adult in control IFM (Fig 4F). We observed a clear temporal switch in exon use, reflecting mainly alternative splice events and alternative 3’ UTRs, but also alternative promoter use (S3 Table). Strikingly, when we plotted the temporal change in use of these exons in *bru1-IR* IFM, we observed a reduction in coordinated developmental regulation. We confirmed these mRNA-level changes at the protein level for *wupA*, *Kettin*, and *Clip190* using GFP-tagged reporters under the control of endogenous regulatory elements (S5A and S5C–S5E Fig). This analysis reveals that loss of Bru1 results in a block in the temporal shift in exon use during IFM development, including increased expression of tubular-preferential isoforms. Thus, the molecular defect that underlies the myofibril growth and hypercontraction defects observed during myofibril maturation in *bru1-IR* and *bru1*^*M3*^ IFM is a failure to transition to expression of mature, muscle-type-specific splice isoforms.

### Loss of Bru1 leads to cytoskeletal organization defects at early stages of myogenesis

**Fig 5:**
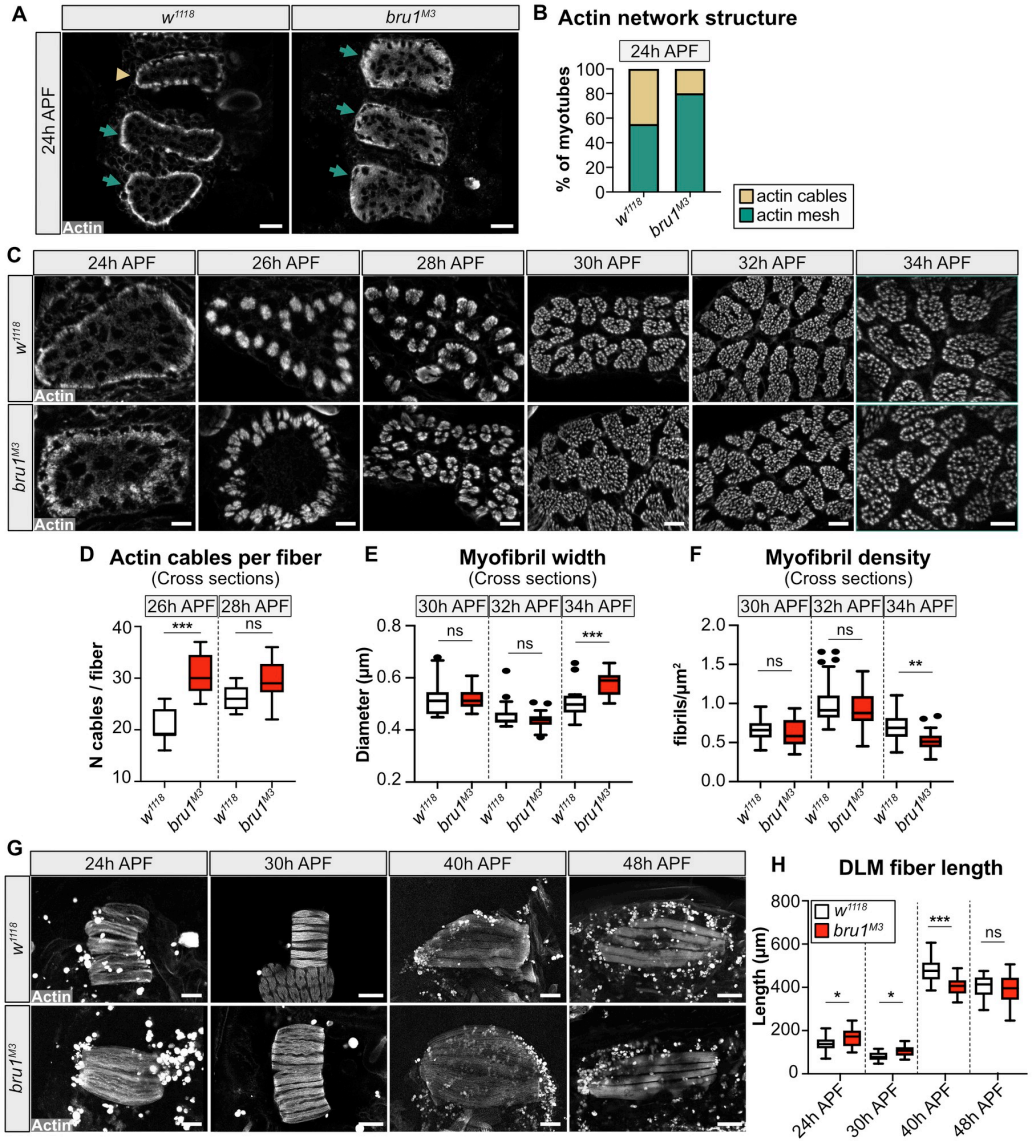
Early cytoskeletal rearrangement and myofiber compaction are abnormal in *bru1* mutant IFM. **(A)** Confocal images of DLM cross-sections at 24 h APF. Phalloidin stained F-actin (gray) reveals progressive condensation of the actin network into cables (arrow head) near the sarcolemma in *w*^*1118*^, but a meshwork (arrow) in *bru1*^*M3*^. Scale bar = 10 μm. **(B)** Quantification of actin network structure in (A). *N* > 10 for each genotype. **(C)** Cross-section time-course of early cytoskeletal rearrangements and pre-myofibril formation in *w*^*1118*^ and *bru1*^*M3*^ at 24 h, 26 h, 28 h, 30 h, 32 h, and 34 h APF. Scale bar = 5 μm. Irregular actin condensation at 24 h and cable splitting at 26 h and 28 h APF is evident in *bru1*^*M3*^, prior to pre-myofibril formation at 30–32 h APF. **(D)** Quantification of actin cable number per myotube in *w*^*1118*^ and *bru1*^*M3*^ at 26 h and 28 h APF. Significance determined by ANOVA and post hoc Tukey (ns, not significant; ***, p-val < 0.001). **(E, F)** Quantification of myofibril width (E) and density (F) in *w*^*1118*^ and *bru1*^*M3*^ at 30 h, 32 h, and 34 h APF. The first sarcomere growth defects in *bru1*^*M3*^ are already detected at 34 h APF, when the pre-myofibrils are fully formed. Significance determined by ANOVA and post hoc Tukey (ns, not significant; **, p-val < 0.01; ***, p-val < 0.001). All boxplots are shown with Tukey whiskers, outlier data points marked as black dots. **(G)** Confocal Z-stack images of DLMs in *w*^*1118*^ and *bru1*^*M3*^ at 24 h, 30 h, 40 h, and 48 h APF. DLM fibers undergo compaction at 30 h APF, followed by re-extension and fiber growth. Phalloidin stained actin, gray; scale bar at 24, 30 h APF = 50 μm, at 40, 48 h APF = 100 μm. **(H)** Quantification of the DLM fiber length in (G). *bru1*^*M3*^ DLM fibers fail to fully compact. Significance determined by ANOVA and post hoc Tukey (ns, not significant; *, p-val < 0.05; ***, p-val < 0.001). Underlying data can be found in Fig 5 Source Data as listed in S6 Table. APF, after puparium formation; DLM, dorsal-longitudinal myofiber; IFM, indirect flight muscle.

Although published Bru1 phenotypes in IFM are only reported after 48 h APF [14,36], our data above show that gene expression and splicing of structural genes are already abnormal in *bru1-IR* IFM from 24 h APF, prior to myofibril formation, and that abnormal sarcomere structure and contractility are already evident in *bru1*^*M3*^ myofibers at 48 h APF. We therefore investigated the possibility that Bru1 also has a function in early IFM development. We extended our cross-section time course to include time points at 24 h, 26 h, 28 h, 30 h, 32 h, and 34 h APF, a time window during which the developing IFM myofibers undergo dramatic cytoskeletal reorganization in preparation for myofibril formation from 30 to 34 h APF [81,82]. Already at 24 h APF, we detected a defect in actin cytoskeleton organization in *bru1*^*M3*^ myofibers (Fig 5A). In control *w*^*1118*^ IFM at 24 h APF, F-actin is tightly organized at the sarcolemma, while in *bru1*^*M3*^ myofibers it forms a more diffuse meshwork throughout the sarcoplasm that fails to condense as tightly as in wild type (Fig 5B and 5C). By 26 h APF in *w*^*1118*^ myofibers, F-actin organizes into uniformly distributed cables at the sarcolemma, which then subdivide and migrate toward the center of the myofiber from 28 to 30 h APF. In *bru1*^*M3*^ myofibers this process is abnormal, with F-actin organizing into many smaller, unequally distributed cables with impaired migration (Fig 5C and 5D). Both *w*^*1118*^ and *bru1*^*M3*^ myofibers undergo myofibrillogenesis from 30 to 34 h APF, but there are fewer myofibrils present in *bru1*^*M3*^ myofibers by 34 h APF and those myofibrils are significantly larger in diameter than *w*^*1118*^ myofibrils (Fig 5C, 5E and 5F). Progressive organization of the actin cytoskeleton during IFM myogenesis leads to myofiber compaction in addition to generating the tension necessary to drive myofibrillogenesis [63,73,74]. At both 24 h and 30 h APF, myofibers are longer in *bru1*^*M3*^ than in *w*^*1118*^ (Fig 5G and 5H), demonstrating that IFM compaction is mildly impaired and consistent with a cytoskeletal defect in myofibers lacking Bru1. We conclude that Bru1 is necessary during early IFM development to regulate cytoskeletal organization dynamics that support proper myofibril formation.

### Temporally restricted RNAi reveals a requirement for Bruno1 in early and late myogensis

**Fig 6:**
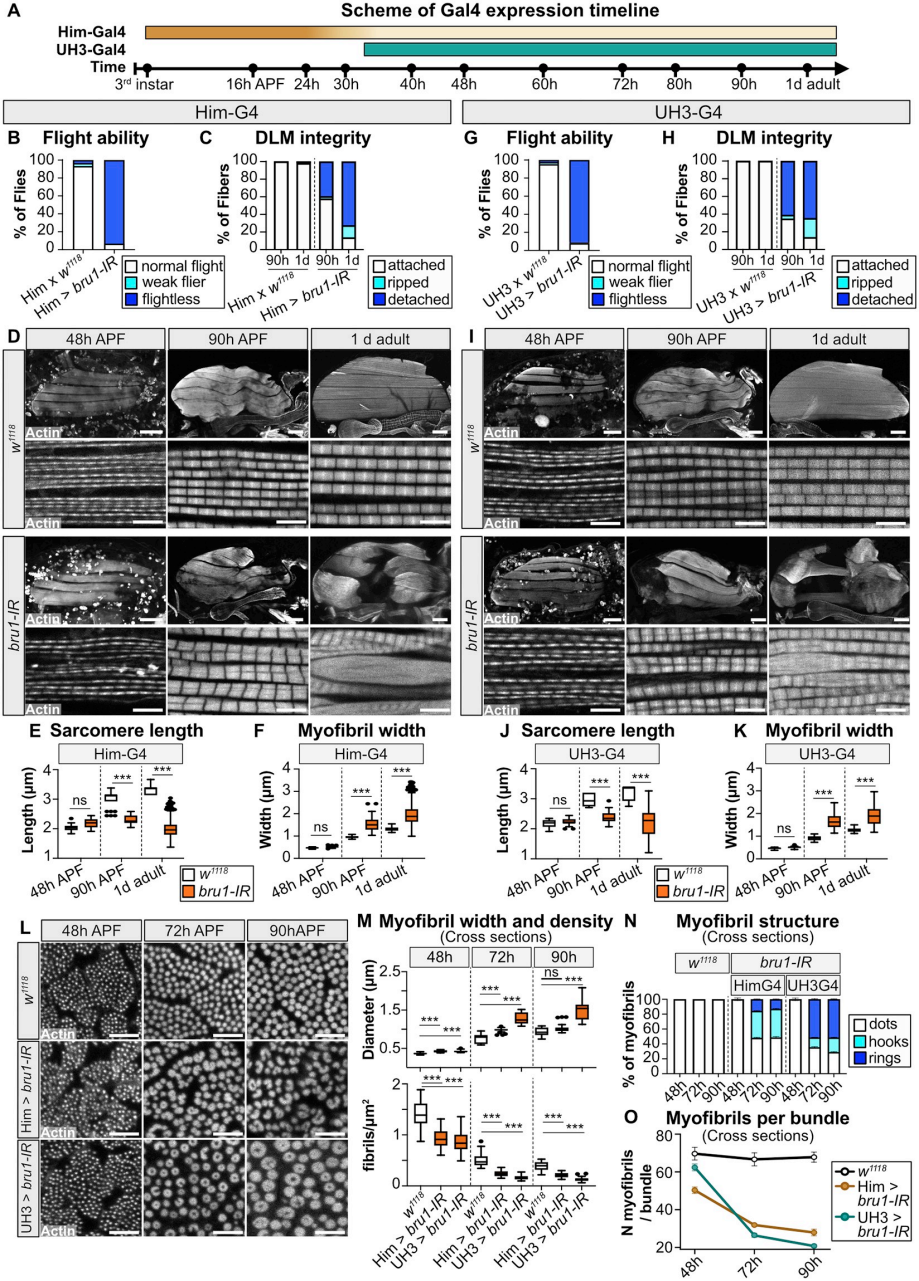
Temporally restricted RNAi demonstrates a functional requirement for Bru1 during early myogenesis. **(A)** Scheme of Him-Gal4 and UH3-Gal4 expression timing during IFM myogenesis. Gradient color of the bar indicates the strength of expression. Time points as marked. **(B)** Quantification of flight ability in *Him*-Gal4 driven *bru1-IR* (*bru1-IR*^*Him*^) (*N* > 130). **(C)** Quantification of myofiber ripping and detachment phenotypes in control and *bru1-IR*^*Him*^ at 90 h APF and 1 d adult (*N* > 40). **(D)** Confocal projections of control and *bru1-IR*^*Him*^ hemithoraxes (upper, scale bar = 100 μm) and single-plane images of myofibrils (lower, scale bar = 5 μm) in control (top) and *bru1-IR*^*Him*^ (bottom) at 48 h and 90 h APF and 1 d adult. **(E, F)** Quantification of sarcomere length (E) and myofibril width (F) from (D). Boxplots are shown with Tukey whiskers, outlier data points marked as black dots. Significance determined by ANOVA and post hoc Tukey (ns, not significant; ***, p-val < 0.001). **(G)** Quantification of flight ability in *UH3*-Gal4 driven *bru1-IR* (*bru1-IR*^*UH3*^) (*N* > 110). **(H)** Quantification of myofiber integrity in control and *bru1-IR*^*UH3*^ at 90 h APF and 1 d adult (*N* > 40). **(I)** Confocal projections of hemithoraxes (upper, scale bar = 100 μm) and single-plane images of myofibrils (lower, scale bar = 5 μm) in control (top) and *bru1-IR*^*UH3*^ (bottom) at 48 h and 90 h APF and 1 d adult. The severity of the *bru1*-RNAi associated myofibril phenotype is comparable between (D) and (I). **(J, K)** Quantification of sarcomere length (J) and myofibril width (K) from (I). Significance determined by ANOVA and post hoc Tukey (ns, not significant; ***, p-val < 0.001). **(L)** Single-plane cross-section images of DLM myofibrils in control *w*^*1118*^, *bru1-IR*^*Him*^, and *bru1-IR*^*UH3*^ at 48 h, 72 h, and 90 h APF. Scale bar = 5 μm. **(M)** Quantification of myofibril width (top) and density (bottom) in (L). Significance determined by ANOVA and post hoc Tukey (ns, not significant; ***, p-val < 0.001). **(N)** Quantification of myofibril structure from (L), showing the ratio of normal dot morphology (white) to abnormal hook (cyan) and ring (blue) structures. Fewer hooks and rings form in early expressed *bru1-IR*^*Him*^ as compared to *bru1-IR*^*UH3*^. Error bars = SEM. **(O)** Quantification of mean myofibril number per bundle in (L). *bru1-IR*^*UH3*^ IFM forms the correct number of myofibrils while *bru1-IR*^*Him*^ IFM does not, but myofibril fusion is more extensive after 48 h APF in *bru1-IR*^*UH3*^. *bru1-IR*^*UH3*^ IFM have a near wild-type number of 62 ± 12 myofibrils per bundle at 48 h APF, but by 72 h APF the number of 26 ± 6 myofibrils per bundle is less than the 32 ± 7 observed with *bru1-IR*^*Him*^. Error bars = SEM. F-actin in (D, I, L) stained with phalloidin. Underlying data can be found in Fig 6 Source Data as listed in S6 Table. APF, after puparium formation; DLM, dorsal-longitudinal myofiber; IFM, indirect flight muscle.

The data we present above suggest that Bru1 is required both during early and late stages of IFM myogenesis. To test the temporal requirement of Bru1 in muscle development, we performed RNAi with a panel of 5 previously characterized Gal4 enhancer lines with distinct temporal expression patterns in IFM (Fig 6A, S6A and S7 Figs). In contrast to Mef2-Gal4, which is expressed in all muscle throughout development, salm-Gal4, Act88F-Gal4, UH3-Gal4, and Fln-Gal4 are expressed in IFM from approximately 16 h APF, 24 h APF, 34 h APF, and 56 h APF, respectively [77,80,83,84]. Him-Gal4 is expressed in myoblasts and is down-regulated in IFM by 30 h APF [42]. *bru1-IR* led to a strong reduction in *bru1* mRNA with all drivers tested (S8A Fig), but did not impair adult viability or eclosion (S6B Fig). We then compared phenotypes of *bru1-IR* driven by Gal4 lines active during early myogenesis (*bru1-IR*^*Him*^, *bru1-IR*^*salm*^, *bru1-IR*^*Act88F*^) to those active after myofibrillogenesis is completed (*bru1-IR*^*UH3*^ and *bru1-IR*^*Fln*^).

Knockdown of *bru1* during early myogenesis with Him-Gal4, salm-Gal4, or Act88F-Gal4 resulted in severe behavioral and structural defects. We focus below on *bru1-IR*^*Him*^ as representative of *bru1* RNAi knockdown phenotypes during early myogenesis. *bru1-IR*^*Him*^ flies were flightless (Fig 6B), and the percent of detached IFM myofibers increased between 90 h APF and 1 d adult, reflecting a progressive hypercontraction phenotype (Fig 6C and 6D), and 1 d adult *bru1-IR*^*Him*^ myofibrils were torn and degenerate, and at both 90 h APF and 1 d adult sarcomeres were significantly shorter and wider than control sarcomeres (Fig 6D–6F). Him-Gal4 driven expression of UAS-GFP or UAS-GFP-RNAi did not affect sarcomere length or width (S7A–S7O Fig), confirming this phenotype is specific to knockdown of *bru1*. Interestingly, although Him-Gal4 turns off at 30 h APF, while salm-Gal4 and Act88F-Gal4 are expressed continuously starting from 16 h and 24 h APF, respectively, they all produce a similar *bru1-IR* phenotype characterized by short, thick sarcomeres and progressive hypercontraction mediated loss of adult IFM myofibers (Fig 6B–6F and S6C–S6G Fig). We confirmed that *bru1-IR*^*Him*^ results in a loss of Bru1 protein at 24 h and 30 h APF, indicating that RNAi is effective at early pupal stages, and from 48 h APF Bru1 protein is again detectable and presumably functional in the nuclei of *bru1-IR*^*Him*^ flies (S8M–S8Q Fig). This data shows that decreased expression of Bru1 during early stages of IFM development is sufficient to produce severe myofibril and sarcomere phenotypes.

We then examined *bru1* knockdown phenotypes with the UH3-Gal4 and Fln-Gal4 drivers that are expressed after myofibril formation. While *bru1-IR*^*UH3*^ produces flightless flies with ripped and detached IFM myofibers (Fig 6G–6I), *bru1-IR*^*Fln*^ flies are able to fly and have intact IFM (S6C, S6D, S6J-J’, and S6P-P’). Although *bru1* mRNA levels are significantly decreased by both *bru1-IR*^*UH3*^ and *bru1-IR*^*Fln*^ (S8A Fig), Bru1 protein is still detected in *bru1-IR*^*Fln*^ myofibers at 90 h APF, but not in *bru1-IR*^*UH3*^ myofibers (S8D–S8H Fig). This can be explained if Bru1 protein perdures for some time after RNAi is induced, or if Bru1 protein turnover decreases at later pupal stages. We therefore focused our subsequent analysis on *bru1-IR*^*UH3*^. Like we observed in *bru1-IR*^*Him*^ as well as in *bru1*^*M3*^ IFM, sarcomeres in *bru1-IR*^*UH3*^ IFM are shorter and thicker than control sarcomeres at 90 h APF and 1 d adult, and myofibrils are torn and display a progressive loss of sarcomere architecture (Fig 6I, 6J and 6K). However, when examined in cross-section, *bru1-IR*^*UH3*^ produces stronger radial growth defects than *bru1-IR*^*Him*^ (Fig 6L). *bru1-IR*^*UH3*^ myofibrils are significantly wider in diameter than control or *bru1-IR*^*Him*^ myofibrils at both 72 h and 90 h APF (Fig 6M), and proportionally more myofibrils appear as rings instead of hooks or dots already at 72 h APF (Fig 6N). In addition, although *bru1-IR*^*UH3*^ IFM have a near wild-type number of myofibrils per bundle at 48 h APF, a large drop in myofibril number is observed between 48 h and 72 h APF (Fig 6O). This suggests that the recovering level of Bru1 protein after 48 h APF in *bru1-IR*^*Him*^ promotes a somewhat less severe radial overgrowth phenotype than the absence of Bru1 protein from 48 h APF onwards in *bru1-IR*^*UH3*^. These data support a requirement for Bru1 during myofibril maturation after 48 h APF to regulate sarcomere growth dynamics.

### Levels of Bru1 expression during early myogenesis impact myofibril formation and growth

Our data indicate that phenotypes related to Bru1 function in early and late myogenesis may be separable, where early function defines the number and organization of myofibrils while later function regulates sarcomere radial and lateral growth. To test this hypothesis, we performed temporally regulated rescue experiments, as continuous Bru1 overexpression with Mef2-Gal4 causes severe phenotypes [14,36]. We generated a UAS-Bru1 (isoform A) rescue construct (S9A Fig), and then optimized expression conditions with our Gal4 panel (S9B–S9P Fig). Overexpression with Him-Gal4 and Fln-Gal4, the 2 Gal4 drivers with the narrowest expression windows, produced surviving flies with intact IFM (S9B–S9D Fig) and enabled us to evaluate Bru1 function in the early 0 to 30 h APF and late 56 h APF to adult time windows, respectively.

**Fig 7:**
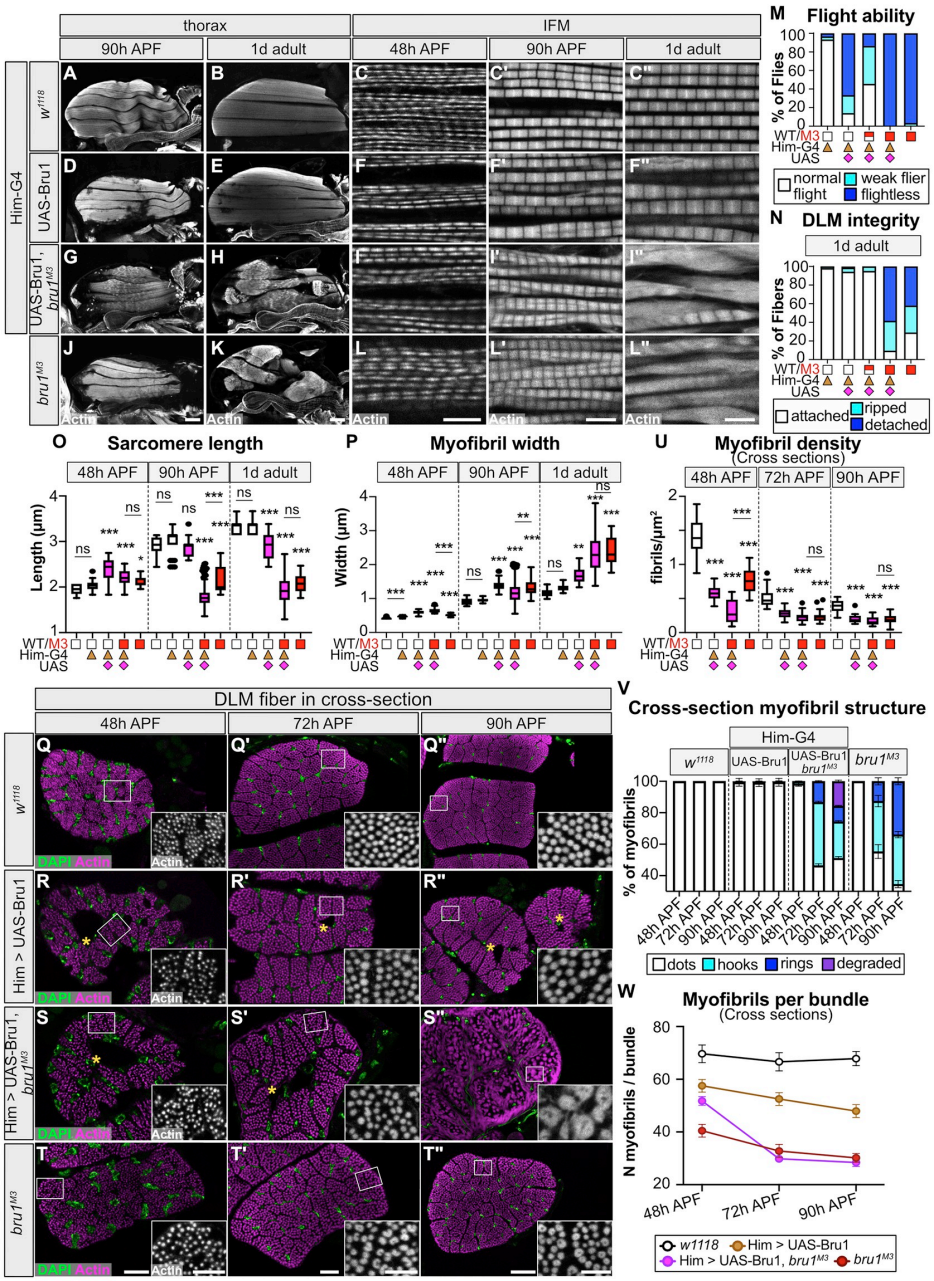
Expression of Bru1 restricted to early myogenesis fails to rescue and exacerbates *bru1*^*M3*^ myofibril phenotypes. **(A–L”)** Confocal projections of hemithorax (scale bar = 100 μm) and single-plane images of myofibril structure (scale bar = 5 μm) in control (A–C”); Bru1 overexpression (*Him*-Gal4 > UAS-Bru1, D–F”); early temporal rescue (*Him*-Gal4 > UAS-Bru1, *bru1*^*M3/M3*^, G–I”); and mutant *bru1*^*M3*^ (J–L”). Time points at 48 h, 90 h APF and 1 d adult as labeled. Phalloidin stained actin, gray. **(M, N)** Quantification of flight ability (M) and DLM fiber integrity (N) in (B, E, H, K). Genotypes are denoted by symbols: wild type, white square; *bru1*^*M3*^, red square; *Him*-Gal4, tan triangle; UAS-Bru1, magenta diamond. *N* > 40 fibers for each genotype. **(O, P)** Quantification of sarcomere length (O) and myofibril width (P) in (C, F, I, L). Boxplots are shown with Tukey whiskers, outlier data points marked as black dots. Significance determined by ANOVA and post hoc Tukey (ns, not significant; *, p-val < 0.05; **, p-val < 0.01; ***, p-val < 0.001). **(Q–T”)** Single-plane cross-section images of myofibril structure at 48 h, 72 h, and 90 h APF in control (Q–Q”), Bru1 overexpression (R–R”), early temporal rescue (S–S”), and mutant *bru1*^*M3*^ IFM (T–T”). Magnified image of selected area (white rectangle) shown in lower right corner. Overexpression of Bru1 with *Him*-Gal4 produces holes in the center of IFM myofibers (yellow asterisks) and cannot rescue later formation of hollow myofibrils; DAPI, green; phalloidin stained actin, magenta; scale bar = 10 μm (Q–T, Q’–T’), 20 μm (Q”–T”) and 5 μm (zoom-in sections). **(U, V)** Quantification of myofibril density (U) and myofibril structure (V) in (Q–T”). Genotypes, boxplot, and significance as above. Error bars in V = SEM. **(W)** Quantification of mean myofibril number per fiber bundle in (Q–T”). *Him*-Gal4 driven Bru1 can partially rescue the number of myofibrils formed in *bru1*^*M3*^ myofibers before 48 h APF, but cannot rescue myofibril fusion and hollow myofibril formation after 48 h APF. Error bars = SEM. Underlying data can be found in Fig 7 Source Data as listed in S6 Table. APF, after puparium formation; DLM, dorsal-longitudinal myofiber; IFM, indirect flight muscle.

To evaluate Bru1 function during early IFM development, we performed overexpression and rescue experiments with Him-Gal4. We first confirmed that in a *bru1*^*M3*^ background, Him-Gal4 drives expression of Bru1 at 24 h APF, but not at 48 h or 90 h APF (S10A and S10B Fig), demonstrating the temporal-restriction of Bru1 expression to early myogenesis. Overexpression of Bru1 in a wild-type background is sufficient to produce short, thick sarcomeres and loss of flight ability, but IFM myofibers remain attached (Fig 7A–7F and 7M–7P, and S10C–S10F Fig). This demonstrates that overexpression of Bru1 produces the same sarcomere phenotype as loss-of-function, indicating that Bru1 is dosage sensitive. In cross-sections, overexpression of Bru1 with Him-Gal4 leads to a reduced myofibril density, a decreased number of myofibrils per bundle and myofibrils of variable thickness at 72 h and 90 h APF, but is not sufficient to produce hollow myofibrils (Fig 7Q, 7R, 7V and 7W, and S10I Fig). Unexpectedly, IFM overexpressing Bru1 have a prominent hole in the middle of the myofiber at 48 h APF (Fig 7R–7R”), which we interpret to reflect failed migration of actin cables to the center of the fiber from 28 to 30 h APF (Fig 5C). These data demonstrate that the dosage of Bru1 before 30 h APF is crucial to regulate the number and regularity of myofibrils and can further influence sarcomere growth during later developmental stages.

We then investigated if Him-Gal4 mediated expression of Bru1 is sufficient to rescue the *bru1*^*M3*^ phenotype. Instead of rescuing, expression of Bru1 during early developmental stages increased the severity of the *bru1*^*M3*^ phenotype. Rescue flies are flightless and a show greater proportion of flies with ripped or detached IFM at both 90 h APF and 1 d adult than *bru1*^*M3*^ (Fig 7G–7N, S10C and S10D Fig). Sarcomeres in rescue IFM are significantly shorter than both control and *bru1*^*M3*^ sarcomeres at 90 h APF (Fig 7O), are as wide as mutant sarcomeres (Fig 7P), and show myofibril tearing and loss of sarcomere architecture in 1 d adults (Fig 7G–7I”). In cross-section, Him-Gal4 > Bru1 rescue flies form hollow myofibril structures that are larger and more irregular than those formed by *bru1*^*M3*^ myofibrils at 90 h APF (Fig 7S–7T”). Interestingly, while the myofibril density and developmental progression of hollow myofibril formation mirrors that observed in *bru1*^*M3*^ (Fig 7U and 7V), rescue flies have more myofibrils per bundle at 48 h APF than *bru1*^*M3*^ (Fig 7W), indicating that Bru1 expression may partially rescue some early myofibril defects. Rescue flies also have a hole in the middle of the IFM myofiber that is evident at 48 h, 72 h, and 90 h APF (Fig 7S–7S”). We confirmed the presence of this hole using hematoxylin and eosin staining, as well as the absence of such a hole in wild-type or *bru1*^*M3*^ myofibers (S10G and S10H Fig). Taken together, these data demonstrate: (1) that Bru1 has a function during early stages of IFM myogenesis; (2) myofibril phenotypes are sensitive to Bru1 expression level before 48 h APF; (3) loss and gain of Bru1 can produce similar phenotypes; and (4) misregulation of Bru1 in early development also impacts later stages of myofibril maturation.

### Rescue of Bru1 during late development is insufficient to rescue myofibril structural defects

**Fig 8:**
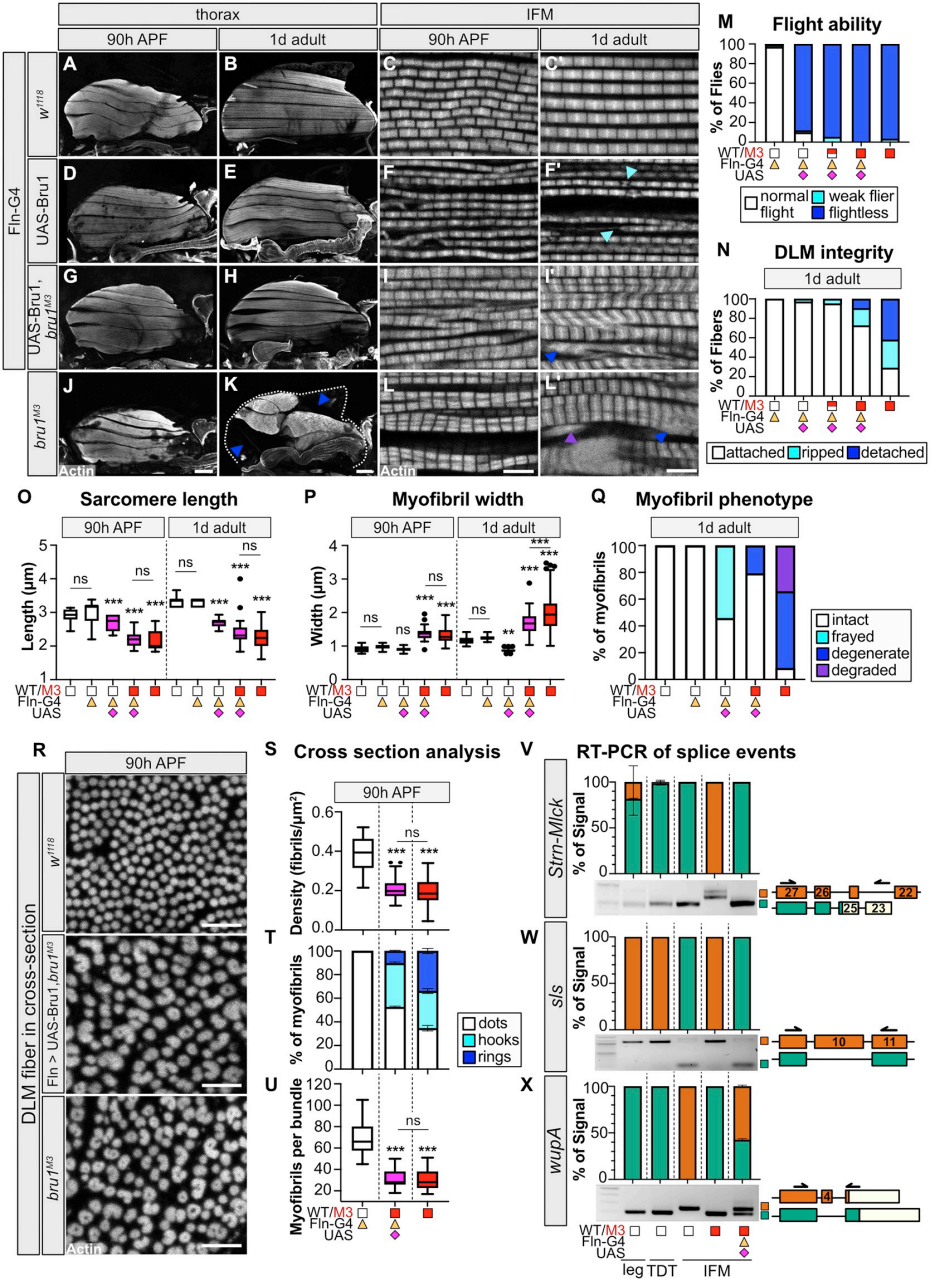
Expression of Bru1 restricted to late myogenesis partially rescues *bru1*^*M3*^ myofibril phenotypes and restores alternative splicing defects. **(A–L’)** Confocal projections of hemithorax and single-plane images of myofibril structure at 90 h APF and 1 d adult in control (A–C’); Bru1 overexpression (*Fln*-Gal4 > UAS-Bru1, D-F’); late temporal rescue (*Fln*-Gal4 > UAS-Bru1, *bru1*^*M3/M3*^, G-I’); and mutant *bru1*^*M3*^ (J–L’). The severity of myofiber detachment and torn myofibril phenotypes is partially rescued in *Fln*-Gal4 > UAS-Bru1, *bru1*^*M3*^ IFM. Dashed line outlines the thorax boundary in (K). Frayed (cyan), degenerate (blue), or degraded (purple) myofibrils, arrowheads; Phalloidin stained actin, gray; scale bar = 100 μm (thorax); scale bar = 5 μm (IFM). **(M, N)** Quantification of flight ability (M) and DLM fiber integrity (N) in (B, E, H, K) (*N* > 50). Genotypes are denote by symbols: wild type, white square; *bru1*^*M3*^, red square; *Fln*-Gal4, orange triangle; UAS-Bru1, magenta diamond. **(O, P)** Quantification of sarcomere length (O) and myofibril width (P) in (C–L’). Boxplots are shown with Tukey whiskers, outlier data points marked as black dots. Significance determined by ANOVA and post hoc Tukey (ns, not significant; **, p-val < 0.01; ***, p-val < 0.001). **(Q)** Quantification of myofibril phenotypes present in (C’–L’) (N > 70). **(R)** Single-plane cross-section images of myofibril structure in DLM from control, late temporal rescue, and mutant *bru1*^*M3*^ at 90 h APF. Phalloidin stained actin, gray; scale bar = 5 μm. **(S–U)** Quantification of cross-section myofibril density (S), myofibril structure (T), and number of myofibrils per bundle (U) in (R). Fewer hollow myofibrils (rings) develop in *Fln*-Gal4 > UAS-Bru1, *bru1*^*M3*^ IFM by 90 h APF. Significance determined as above, error bars in (T) = SEM. **(V–X)** Semi-quantitative RT-PCR verification of Bru1 regulated alternative splice events in *Strn-Mlck* (V), *sls* (W), and *wupA* (X). Representative gel images and quantification of percent exon use in control fibrillar IFM, tubular leg and jump (tergal depressor of the trochanter, TDT) muscle, and mutant *bru1*^*M3*^ or late rescue *Fln*-Gal4 > UAS-Bru1, *bru1*^*M3*^ IFM. Error bars = SD. Scheme on the right of alternative isoforms with primer locations, color coding consistent between scheme and bar plot. 3′-UTR regions in light beige. Exon numbering according to FB2021_05. Underlying data can be found in Fig 8 Source Data and Gels as listed in S6 Table. APF, after puparium formation; DLM, dorsal-longitudinal myofiber; IFM, indirect flight muscle.

To evaluate Bru1 function during late IFM development, we performed overexpression and rescue experiments with Fln-Gal4. We first verified by antibody staining that at 90 h APF in a *bru1*^*M3*^ background, Fln-Gal4 drives expression of UAS-Bru1 (S1A and S1B Fig). Overexpression of Bru1 with Fln-Gal4 in a wild-type background was sufficient to produce flightless flies, but myofibers remained attached and did not display hypercontraction-related tearing (Fig 8A–8F’ and S11C Fig). Although myofibril and sarcomere structure were largely intact, sarcomeres were significantly shorter and thinner than in the control and 52.4% were frayed in 1 d adults (Fig 8A–8F’ and 8O–8Q, and S11C–S11E Fig). We conclude that overexpression of Bru1 during late IFM development with Fln-Gal4 is sufficient to produce flightlessness due to mild sarcomere defects, but not strong enough to reproduce the *bru1*^*M3*^ phenotype.

We then evaluated if expression of Bru1 from 56 h APF with Fln-Gal4 can rescue the *bru1*^*M3*^ phenotype. Strikingly, whereas 71.0% of myofibers are ripped or detached in 1 d adult *bru1*^*M3*^ flies, only 27.4% are ripped or detached in Fln-Gal4 rescue flies (Fig 8A–8L’ and 8N). In addition, while 91.5% of myofibrils are degraded, degenerate, or frayed in *bru1*^*M3*^ IFM, only 20.7% of myofibrils are affected in the rescue (Fig 8Q). However, the rescue flies are still flightless and their sarcomeres are also significantly shorter and thicker than control sarcomeres (Fig 8L’, 8M, 8O and 8P). Cross-section analysis revealed that hollow myofibril formation in the Fln-Gal4 rescue flies proceeds to the same extent as in *bru1*^*M3*^ IFM, and myofibril density and diameter, the percent of myofibrils converted to hook and ring-like structures, as well as the number of myofibrils per bundle is not significantly different from the *bru1*^*M3*^ mutant (Fig 8R–8U and S10F Fig). This indicates that expression of Bru1 with Fln-Gal4 is sufficient to produce a partial rescue of hypercontraction-related myofiber tearing and loss of sarcomere architecture, but is insufficient to rescue sarcomere and myofibril growth defects. To further test this conclusion, we performed semi-quantitative RT-PCR to validate the rescue of individual alternative splice events we have previously shown to be regulated by Bru1 [14,43]. We found that events in *Strn-Mlck*, *sls*, *Tm1*, and *Mhc* are completely rescued (Fig 8V and 8W, S11H and S11I Fig), while events in *wupA* and *Zasp52* are partially rescued (Fig 8X and S11G Fig). We conclude that expression of Bru1 with Fln-Gal4 is sufficient to restore Bru1-mediated splicing of key structural genes and partially alleviate hypercontraction-related phenotypes seen in *bru1*^*M3*^ IFM. However, this late-stage rescue cannot repair preexisting cytoskeletal structural and growth defects leading to a continued imbalance in growth in sarcomere length and width, abnormal radial growth of the myofibril, and associated functional deficits.

## Discussion

CELF proteins are important RNA regulators in muscle of both vertebrates and insects. Here, employing the power of the *Drosophila* model system, we have shown that Bru1 is required for multiple processes at different stages of muscle development. We propose the following developmental model integrating data from experiments with *bru1* mutants, *bru1-IR* and Bru1 overexpression (S12 Fig) to explain how misregulation of CELF proteins leads to muscle structural defects. In the developing myotube and nascent myofiber, Bru1 is required to promote cytoskeletal rearrangements that influence the number, organization, and size of myofibrils that will be formed (Figs 1, 3 and 5). Bru1 regulates isoform expression patterns that influence myofiber compaction dynamics during myofibrillogenesis, as well as the frequency and strength of spontaneous contractions after myofibril formation (Figs 2, 4 and 5). As development proceeds, Bru1 enables a genome-wide switch to production of mature isoforms in structural and cytoskeletal regulatory genes (Fig 4). In *bru1* mutants, disruption of this switch leads to an imbalance in sarcomere growth in length and width and a pronounced misregulation of radial growth that alters the dynamics of actin and myosin incorporation into sarcomeres and drives myofibril fusion and the formation of hollow myofibrils (Figs 1, 3, 6 and 7, and S12 Fig). Further, loss of IFM-specific isoforms produces abnormal actomyosin contractility throughout myofiber development and a terminal hypercontraction phenotype that results in adult myofiber loss (Fig 1). Due to this developmental progression, rescue of Bru1 during late development can restore alternative splice events and partially alleviate hypercontraction and myofiber loss, but cannot correct preexisting structural defects to restore sarcomere structure or muscle function (Figs 7 and 8). Our model provides novel insight into the multiple stages of myofibrillogenesis regulated by Bru1 and the breadth of the fiber-type differentiation processes regulated by CELF proteins.

Myofibril assembly and sarcomere growth require the coordination of multiple cellular processes. In early myogenesis, major cytoskeletal rearrangements, subdivision of the sarcoplasm and reorganization of internal membranes from 26 to 30 h APF immediately precede myofibrillogenesis at 30 to 32 h APF [81,82]. During myofibrillogenesis, pre-assembled complexes of actin thin filaments, α-actinin and short, bipolar myosin filaments are progressively organized into regularly patterned immature myofibrils [57,73,85–88] in a tension-dependent manner [63,74,89,90]. These processes are linked, as the sarcoplasm is patterned by organized arrays of microtubules that scaffold and direct actomyosin cytoskeletal assembly [82,91]. Consistent with previous studies, our data show that reorganization of the cytoskeleton is necessary for myofibrillogenesis and further identify a requirement for CELF activity during this process (Fig 5). While early cytoskeletal rearrangement defects in *bru1* mutant IFM at least partially reflect missplicing of actin regulators and sarcomere components (Figs 1–4), we speculate based on our transcriptomics analysis (Fig 4) that regulation of the microtubule cytoskeleton may also be defective in *bru1* mutants and another mechanism that contributes to myofibril defects. Microtubule association with developing myofibrils pre-patterns myofibril formation and facilitates radial myofibril growth during later sarcomere development [82], 2 of the key processes we identified as impaired in *bru1*^*M3*^ IFM (Figs 1 and 5). In cells, detyrosinated microtubules have been identified as an early marker of myoblast differentiation and are important for myofibril formation [91,92], but an increased density of persistent detyrosinated microtubules in dystrophic mouse muscle is associated with myofibrillar malformations [93,94]. Malformations likely arise due to disrupted protein synthesis, as microtubules are essential for mRNA transport and delivery to sites of protein translation at Z-discs in developing myofibers [95]. CELF2, 3, and 4 have been shown to regulate the splicing of multiple exons in microtubule-interacting proteins including tau, and changes in tau isoform expression and microtubule organization may contribute to neurological phenotypes in DM1 patients [96–99]. Future work will be necessary to assay microtubule network dynamics in muscle with altered CELF expression and to determine if microtubule regulatory defects contribute to cytoskeletal organization, myofibril assembly, or sarcomere growth deficits in *bru1*^*M3*^ IFM.

Myofibril development is also influenced by interactions with cellular organelles, notably mitochondria. Disruption of the microtubule network leads to contractile dysfunction in part because microtubules help position and mediate contact between myofibrils, sarcoplasmic reticulum, and mitochondria [100]. Mitochondrial networks have distinct morphologies in different muscle types [101] and in addition to long-established roles in metabolism, are necessary for muscle organization, morphogenesis, differentiation, and maintenance [45,102,103]. In IFM, mitochondria wrap around developing myofibrils and promote a fibrillar organization by preventing lateral alignment and myofibril fusion [101]. In turn, mitochondria are squeezed by myofibril crowding, activating a mechanical feedback mechanism that induces mitochondrial gene expression [42,104] to increase cristae density [105], thus increasing respiratory capacity [45]. Our transcriptomic and proteomic data reveal changes in mitochondrial gene expression that become more pronounced during later pupal stages (Fig 4), which may indicate that mitochondrial function and mechanical feedback mechanisms are impaired in *bru1* mutant muscle. Consistent with this observation from flies, DM1 patients display a number of metabolic defects and hallmarks of mitochondrial dysfunction including reduced expression of Coenzyme Q10, lower ATP production, and impaired mitochondrial dynamics and energy homeostasis [106–109]. Disruption of mitochondrial networks could promote the radial growth and partial-fusion of myofibrils observed in *bru1* mutant muscle as well as contribute to the observed defects in developmental gene expression, but further studies will be necessary to determine if mitochondrial dysfunction contributes to the IFM phenotype.

In addition to regulation of the microtubule and mitochondrial networks, developmental shifts in sarcomere protein isoform expression are thought to play important roles in myofibril assembly and function [13,18]. For example, Z-disc width in IFM is regulated by a developmental shift in the balance of long and short Zasp52 isoforms. Long isoforms of Zasp52 with multiple LIM domains promote Zasp protein oligomerization and sarcomere radial growth, while short Zasp52 isoforms with one or no LIM domain block oligomerization and restrict Z-disc growth [79]. Similarly, short isoforms of Fhos that localize to the M-line are sufficient to support early steps in sarcomere assembly and rescue the viability of *Fhos* null flies, but long isoforms of Fhos with an N-terminal extension that mediates Z-disc localization are required in IFM for sarcomere growth and maturation [65]. Other developmentally regulated shifts in exon use in sarcomeric genes such as vertebrate cardiac Troponin T (cTnT) [110,111], *Drosophila* Myosin heavy chain (Mhc) [112], and Titin-like proteins [113,114] have been shown to affect myofibril assembly, growth, and contractility. Here, we identified a genome-wide developmental switch in exon use and gene isoform expression in IFM that is disrupted in *bru1-IR* IFM (Fig 4). This switch is extensive and conservatively involves around 3,000 exons from 2,000 genes (S3 Table), including cytoskeletal and sarcomeric proteins, metabolic and mitochondrial genes, calcium regulation, signaling and the translation machinery. This reflects the diversity of cellular processes that must be coordinately regulated for the construction of functional muscle. RNA-sequencing technologies have revealed similar temporal shifts from embryonic to postnatal splice isoform expression in hundreds of cytoskeletal, calcium-regulatory, and signaling genes in vertebrate heart and skeletal muscles [21,27,115], suggesting that regulation of RNA processing is a conserved mechanism to fine-tune cytoskeletal dynamics, cellular metabolism, intracellular transport, and protein expression during muscle development.

While CELF1 and CELF2 are required during early mouse and chick myogenesis to promote embryonic splicing patterns in heart and skeletal muscle [18,19,21,116], previous studies on Bru1 in *Drosophila* IFM only reported phenotypes after 48 h APF during later phases of myofibril maturation [14,36]. Here, we showed that Bru1 in flies also has a function in early IFM myogenesis before 48 h APF to regulate cytoskeletal rearrangement and myosin contractility (Figs 4–8), demonstrating a conserved requirement for CELF family function during early myogenesis. Our data show that like other CELF proteins, Bru1 regulates alternative splicing of hundreds of exons (Fig 4). In addition to a nuclear role in alternative splicing, CELF proteins have been shown to have cytoplasmic roles in regulating mRNA decay and translation [18,38,117,118]. Based on the low or anti-correlation between mRNA-Seq and whole proteome mass spectrometry data for many peptides, our data also suggests a possible role for Bru1 in regulating mRNA stability or translation in IFM (Fig 1 and S1 Fig). Further studies are required to determine if this effect is direct, driven by changes in alternative exon or 3′-UTR use in target genes, or indirect through interaction with other RBPs such as Rbfox1 [43]. In vertebrates, down-regulation of CELF1 and concurrent up-regulation of MBNL1/2 promotes a transition to mature patterns of alternative splicing [21,23,24,119]. It is still unclear if the same temporal regulatory relationship exists between Bru1 and Muscleblind (mbl) in *Drosophila*. Mbl is expressed in both larval and adult muscle, is necessary for z-disc and myotendinous junction maturation in embryonic muscle, and regulates both sarcomere growth and myosin contractility [13,120–122], but the temporal requirement for Mbl function in IFM as well as Mbl target genes remain to be determined. Our discovery of an early requirement for Bru1 in IFM development strengthens the use of *Drosophila* as a model to examine the molecular mechanism of CELF function as well as co-regulatory interactions with other RBPs.

The function of Bru1 prior to myofibrillogenesis as well as during stages of sarcomere growth and myofibril maturation (Figs 1–6) demonstrates a requirement for Bru1 throughout IFM development. This is consistent with previous work showing that Bru1 is expressed selectively and continuously in the IFM and promotes fiber-type-specific splice events in structural genes necessary to establish adult IFM contractile properties [14,36,43]. Muscle-specific splicing factors are also found in vertebrates, for example RBM24 [123,124] or the Fragile-X like protein isoform FXR1P82,84 [125], and enhance splicing or stability of select isoforms of cytoskeletal genes to promote muscle differentiation [124,126,127]. Vertebrate CELF proteins are not expressed in a myofiber-type-specific manner, but they may have temporally regulated or fiber-type-specific functions. Overexpression of either CELF1, which is normally down-regulated during muscle differentiation, or a dominant-negative CELF1 protein is sufficient to change the ratio of slow to fast fiber types [29,128]. While CELF1 is down-regulated during differentiation, expression of other CELF proteins including CELF2 (ETR-3) and CELF4 have been shown to increase as mouse thigh and heart muscle develop [23,27]. These CELF proteins also regulate alternative splicing of sarcomere gene exons [27,129–131], for example CELF4 promotes splicing of mature isoforms of chicken β-tropomyosin [132]. CELF2 expression in chicken and mouse shifts from preferential production of an embryonic 52 kDa isoform to lower 42 and 50 kDa adult isoforms that correlates with changes in cTNT splicing [23]. Taken together, our results with Bru1 in *Drosophila* reflect a general requirement for CELF activity throughout myogenesis to regulate developmental transitions and fiber-type-specific isoform expression.

Our data suggest that IFM are sensitive to both the timing and dosage of Bru1 expression. Early overexpression of Bru1 with Him-Gal4 resulted in IFM with a hole in the center of the myofiber, and rescue with Him-Gal4 enhanced myofiber, myofibril, sarcomere, and contractile defects, indicating that too much Bru1 during early stages of myogenesis is detrimental to development (Fig 7). Early overexpression of CELF1 in mice with a β-actin or MCK promoter leads to central nuclei, reduced muscle mass, increased expression of p21, Myogenin and Mef2A, and lethality where phenotypic severity is directly correlated with the level of CELF1 overexpression [29,133]. In cell culture, overexpression of CELF1 in myocytes promotes the cell cycle and inhibits differentiation [134]. Our data are consistent with and extend these results to show how overexpression of Bru1 affects myofibril assembly. Interestingly, recent work demonstrates that steady-state protein expression levels of CELF1 are significantly affected by use of alternative 3′-UTR regions [135] and that increased nuclear activity of CELF1 affecting alternative splicing but not increased cytoplasmic activity affecting translation in adult muscle leads to severe histopathology [136], revealing that muscles possess multiple mechanisms to finely-tune CELF expression levels. One mechanism that might explain Bru1 dosage effects is differential sensitivity of Bru1 targets. It has been shown that splicing of CELF1 targets Bin1 and Mef2A are only disrupted in a strong MHC-CELFΔ line and not in a milder line, indicating that different targets have different thresholds of responsiveness to CELF activity [116]. Alternatively, dosage sensitivity may reflect interactions with other co-regulatory RBPs. RBPs are suggested to function in multi-factor complexes, such as the RBFOX1 containing LASR complex [137], where different combinations of constituent proteins lead to different regulatory outcomes. CELF proteins have been reported to have antagonistic interactions with both MBNL [21,24] and RBFOX family proteins [27,43]. Around 22% to 30% of splice events are co-regulated by RBFOX2 and either CELF1 or CELF2 in mammalian heart, and RBFOX family motifs are enriched downstream of exons regulated after *bru1* RNAi in flies [27], suggesting functional conservation of antagonism. Further studies are needed to determine the molecular mechanisms underlying CELF family dosage sensitivity and to elucidate the co-regulatory logic of RBPs that participate in the fiber-type-specific RNA-regulatory network.

Up-regulation of CELF1 activity in differentiated muscle is thought to play a major role in the pathogenesis of myotonic dystrophy Type I (DM1) (OMIM 160900). CTG repeat expansions in the DMPK gene sequester and functionally deplete MBNL, leading to increased nuclear localization and activity of CELF1, with longer CTG repeats correlated with increased disease severity and decreased age of onset [138,139]. CELF1 is normally down-regulated 5- to 10-fold in adult vertebrate muscle [21,23,115] and is reported to be required during early muscle development [116,128,140], such that increased nuclear activity in mature muscle results in a reversion to embryonic splicing patterns [21,25,28,30,136]. In *Drosophila*, Bru1 levels also decrease as the IFM develop [43], and our data show that overexpression of Bru1 with late-stage Fln-Gal4 impairs muscle function and sarcomere structure (Fig 8). When we rescued the *bru1*^*M3*^ mutant with Fln-Gal4 driven UAS-Bru1, we were able to largely restore mature patterns of alternative splicing and partially abrogate hypercontraction and myofiber loss, but we could not rescue myofibril and sarcomere structural defects (Fig 8). Given the parallels between Bru1 and CELF1 function in flies and vertebrates, our results provide mechanistic insight into the variable efficacy and phenotypes observed during the development of DM1 therapeutics [141,142]. Current nucleic acid therapeutics or genome modification approaches are focused on restoring MBNL function by increasing MBNL expression levels, blocking MBNL binding to CTG-repeats or editing the DMPK locus [143,144], in turn decreasing CELF1 activity. Consistent with our rescue data in flies, clinical trials and nonclinical studies in cells and mice models often are able to restore mature patterns of alternative splicing and improve symptoms of myotonia [142,144,145], providing a positive outlook for the development of therapeutics that can significantly improve patient quality of life. However, our results indicate that a cure for DM1 will need to restore the balance in MBNL and CELF regulation to avoid dosage artifacts, and that timely intervention and early administration of therapeutics will be necessary to arrest the developmental progression that results in sarcomere structural defects and contractile deficits. Taken together, our results advance understanding of Bru1 function and more broadly highlight the importance of CELF protein dosage and the progressive nature of CELF phenotypes over time.

## Materials and methods

### Fly stocks and crosses

Experimental work with *Drosophila melanogaster* was approved under German §15 GenTSV (license number 55.1-8791-14.1099). Fly stocks were maintained using standard culture conditions at room temperature. Fly food was made by combining 16 L water, 1,300 g corn flour, 150 g soy flour, 1,300 g molasses, 130 g agar, 300 g yeast, and 650 g malt extract in a water-jacketed cooker. After food was sufficiently cool, 415 ml of 10% Nipagin and 295 ml acid mix (3% phosphoric acid, 21% propionic acid) were added. Food was distributed in vials and bottles with a peristaltic pump, dried at room temperature and stored at 4°C till use. All experimental crosses were kept in a 27°C incubator.

*w*^*1118*^ alone or in combination with the relevant Gal4 driver (Gal4 x *w*^*1118*^) was used as the wild-type control. We additionally validated the Gal4 x *w*^*1118*^ control by verifying that flight ability, fiber integrity, and sarcomere length and width were not altered in the Gal4 driver crossed to *w*^*1118*^ as compared to the Gal4 driver crossed to UAS-GFP (gift from Ulrike Gaul) or UAS-GFP-RNAi (Bloomington 41551) (S7 Fig). The *bru1*^*M2*^ and *bru1*^*M3*^ alleles were generated by insertion of a selectable 3xP3-DsRed cassette using a CRISPR-Cas9 approach [146]. The *bru1*^*M2*^ mutant contains a cassette insertion upstream of *bru1* exon 12, and has been described previously [43]. *bru1*^*M3*^ is described in this manuscript. The UAS-Bru1 line was generated by amplifying the full-length *bru1-RA* transcript from *w*^*1118*^ using RT-PCR (primer sequences are available in S4 Table), cloning the cDNA into the *pUAST-attB* transformation vector containing a 5× UAS-hsp70 promoter region and a SV40 polyadenylation sequence, and integrating at the attP-86Fb landing site [147] with ϕC31 integrase (S8A Fig). RNAi against *bru1* (*bru1-IR*) was achieved with a previously characterized hairpin (GD41568) [14,43] obtained from the Vienna Drosophila Resource Center (VDRC). Df(2L)BSC407 is a deficiency allele on chromosome 2L that includes the *bru1* locus and was obtained from the Bloomington Drosophila Stock Center (BDSC). Mhc^10^ is a TDT and IFM-specific amorphic myosin mutant [148]. Gal4 driver lines used in this study include: *Mef2*-Gal4 [149], which constitutively drives in all muscles; *Him*-Gal4 [42], which drives strongly in myoblasts and at early stages of IFM development until about 30 h APF; *salm*-Gal4 [84], which expresses in IFM starting from approximately 16 h APF; *Act88F*-Gal4 [77], which drives strongly in IFM starting at about 24 h APF; *UH3*-Gal4 [80], which expresses specifically in IFM starting at around 36 h APF; and *Fln*-Gal4 [83], which is expressed IFM-specifically from about 56 h APF (S6A Fig). weeP26 is a GFP-trap line inserted in the intron between *Mhc* exon 36 and 37, which only tags the long isoform of *Mhc* that terminates in exon 37 after the insertion [44,66,150]. Act88F localization was tracked using UASp-GFP-Act88F [75]. GFP-tagged fosmid reporter fly lines included Strn-Mlck-GFP (strn^4^, which tags IFM-specific isoform R) [14], wupA-GFP (fTRG925), Kettin-GFP (fTRG477), Clip190-GFP (fTRG156), Mlp84B-GFP (fTRG678), and Mlp60A-GFP (fTRG709) [151]. Talin-YPet is a CRISPR-mediated endogenous tag that strongly localizes to tendon attachment sites at muscle tips [152]. All fly lines and reagents are listed in the Resources S5 Table.

### Generation of the *bru1*^*M3*^ CRISPR allele

*bru1*^*M3*^ was generated by CRISPR-mediated genome modification, using a previously described approach [146]. Two sgRNAs targeting sequences in the intron between *bru1* exon 17 and 18 and after the 3′-UTR downstream of *bru1* (S1A Fig and S4 Table) were screened in S2 cells for cutting efficacy and then co-injected with a 3x-P3 DsRed integration cassette flanked by approximately 1 kilobase long arms homologous to the genome sequence immediately upstream and downstream, respectively, of the sgRNA cut sites. The integration cassette contains a strong splice-acceptor followed by a 3 frame stop and a poly-adenylation sequence. Instead of the intended deletion, our genome modification resulted in the insertion of the cassette into the intron just upstream of exon 18, the last coding exon shared by all *bru1* isoforms (S1A and S1B Fig). Although we could detect increased expression of *bru1* mRNAs that include upstream exons 12 to 14, splicing into exon 18 was dramatically reduced, while splicing from exon 17 into the inserted construct containing the SV40 polyadenylation sequence was strongly increased (S1C Fig). Splicing into the integrated cassette in *bru1*^*M3*^ results in deletion of exon 18, which encodes the terminal 88 amino acids (aa516-604 of *bru1-RA*) in the third RRM domain (the extended RRM3 domain comprises aa471-604 [39]). *bru1*^*M3*^ is therefore a truncation allele that results in early termination after exon 17, which affects all *bru1* isoforms (S1D Fig).

### Behavioral assays

Flight ability was assayed as described previously [153]. Briefly, *N* > 30 adult male flies were collected under CO_2_ on day 1 after eclosion, recovered overnight at 27°C and introduced into a 1-meter-long cylinder divided into 5 zones. Flies with a “normal flight” ability landed in the top 2 zones, “weak fliers” in the middle two, and “flightless” males fell to the bottom. Eclosion competence was assayed by counting the number of adult flies that emerged from their pupal cases. At least 60 live pupae were unbiasedly selected after 48 h APF and monitored until eclosion.

### Immunofluorescence staining

Pupae of the desired genotype were tightly staged as follows: newly pupated 0 h APF flies (motionless, transparent-white body color and everted spiracles) were selected and sorted by sex, and males were transferred to a wetted filter paper in a 60 mm Petri dish and maintained at 27°C until the desired time point. Pupae and adult flies were dissected and stained as described previously [154]. For early pupae (before 48 h APF), we performed open-book dissections by removing the ventral half of the pupa to expose the developing IFM. For late pupal (after 48 h APF) to adult fly stages, we bisected the thorax sagittally after fixation to allow visualization of IFM. All samples were fixed in 4% PFA in 0.5% PBS-T (1× PBS + Triton X-100) for 30 to 60 min. For primary antibody staining, samples were blocked in 5% normal goat serum in 0.5% PBS-T for 90 min at room temperature, and then incubated overnight at 4°C with rabbit anti-Bru1 (1:500) [43] or rabbit anti-GFP (1:1,000, Abcam ab290). All antibodies are listed in the Resources S5 Table. After washing 3 times in 0.5% PBS-T for 10 min at room temperature, samples were incubated for 2 h at room temperature with secondary antibody Alexa 488 goat anti-rabbit IgG or rhodamine-phalloidin (1:500, Invitrogen, Molecular Probes). Samples were washed 3 times in 0.5% PBS-T and mounted in Vectashield containing DAPI.

### Cryosectioning and histological staining

Pupae were staged as above. Cryosections were performed as described previously [42]. Samples were removed from the pupal case and fixed in 4% PFA in 0.5% PBS-T overnight at 4°C, and then incubated overnight in 30% sucrose in 0.5% PBS-T at 4°C on a rocking shaker. Pupae or 1 d adult thoraxes were arranged ventral side down in a vinyl specimen mold (Sakura Finteck), embedded in Tissue-Tek O.C.T. (Sakura Finteck) and snap-frozen on dry ice. Blocks were stored at −80°C, and then sectioned from anterior to posterior at 30 μm on a cryostat (Leica). Sections were collected on glass slides coated with 0.44 mM chromium potassium sulfate dodecahydrate in 1% gelatin to avoid detachment of the tissue during subsequent washing steps. Slides were post-fixed for 5 min in 4% PFA in 0.5% PBS-T at room temperature, washed 2 times in 0.5% PBS-T, and stained with rhodamine-phalloidin (1:500) for 2 h at room temperature in a humidity chamber protected from light. Slides were washed 3 times in 0.5% PBS-T, mounted with Fluoroshield containing DAPI (Sigma), and imaged on a Leica SP8X WLL upright confocal.

For hematoxylin-eosin staining, slides were post-fixed in formalin for 10 min, washed in 35°C running tap water, and rinsed in distilled water. Slides were incubated with Harris Hematoxylin stain (Roth) for 1 min, rinsed with distilled water, and placed under running tap water for 7 min to remove excess stain. After rinsing with distilled water, slides were stained in 1% Eosin solution (Apotheke Klinikum der Universität München) for 2 min, rinsed in distilled water, and dehydrated in 70% EtOH, 96% EtOH, and 99% EtOH in consecutive 5-min incubation steps. Slides were placed in xylenes (Roth) and mounted with Eukitt (Orsatec). After drying, slides were imaged on an Olympus IX83 inverted microscope with a 0.95 NA 40× objective.

### Confocal microscopy

Laser-scanning confocal images were acquired on a Leica SP8X WLL upright confocal using Leica LAS X software in the Core Facility Bioimaging at the LMU, Biomedical Center (Martinsried, DE). Sagittal hemi-thorax sections were imaged with an HCPL FLUOTAR 10×/0.30 objective to resolve the whole fiber morphology and with an HCPL APO 63×/1.4 OIL CS2 objective to capture myofibril and sarcomere structure. Fibers of early pupae (before 48 h APF) were imaged with an HCPL APO 20×/0.75 IMM CORR CS2 objective. Samples stained with rabbit anti-Bru1 were processed in replicates using same antibody mix and were imaged with the same laser gain settings. 3D stereo projection movies of GFP-Act88F and Mhc^Wee-P26^-GFP myofibril incorporation were assembled with Leica LAS X software from 0.1 mm confocal Z-stacks. Samples for the live imaging of spontaneous flight muscle contractions at 48 h and 72 h APF were prepared as previously described [155], and 10-min live recordings were obtained at a frame rate of 0.65 s/frame with an HCPL APO 40×/1.30 WATER CS2 objective. Samples for developmental GFP-tagged fosmid reporter expression at 48 h, 72 h, and 1 d adult were stained with the same antibody mix and images acquired with the same laser settings on a Zeiss LSM 780 confocal microscope using a Plan-APOCHROMAT 100×/1.46 oil immersion objective lens.

### Transmission electron microscopy

*w*^*1118*^ or *bru1*^*M3*^ pupae were staged as described above to 48 h, 60 h, 72 h, or 90 h APF, and removed from the pupal case. Approximately 70% of the abdomen was carefully removed in 0.1 M cacodylate buffer using fine scissors, and thoraxes were incubated in glutaraldehyde fix solution (4% glutaraldehyde in 0.1 M cacodylate solution with 3% sucrose) for 2 h at room temperature on a rocking shaker. After pre-fixation, pupae were cut sagittally with a #C35 Feather microtome blade (Feather), and then fixed overnight in glutaraldehyde fix solution on a rocker at 4°C. Samples were post-fixed in osmium tetroxide (1% in cacodylate buffer) and stained with uranyl acetate (1% in water), dehydrated in a graded acetone series, and embedded in spurr low-viscosity epoxy resin following standard protocols. Samples were sectioned at 1 μm on an ultramicrotome (RMC MT 7000) with a diamond knife as previously described [156]. Sections were collected on glass slides, stained with Richardson blue and evaluated on an Olympus BX61VS light microscope until the sectioning depth of the IFM was reached. Then, the ultrathin sections were made at 60 to 70 nm, collected on TEM grids, and post-stained with uranyl acetate (1% in water) and lead citrate to enhance contrast. Images were acquired on an FEI Morgagni transmission electron microscope at 80 kV with a side-mounted SIS Megaview 1K CCD camera. At least 2 individual samples per genotype and time point were analyzed.

### RNA isolation, RT-PCR, and RT-qPCR

All primers are listed in S4 Table. Whole thorax samples were prepared from 10 or more flies by removing the head, wings, and abdomen in a drop of pre-cooled 1× PBS using a fine scissors. Dissected IFM (>30 flies) and TDT (>60 flies) muscle samples were prepared from 1 d adult flies as described previously [44]. Legs were removed from >15 flies using fine scissors. Samples were snap-frozen in 50 μl of TRIzol (TRIzol Reagent; Ambion) on dry ice and stored at −80°C. Total RNA was isolated using TRIzol-Chloroform per the manufacturer’s guidelines. RNA samples were treated with DNaseI (New England Biolabs) and concentration was assessed using a Qubit 2.0 Fluorometer (Invitrogen). For normalization purposes, equivalent quantities of total RNA were used for cDNA synthesis with the LunaScript RT SuperMix Kit (New England Biolabs). For RT-PCR, 1 μg of cDNA was amplified with Phusion polymerase for 32 cycles and separated on a standard 1% agarose gel together with a 100 bp or 1 kb ladder (New England Biolabs). Ribosomal protein L32 (RpL32, RP49) served as an internal control for some reactions. Semi-quantitative analysis of gel band intensity to determine differences in exon use was performed using the “gel analysis” feature in Fiji. Band intensity was normalized against RpL32 to compare expression levels, or was used to calculate percent exon usage as: 100× (individual band intensity)/Σ (intensity of all bands observed for same primer pair).

For RT-qPCR, total RNA was extracted from IFM dissected from 150 flies using TRIzol. Reverse transcription and cDNA generation were performed with the LunaScript RT SuperMix kit, starting with 1 μg of cDNA. cDNA was diluted from 1:8 to 1:20, depending experimentally on the expression level of the target. Samples were assayed using SYBRgreen on a QuantStudio 3 (Applied Biosystems by Thermo Fisher Scientific) with an extension temperature of 60°C for 40 cycles. Samples were normalized against either RpL32 or Vha44, and fold change was calculated using the 2^-ΔΔCT^ method [157]. Data were plotted in GraphPad Prism, and significance was calculated from at least 3 replicates using either a Student’s *t* test (for a single sample versus control) or ANOVA (for more than 2 samples).

### Image analysis

Image analysis was performed in Image J/Fiji [158]. For every confocal-based assay, 10 to 15 images were acquired from >10 adult flies or pupae. For TEM, more than 20 images were acquired from 2 to 3 animals. DLM fiber integrity and length were measured from Z-stack projections of hemi-thorax samples based on rhodamine-phalloidin staining. Fiber length was measured as distance (μm) from anterior to posterior tip of a single fiber using the freehand drawing tool in Fiji. The same tool was used on TEM images, after setting the scale based on the inset in each image, to measure the sarcomere length and myofibril width as the distance between or across Z-disks, respectively. For confocal images, sarcomere length and myofibril width of sagittal sections, as well as myofibril diameter and density of cross-sections, were measured based on phalloidin (F-actin) staining using MyofibrilJ [42] (https://imagej.net/MyofibrilJ). The number of myofibrils per bundle was quantified in a semi-automated manner as follows: the boundaries of individual fiber bundles were determined from the Z-stack and at least 3 separate and complete bundles from >10 flies were cropped, analyzed with MyofibrilJ, and then manually corrected for accuracy (myofibril “dots” were added or subtracted based on manual inspection of the MyofibrilJ output). Each “hook” or “ring” structured myofibril was counted as one. The number of myofibrils classified as “dots,” “hooks,” or “rings” were manually counted per defined cross-sectional area. The number of actin cables per fiber (at 26 h and 28 h APF) was determined manually based on rhodamine-phalloidin staining. Bru1 signal intensity was analyzed as follows: the nuclei were selected based on DAPI staining using the wand tool in Fiji, and relative Bru1 signal in the nuclei (overlapping the DAPI positive regions) was quantified as the mean intensity of Bru1 staining divided by the mean intensity of the absolute background.

Quantification of spontaneous contractions in developing DLM has been described previously [42] and was modified here as follows. Live movies were obtained for 10 min, and “twitches” in each visible DLM fiber at 48 h APF were classified as single, double, or triple contraction events, and resulted in displacement of the myofiber and return to the original resting position. The range of each single spontaneous contraction at 48 h APF was measured using the freehand drawing tool in Fiji as the distance between the tip of the fiber prior to and after the extension. At 72 h APF, contraction events quantified in the *bru1* mutant were slower than “twitches” and resulted in a one-directional myofiber extension, i.e., without return to the original resting position. All data were tabulated in Excel, and plotting and statistical analysis were performed in GraphPad Prism 8.4.0, using ANOVA or unpaired Student’s *t* test.

### Deconvolution

Deconvolution was performed in ImageJ using the Diffraction PSF 3D and FFTJ–DeconvolutionJ plugins. Images were obtained at a resolution of 23.2848 pixels per micron and a corresponding voxel size of 0.0429 × 0.0429 × 0.2014 micron^3^ for deconvolution and 3D projection. YZ stacks were generated with interpolation using the Reslice option native to ImageJ. A digital point spread function (PSF) was generated with the following settings: IR = 1.518, NA = 1.40, 580 nm, pixel spacing 23.28 and corresponding slice spacing of 4.97 units, with a Rayleigh resolution of 10.68 pixels. Deconvolution was performed without resizing at 32-bit, double precision, and with a gamma setting of 0.01/0.03.

### Proteomics

IFM were dissected as described previously [44], and 30 flies per genotype were dissected from *w*^*1118*^ and *bru1*^*M2*^ at 72 h APF and 1 d adult, and 4 biological replicates were prepared per genotype. Samples were processed according to the manufacturer’s instructions using the PreOmics iST Sample Preparation Kit (Preomics, #0000.0061) and analyzed by the Protein Analysis Unit (ZfP) at the LMU Biomedical Center as follows. Desalted peptides were injected in an Ultimate 3000 RSLCnano system (Thermo) and separated in a 25-cm analytical column (75 μm ID, 1.6 μm C18, IonOpticks) with a 100-min gradient from 5% to 60% acetonitrile in 0.1% formic acid. The effluent from the HPLC was directly electrosprayed into a Qexactive HF (Thermo) operated in data-dependent mode to automatically switch between full scan MS and MS/MS acquisition. Survey full scan MS spectra (from m/z 375–1,600) were acquired with resolution R = 60,000 at m/z 400 (AGC target of 3 × 10^6^). The 10 most intense peptide ions with charge states between 2 and 5 were sequentially isolated to a target value of 1 × 10^5^, and fragmented at 27% normalized collision energy. Typical mass spectrometric conditions were: spray voltage, 1.5 kV; no sheath and auxiliary gas flow; heated capillary temperature, 250°C; ion selection threshold, 33,000 counts. MaxQuant 1.6.14.0 [159] was used to identify proteins and quantify by LFQ with the following parameters: Database, Uniprot_AUP000000803_Dmelanogaster_Isoforms_20210325.fasta; MS tol, 10 ppm; MS/MS tol, 20 ppm; Peptide FDR, 0.1; Protein FDR, 0.01 Min. peptide Length, 5; Variable modifications, Oxidation (M); Fixed modifications, Carbamidomethyl (C); Peptides for protein quantitation, razor and unique; Min. peptides, 1; Min. ratio count, 2. Additional analysis was performed in Perseus [160]. We filtered the data to retain biologically relevant protein groups with missing intensities between mutant and control samples (MNAR values) by requiring at least 3 replicates in either control or mutant to contain a value, and we imputed missing values by replacement with a constant value (lowest observed intensity– 1). Differential expression was tested by *t* test with FDR = 0.05. Results were exported and further visualization performed in R.

### mRNA-Seq and transcriptome bioinformatic analysis

For transcriptome analysis, IFM were dissected from 1 d adult (0 to 24 h after eclosion) *w*^*1118*^ and *bru1*^*M3*^ flies as described previously [44]. Two replicates of IFM from 100 flies were dissected per genotype. RNA was isolated using TRIzol and sent to LC Sciences (Houston, TX) for sequencing. After quality verification, poly-A mRNA selection and library construction, samples were sequenced as stranded, 150 bp paired-end on an Illumina HiSeq to a depth greater than 70 million reads. Transcriptome data from *bru1-IR* and control IFM at 24 h, 30 h, 72 h, and 1 d adult was generated previously [14,42], and was reanalyzed as part of this manuscript.

Sequence data was mapped with STAR to ENSEMBL genome assembly BDGP6.22 (annotation dmel_r6.32 (FB2020_01)). Files were indexed with SAMtools and processed through featureCounts. Downstream analysis and visualization were performed in R using packages listed in the S5 Table. Differential expression was analyzed at the gene level with DESeq2 and at the exon level with DEXSeq. Differential exon use can reflect alternative splicing as well as alternative promoter use. Both packages were additionally used to generate normalized counts values. We employed previously annotated sets of genes, including sarcomere proteins [42], genes with an RNAi phenotype in muscle [153], core fibrillar genes regulated by Spalt major and differentially expressed between IFM and tubular muscle [14], mitochondrial proteins [104,161], and all genes with the Flybase GO term “muscle contraction,” “actin cytoskeleton,” or “actin cytoskeleton organization.” Genes with a hypercontraction phenotype in *Drosophila* muscle were curated by hand from the literature. Full lists of all gene categories are available in S2 Table. Plots were generated using ggplot2 or ComplexHeatmap. Unless otherwise stated, we used an adjusted *p*-value threshold of 0.05 for significance. If a log_2_FoldChange threshold was used in a specific analysis, it is specified in the legend for the relevant figure panel.

## Supporting information

S1 Fig Molecular and phenotypic verification of the *bru1*^*M3*^ CRISPR allele. **(A)** Diagram of the C-terminal region of the *bruno1* (*bru1*) locus and mRNA isoforms RA, RB, and RD (exons, purple; UTRs, yellow). Location of the RNA recognition motif domains (RRM, light red), target region of anti-Bru1 antibody (brown), target region of *bru1-IR* GD41568 hairpin (brown), location of *bru1*^*M2*^ construct insertion site (brown), and the sgRNAs (blue) used for CRISPR-mediated generation of the *bru1*^*M3*^ hypomorph allele are marked. Transgenic construct is inserted upstream of exon 18 and contains a strong splice acceptor (SA, light blue), a triple frame stop (stop, red), an SV40 polyadenylation signal (orange) and a selectable 3xP3-dsRed marker (crimson) flanked by homology arms (light tan). Exon numbering according to the annotation FB2021-05. **(B)** Whole-fly genomic PCR verifying dsRed cassette insertion in the *bru1* locus. Identity of amplified region marked on the left, band size noted on the right. Primer sequences available in S4 Table. **(C)** RT-PCR to test expression of *bru1* mRNA in whole-thorax and dissected IFM. Identity of amplified region marked on the left, band size noted on the right. RpL32 used as internal control. **(D)** Diagram of the *bru1*^*M3*^ allele. Splicing from exon 17 is redirected into the splice acceptor (SA) of the inserted construct (red line, A), leading to early termination of the *bru1* mRNA and truncation of RRM3. Splicing from exon 17 to exon 18 is strongly reduced (dotted red line, B), and signal from 3′-UTR exon 21 is not detectable. **(E, F)** Confocal projections of 1d adult hemithoraxes showing IFM from *bru1*^*M3*^/+ and *bru1*^*M3*^/Df(2L)BSC407. Deficiency BSC407 covers the complete *bru1* locus. Thorax boundaries in (F), dashed line; phalloidin stained actin, gray; scale bar = 100 μm. **(E’–F’)** Single-plane confocal images of 1 d adult IFM myofibrils. Scale bar = 5 μm. **(G, H)** Quantification of sarcomere length (G) and myofibril width (H) from TEM data shown in Fig 1F. Boxplots are shown with Tukey whiskers, outlier data points marked as black dots. Significance determined by ANOVA and post hoc Tukey (ns, not significant; ***, p-val < 0.001). **(I, J)** Quantification of Z-disc alignment (I) and sarcomere morphology (J) defects from TEM data shown in Fig 1F. *N* > 20 images from 2 biological samples for each individual genotype and time point. **(K)** Dot plot showing the correlation between all detected peptide groups and their corresponding mRNA expression level in *bru1*^*-/-*^ versus w^1118^ IFM (proteins with a significantly DE exon, orange; significantly DE genes, purple). The Pearson’s/Spearman’s correlation coefficients (top left corner) and regression line (blue) indicate a weak but positive correlation. Underlying data can be found in S1 and S1 Fig Source Data and Gels as listed in S6 Table.
(TIFF)

S2 Fig Fiber-type specific alternative splicing of muscle proteins is disrupted in *bru1*^*-/-*^ IFM. **(A)** Boxplot of gene (DESeq2), exon (DEXSeq) and protein level (mass spec) expression changes between 1 d adult *bru1*^*-/-*^ and *w*^*1118*^ IFM in select categories of genes including GO term “actin cytoskeleton organization,” microtubule associated genes, mitochondrial genes, GO term “muscle contraction,” RNA-binding proteins (RBPs), sarcomere proteins (SPs), and fibrillar core genes. Blue dot denotes *p* ≤ 0.05. **(B)** Venn diagram of the overlap between all significantly DE genes (purple), exons (orange), and proteins (green) between *bru1*^*-/-*^ versus *w*^*1118*^ IFM in 1 d adults. **(C–E)** Semi-quantitative RT-PCR verification of alternative splice events in *Strn-Mlck* (C), *wupA* (D), and *Mhc* (E). Top: scheme of alternative isoforms with primer locations. Exon numbering in accordance with the FB2021_05 annotation. Color coding of depicted isoforms consistent with bottom panel; 3′-UTR regions in light beige. Middle: Quantification of relative expression level of splice events in tubular leg and jump (tergal depressor of the trochanter, TDT) and fibrillar IFM in control flies and in *bru1*^*M3*^ IFM. Error bars = SD. Bottom: representative RT-PCR gel image. **(F–N’)** Misexpression of GFP-tagged sarcomere proteins in *bru1*^*M3*^ IFM. (**F, I, L)** Diagrams of reporter GFP incorporation into tagged transcripts of *Strn-Mlck* (F), *wupA* (I), and *Mhc*-weeP26-GFP (L). Exons, magenta; 3′-UTR, tan; SA, splice acceptor; SD, splice donor; sGFP, superfold GFP. **(G–N)** Intensity matched single-plane confocal images from control and *bru1*^*M3*^ IFM at 90 h APF showing incorporation of Strn-Mlck-IsoR-GFP (G, H), wupA-GFP (J, K), and Mhc-weeP26-GFP (M, N). Strn-Mlck isoform R with sGFP tagged exon 25 is strongly expressed in wild-type IFM (G’) but absent from *bru1*^*M3*^ (H’). WupA with an sGFP tagged exon 3 is normally absent from wild-type IFM (J’) but gained in *bru1*^*M3*^ (K’). Expression of the Mhc isoform containing exon 37 and tagged in weeP26-GFP is normally restricted to early IFM development (M–M’), but is altered in *bru1*^*M3*^ (N–N’). GFP-tag, green; phalloidin stained actin, magenta (G, H, J, K, M, N); pseudo-coloring, GFP-intensity (compare G’ and H’; J’ and K’; M’ and N’). Scale bar = 5 μm. **(O–Q)** Semi-quantitative RT-PCR verification of alternative splice events in *Zasp52* (O), *Tm1* (P), and *sls* (Q). Top: schemes of alternative isoforms. Bottom: representative RT-PCR gel image. Labeled as in (C–E). Underlying data can be found in S2 Table and S2 Fig Source Data and Gels, and the RNA-Seq data tables as listed in S6 Table.
(TIFF)

S3 Fig Incorporation of both Act88F and Mhc into growing myofibrils is abnormal in *bru1*^*-/-*^ IFM. **(A)** mRNA expression level raw C_T_ values assayed by RT-qPCR for different genes in *bru1*^*M3*^ (red dots) and *w*^*1118*^ (white dots) IFM, including *Act88F*, *Vps35*, *Lamp1*, *Vha44*, *mical*, *Nedd8*, *Atg2*, *Ald1*, and *His3*.*3B* (2 independent primer sets: N1 and N2, were used). Some genes, such as *His3*.*3B* and *Ald1* are changed in *bru1*^*M3*^, and not suited as a normalization standard. **(B–C”‘)** Single plane confocal images of *Fln*-Gal4 driven pUAS-GFP-Actin88F incorporation into control (B–B’”) and *bru1*^*M3*^ (C–C’”) at 90 h APF. Longitudinal sections (B, C) represent the XY-axis, while vertical lines mark the exact position of orthogonal slices at the z-disc (yellow line) and M-line (cyan line). Orthogonal view (B’–B’”, C’–C’”) represents YZ-axis of (B, C), respectively. GFP, green; phalloidin stained actin, magenta; scale bar = 5 μm. **(D–G”‘)** Single-plane confocal images of Mhc-weeP26-GFP expression in control (D–E””) and *bru1*^*M3*^ (F–G””) at 90 h APF. Mhc-weeP26-GFP labels a specific isoform of Mhc that is only expressed during early IFM development. Longitudinal (D, F) and orthogonal sections (E–E””, G–G””) are shown as above at the z-disc (yellow line) and M-line (blue lines). GFP, green; phalloidin stained actin, magenta; scale bar = 5 μm. **(H)** Violin plots of changes in expression of tubular-preferential genes and exons. Left plot shows changes in tubular-preferential gene and exon expression in tubular leg versus fibrillar wild-type IFM (yellow) and *bru1*^*M3*^ versus wild-type IFM (red). Tubular-preferential was defined as all genes or exons with a log_2_FC > 1 and an adjusted *p*-value < 0.05 in the leg versus IFM comparison. Some but not the majority of tubular genes and exons are up-regulated in *bru1*^*M3*^ IFM. Right plot shows how the same tubular-preferential gene/exon sets change expression with time in IFM when comparing 1 d adult IFM to 24 h APF IFM in control (gray) or *bru1-IR* (orange) IFM. **(I)** Violin plots of changes in expression of fibrillar-preferential genes and exons. Left plot shows changes in fibrillar-preferential gene and exon expression in tubular leg versus fibrillar wild-type IFM (yellow) and *bru1*^*M3*^ versus wild-type IFM (red). Fibrillar-preferential was defined as all genes or exons with a log_2_FC < -1 and an adjusted *p*-value <0.05 in the leg versus IFM comparison, and thus expressed higher in IFM than in leg. Right plot shows how the same fibrillar-preferential gene/exon sets change expression with time in IFM when comparing 1 d adult IFM to 24 h APF IFM in control (gray) or *bru1-IR* (orange) IFM. Underlying data can be found in S3 Fig Source Data and the RNA-Seq data tables as listed in S6 Table.
(TIFF)

S4 Fig Temporal dynamics of gene expression and exon use in *bru1-IR* IFM across muscle development. **(A)** Top: Boxplot of changes in gene expression across the *bru1-IR* time course (*bru1-IR* versus control) for temporal-switch genes that are normally up-regulated (log_2_FC > 0, p-adj ≤ 0.05) or down-regulated (log_2_FC < 0, p-adj ≤ 0.05) in control IFM from 24 h APF to 1 d adult. Bottom: Boxplot of changes in exon use across the *bru1-IR* time course for temporal-switch exons that are normally up-regulated (log_2_FC > 0, p-value ≤ 0.05) or down-regulated (log_2_FC < 0, *p*-value ≤ 0.05) in control IFM from 24 h APF to 1 d adult. Blue dot denotes *p* ≤ 0.05. **(B)** Boxplot of changes in gene expression (DESeq2) and exon use (DEXSeq) in GO term “actin cytoskeleton” genes in *bru1-IR* versus control IFM at 24 h, 30 h, 72 h APF, and in 1 d adult. Blue dot denotes *p* ≤ 0.05. **(C)** Heatmap of gene level-expression changes in all sarcomere protein and fibrillar muscle genes at all time points in *bru1-IR* versus control IFM. The fifth column shows the temporal change in use of the same genes in wild-type IFM from 24 h APF to 1 d adult. **(D)** Heatmap of all exons significantly DE (DEXSeq, p-val ≤ 0.05) at any time point in *bru1-IR* versus control IFM. The fifth column shows the temporal change in use of the same exons in wild-type IFM from 24 h APF to 1 d adult. Underlying data can be found in S2 Table, and the RNA-Seq data tables as listed in S6 Table.
(TIFF)

S5 Fig GFP-tagged sarcomere protein reporters reveal temporal misexpression dynamics in *bru1-IR* IFM. **(A)** Plot of mRNA-Seq based gene-level expression of *wupA* (maroon), *sls* (blue), *CLIP-190* (green), *Mlp60A* (purple) and *Mlp84B* (orange) in *bru1-IR* (dark colors) and control (light colors) IFM at 24 h, 30 h, and 72 h APF and in 1 d adult. log_2_(counts) of DESeq2 count values normalized across all 4 time points are plotted. Gene expression levels of *wupA*, *Kettin*, and *Clip190* are consistent between control and *bru1-IR* across the mRNA time course. Both Mlp60A and Mlp84B encode a single protein isoform that shows strong up-regulation at the gene level in *bru1-IR* IFM from 72 h APF to 1 d adult. **(B)** Plot of mRNA-Seq based exon-level expression of *wupA exon 1* (maroon), *sls exon 38* (blue), *CLIP-190 exon 29* (green), *Mlp60A exon 13* (purple), and *Mlp84B exon 2* (orange) in *bru1-IR* (dark colors) and control (light colors) IFM at 24 h, 30 h, and 72 h APF and in 1 d adult. There are differences in the use of the isoform containing the exon where the GFP tag is inserted in *wupA*, *Kettin*, and *Clip190*. log_2_(counts) of DEXSeq normalized count values are plotted. The selected exons contain the GFP-tag visualized in (C–G). **(C–E)** Expression of select splice-isoforms of *wupA* (C), *sls* (D), and *CLIP-190* (E) visualized by GFP-tag fluorescence (grayscale) in intensity-matched, single-plane confocal micrographs of IFM from control (left) and *bru1-IR* (right) flies at 48 h and 72 h APF and 1 d adult. *wupA-GFP*, which labels a termination used preferentially in tubular muscle [14] (see also S2D, S2I, S2J’, and S2K’), is already visible in *bru1-IR* but not control IFM at 48 h and 72 h APF (C), confirming its missplicing throughout development in *bru1-IR* IFM. Kettin encodes a short isoform of *sls* that is preferentially expressed in tubular muscle [162]. The GFP-tag in *sls* labels the Kettin isoform. Kettin-GFP is not expressed in control IFM, but is observed in *bru1-IR* IFM from 72 h APF (D). A GFP tag inserted in exon 29 of *Clip-190* is only expressed weakly in adult IFM in control, but is already expressed at 48 h and strongly at 72 h APF in *bru1-IR* IFM (E). GFP, green; phalloidin stained actin, magenta; scale bar = 5 μm. **(F, G)** Single-plane confocal images of GFP-tagged Mlp60A and Mlp84B expression (grayscale) in control (left) and *bru1-IR* (right) IFM at 48 h and 72 h APF and 1 d adult. Expression is only detected in 1 d adult *bru1-IR* IFM, but not earlier time points at 48 h and 72 h APF. All GFP-tagged reporters are under the control of endogenous regulatory elements for *wupA*, *Kettin*, *Clip190*, *Mlp60A*, and *Mlp84B*. GFP, green; phalloidin stained actin, magenta; scale bar = 5 μm. Underlying data can be found in S5 Fig Source Data as listed in S6 Table.
(TIFF)

S6 Fig RNAi knockdown of *bru1* with temporally regulated Gal4 drivers reveals differential requirement for Bru1 during IFM development. **(A)** Scheme of temporal Gal4 expression during IFM myogenesis. All Gal4 drivers tested in this study are listed on the left and ordered by expression time point. Colored bars depict the time range when each Gal4 driver is expressed. Gal4 drivers used for RNAi and rescue experiments depicted in main figures have a distinct color (*Him*-Gal4, tan; *UH3*-Gal4, turquoise; *Fln*-Gal4, yellow). Gradient color of the bar indicates the strength of temporal expression. Key time points in IFM myogenesis are marked at the bottom. **(B)** Quantification of the percent of flies that eclose from pupal cases in Gal4 controls and *bru1-IR*. No eclosion defect was noted for any of the *bru1-IR* lines tested. **(C)** Quantification of flight ability in Gal4 controls and *bru1-IR*^*salm*^, *bru1-IR*^*Act88F*^ and *bru1-IR*^*Fln*^ knockdown flies. *N* > 45 flies for each genotype. **(D)** Quantification of myofiber ripping and detachment phenotypes in Gal4 control and *bru1-IR*^*salm*^, *bru1-IR*^*Act88F*^, and *bru1-IR*^*Fln*^ knockdown flies at 90 h APF and in 1 d adult. *N* > 40 fiber for each genotype and time point. **(E–P)** Confocal projections of hemi-thoraxes showing DLMs of *salm*-Gal4, *Act88F*-Gal4, and *Fln*-Gal4 driven *bru1-IR* at 90 h APF (E–J) and 1 d adult (K–P). The myofibers of *salm*-Gal4 and *Act88F*-Gal4 driven *bru1-IR* are already ripped at 90 h APF (F, H), while *Fln*-Gal4 driven *bru1-IR* myofibers remain intact (J, P). Scale bar = 100 μm. **(E’–P’)** Single-plane confocal images of genotypes as in (E–P) showing myofibril and sarcomere phenotype of *bru1-IR* at 90 h APF (E’–J’) and 1 d adult (K’–P’). Scale bar = 5 μm. **(Q, R)** Quantification of sarcomere length (Q) and myofibril width (R) in (E’–P’). Boxplots are shown with Tukey whiskers, outlier data points marked as black dots. Significance determined across the time course by ANOVA and post hoc Tukey (ns, not significant; **P < 0.01; ***P < 0.001). Underlying data can be found in S6 Fig Source Data as listed in S6 Table.
(TIFF)

S7 Fig Validation of RNAi control crosses to *w*^*1118*^, UAS-GFP, and UAS-GFP-RNAi. **(A)** Quantification of flight ability in Gal4 drivers crossed to *w*^*1118*^ (magenta square), UAS-GFP (green triangle), or UAS-GFP-RNAi (yellow circle) at 27°C. High levels of Gal4 expression in Act88F-Gal4 at warm temperatures interfere with flight ability, but do not affect sarcomere length or width. *N* > 86 flies for each genotype. **(B–M)** Confocal projections of IFM myofiber structure (10× objective) and **(B’–M’)** single-plane confocal images of sarcomere structure (60× objective) for Fln-Gal4 (B, F, J), Him-Gal4 (C, G, K), Mef2-Gal4 (D, H, L), and UH3-Gal4 (E, I, M) crossed to *w*^*1118*^ (B–E), UAS-GFP (F–I), or UAS-GFP-RNAi (J–M). IFM myofibers are attached and sarcomeres have normal structure in all genotypes. **(N, O)** Quantification of sarcomere length (N) and myofibril width (O) in (B’–M’). Boxplots are shown with Tukey whiskers overlayed with all data points marked as black dots. Significance was determined by ANOVA and post hoc Tukey (ns, not significant). Sarcomere length and width are consistent in Gal4-alone as well as Gal4 driver crossed to UAS-GFP or UAS-GFP-RNAi. Underlying data can be found in S7 Fig Source Data as listed in S6 Table.
(TIFF)

S8 Fig Validation of *bru1* RNAi knockdown efficiency with different temporal Gal4 drivers at the mRNA and protein level. **(A)** RT-qPCR verification of *bru1* gene expression levels in mutant and knockdown conditions in 1 d adult IFM. Expression is shown relative to the matched control, either control *w*^*1118*^ or a Gal4 driver crossed to *w*^*1118*^. *bru1* levels were strongly and significantly reduced in *bru1*^*M3*^, *bru1-IR*, *bru1-IR*^*Him*^, *bru1-IR*^*UH3*^, and *bru1-IR*^*Fln*^. **(B–G)** Single-plane confocal images of IFM nuclei stained with rabbit anti-Bru1 in control, *bru1*^*M3*^, *bru1-IR*^*UH3*^, and *bru1-IR*^*Fln*^ at 90 h APF. Bru1 signal is absent in *bru1*^*M3*^ (C–C’) and *bru1-IR*^*UH3*^ (E–E’) IFM, but can still be detected in *bru1-IR*^*Fln*^ IFM (G–G’). Images were acquired using same settings and pseudo-colored based on intensity (B’–G’). Bru1, green; DAPI, magenta; scale bar = 5 μm. **(H)** Quantification of Bru1 relative signal intensity based on fluorescence levels in (B–G). Boxplots are shown with Tukey whiskers, outlier data points marked as black dots. Significance determined by ANOVA and post hoc Tukey in comparison to matched control (ns, not significant; ***P < 0.001). **(I–P)** Single-plane confocal images of IFM nuclei stained with rabbit anti-Bru1 in control and *bru1-IR*^*Him*^ at 24 h, 30 h, 48 h, and 90 h APF. Bru1signal is absent from *bru1-IR*^*Him*^ IFM at 24 h (M–M’) and 30 h (N–N’) APF, but can be detected at 48 h (O–O’) and 90 h (P–P’) APF. Images were acquired using same settings and pseudo-colored based on intensity (I’–P’). Bru1, green; DAPI, magenta; scale bar = 5 μm. **(Q)** Quantification of Bru1 relative signal intensity based on fluorescence levels in (I–P). Data visualized as in (H). Significance determined by ANOVA and post hoc Tukey in comparison to matched control (ns, not significant; **P < 0.01; ***P < 0.001). Underlying data can be found in S8 Fig Source Data as listed in S6 Table.
(TIFF)

S9 Fig Overexpression of Bru1 pre- or post-myofibrillogenesis generates strong fiber and myofibril phenotypes. **(A)** Diagram of the UAS-Bru1-RA (UAS-Bru1) expression construct integrated into the attP-86Fb landing site on chromosome 3R. The construct contains a 5× UAS-hsp70 promoter region, full-length *bru1-RA* coding sequence, and an SV40 terminator and polyadenylation sequence. **(B)** Quantification of the percent of flies that eclosed from control and UAS-Bru1 overexpression with *Him*-Gal4, *salm*-Gal4, *Act88F*-Gal4, *UH3*-Gal4, and *Fln*-Gal4. Overexpression with *salm*-Gal4 is embryonic lethal. **(C)** Quantification of flight ability in control and UAS-Bru1 overexpression with *Act88F*-Gal4 and *UH3*-Gal4. *N* > 30 flies for each genotype. Overexpression of Bru1 with Act88F-Gal4 or UH3-Gal4 caused loss of flight ability. **(D)** Quantification of myofiber tearing and detachment phenotypes at 90 h APF and 1 d adult in control and UAS-Bru1 overexpression with *Act88F*-Gal4 and *UH3*-Gal4. *N* > 40 fibers for each genotype and time point. Overexpression of Bru1 with Act88F-Gal4 leads to detached and severely degraded myofibers, while overexpression with UH3-Gal4 leads to a progressive hypercontraction phenotype. **(E–N)** Confocal projections of hemi-thoraxes from control and *Act88F*-Gal4 and *UH3*-Gal4 driven UAS-Bru1 at 90 h APF (E–I) and 1 d adult (J–N). Myofibers of *Act88F*-Gal4 Bru1 overexpression are fully degraded (G, L). Dashed line outlines the thorax boundaries in (G, L). UH3-Gal4 mediated overexpression of Bru1 results in abnormally short, thin and trapezoidal-shaped sarcomeres. Scale bar = 100 μm. **(E’–N’)** Single-plane confocal images showing myofibril and sarcomere phenotypes at 90 h APF (E’–I’) and 1 d adult (J’–N’). Scale bar = 5 μm. **(O, P)** Quantification of sarcomere length (O) and myofibril width (P) in (E’–N’). Boxplots are shown with Tukey whiskers, outlier data points marked as black dots. Significance determined for each time point by ANOVA and post hoc Tukey (ns, not significant; *P < 0.05; **P < 0.01; ***P < 0.001). Underlying data can be found in S9 Fig Source Data as listed in S6 Table.
(TIFF)

S10 Fig Expression of Bru1 with *Him*-Gal4 is early stage-specific and sufficient to generate phenotypes in control and *bru1*^*M3*^ IFM. **(A)** Single-plane confocal images of IFM nuclei stained with rabbit anti-Bru1 in control and *Him*-Gal4 overexpression of UAS-Bru1 at 24 h, 48 h, and 90 h APF. Bru1 signal is detected at 24 h APF, but not at 48 h or 90 h APF. Images were acquired using same settings and pseudo-colored based on intensity. Bru1, green; DAPI, magenta; scale bar = 5 μm. **(B)** Quantification of Bru1 relative signal intensity based on fluorescence levels in (A). Horizontal line denotes the mean value of Bru1 signal intensity in *bru1*^*M3*^. **(C)** Confocal projections of hemithorax and single plane images of IFM myofibrils at 48 h and 90 h APF and 1 d adult in control, *Him*-Gal4 driving UAS-Bru1, and *Him*-Gal4 driving UAS-Bru1 in a heterozygous mutant background (*bru1*^*M3/+*^). Phalloidin stained actin, gray; scale bar = 100 μm (hemithorax), or 5 μm (myofibrils). **(D)** Quantification of DLM fiber integrity at 90 h APF. Genotypes denoted by symbols: top row, *bru1* allele presence (wild-type *bru1*^*+/+*^, white square; heterozygous *bru1*^*+/-*^, half-red square, mutant *bru1*^*M3-/-*^, red square); middle row, *Him*-Gal4 driver presence (absent, empty; present, tan triangle); bottom row, UAS-Bru1 presence (absent, empty; present, magenta diamond). *N* > 40 fibers for each genotype. **(E, F)** Quantification of sarcomere length (E) and myofibril width (F) in (C). Genotypes denoted as in (D). Boxplots are shown with Tukey whiskers, outlier data points marked as black dots. Significance determined for each time point by ANOVA and post hoc Tukey (ns, not significant; *P < 0.05; ***P < 0.001). **(G)** Histological stain with hematoxylin and eosin (HE) in control, *bru1*^*M3*^, and *Him*-Gal4 rescue IFM myofibers at 48 h APF. Hole, yellow arrowheads; scale bar = 100 μm. **(H)** Quantification of myofiber morphology in (G). *N* > 10 for each genotype. **(I)** Quantification of myofibril width in (Fig 7Q–7T”). Data plotted and significance is determined as in (E, F). Underlying data can be found in S10 Fig Source Data as listed in S6 Table.
(TIFF)

S11 Fig Expression of Bru1 with *Fln*-Gal4 produces mild sarcomere defects in control but restores alternative splicing defects in *bru1*^*M3*^ IFM. **(A)** Single-plane confocal images of IFM nuclei stained with rabbit anti-Bru1 in control and *Fln*-Gal4 rescue of *bru1*^*M3*^ at 90 h APF. Images were acquired using same settings and pseudo-colored based on intensity. Bru1, green; DAPI, magenta; scale bar = 5 μm. **(B)** Quantification of Bru1 relative signal intensity based on fluorescence levels in (A). Statistical significance determined by unpaired *t* test (*P < 0.05). **(C)** Confocal projections of hemithorax and single plane images of myofibrils at 90 h APF and in 1 d adult in control, *Fln*-Gal4 driving UAS-Bru1, and *Fln*-Gal4 driving UAS-Bru1 in a heterozygous mutant background (*bru1*^*M3/+*^). Phalloidin stained actin, gray; scale bar = 100 μm (hemithorax), or 5 μm (myofibrils). **(D, E)** Quantification of sarcomere length (D) and myofibril width (E) in (C). Genotypes marked by symbols as in S9 Fig. Boxplots are shown with Tukey whiskers, outlier data points marked as black dots. Significance determined for each time point by ANOVA and post hoc Tukey (ns, not significant; ***P < 0.001). **(F)** Quantification of myofibril width in (Fig 8R) at 90 h APF. Significant determined as in (D, E). **(G, H)** Semi-quantitative RT-PCR verification of alternative splice events in *Zasp52* (G) and *Mhc* (H). Top: scheme of alternative isoforms with primer locations. Exon numbering in accordance with FB2021_05 annotation. Color coding of depicted isoforms consistent across top, middle, and bottom panels; 3′ UTR regions in light beige. Middle: Quantification of relative expression level of detectable events in control *w*^*1118*^ leg, jump (tergal depressor of the trochanter, TDT) and fibrillar IFM muscle, as well as *bru1*^*M3*^ and *Fln*-Gal4 rescue IFM. Error bars = SD. Bottom: representative RT-PCR gel image. **(I)** Semi-quantitative RT-PCR verification of alternative splice events in *Tm1*. Top: scheme of alternative isoforms. Bottom: representative RT-PCR gel image. Splice events in *Tm1* were detected with distinct reverse primers, as isoforms do not share a common 3′-UTR. Underlying data can be found in S11 Fig Source Data and Gels as listed in S6 Table.
(TIFF)

S12 Fig Summary table of *bru1* associated phenotypes in *Drosophila* IFM. IFM phenotypes are summarized in table format for all genotypes used in this study, as well as *bru1* mutant and RNAi phenotypes published previously [13,14,36,43,44]. Genotypes are as labeled. IFM phenotypes include early phenotypes in cytoskeletal rearrangement, myofiber compaction, and nascent myofibrils assayed in this study, as well as late pupal and adult phenotypes assayed in this and previous studies including flight ability, sarcomere, and myofibril structure; “loss of IFM-specific splice events” includes data from RT-PCR or mRNA-Seq, while “loss of IFM-specific protein isoforms” includes proteomics data and expression of GFP-tagged isoform reporters. Phenotypes were classified as strong (red), moderate (orange), mild (yellow), no phenotype (wild-type structure, white), or not tested/data not available (gray). Underlying data can be found throughout this manuscript and in references [13,14,36,43,44].
(TIFF)

S1 Table Tables listing the identity and values of data points shown in Fig 1H–1K. Data for each figure panel are contained in a separate tab in the Excel file. Data include which significantly DE peptides in proteomics data are also significantly misregulated in the DESeq2 and DEXSeq data from mRNA-Seq, the complete GO term enrichments and the DE values for sarcomere proteins (SPs) in the DESeq2, DEXSeq, and proteomics analyses.
(XLSX)

S2 Table Tables listing the identity and values of data points shown in Fig 2A, 2B and 2D. Data for each figure panel are contained in a separate tab in the Excel file. Data include a membership list for all annotated gene categories used in this manuscript, membership for genes in each region of the Venn Diagrams, and the DE analysis values from mRNA-Seq and proteomics used to plot the figure panels in Fig 2B and 2D.
(XLSX)

S3 Table Tables listing the identity and values of data points shown in Fig 4A–4F. Data for each figure panel are contained in a separate tab in the Excel file. Data include the DE analysis values used to generate all boxplots and line plots, the complete GO term analysis corresponding to Fig 4C, and the DESeq2 and DEXSeq analysis data for *bru1-IR* versus control IFM at 24 h, 30 h, 72 h, and 1 d adult.
(XLSX)

S4 Table Primer table. Complete list of all primer sequences used in this manuscript.
(XLSX)

S5 Table Key resources table. Complete list of all antibodies, fly lines, software packages, etc. used in this manuscript and their source.
(XLSX)

S6 Table Underlying data table. List of underlying data organized by figure panel and file name for each figure in this manuscript.
(XLSX)

S1 Movie Movie of spontaneous contractions at 48 h APF in wild-type and *bru1*^*M3*^ DLM myofibers. Spontaneous contractions in control and *bru1*^*M3*^ IFM at 48 h APF. From left to right: control DLM performing a single twitch; control DLM performing a double twitch; control DLM performing a triple twitch, *bru1*^*M3*^ DLM performing a single twitch. Timer, bottom left; scale bar = 50 μm.
(AVI)

S2 Movie Movie of spontaneous contractions at 72 h APF in wild-type and *bru1*^*M3*^ DLM myofibers. Control DLMs do not spontaneously contract at 72 h APF (left). A slow, one-directional contractile movement is observed in *bru1*^*M3*^ myofibers at 72 h APF (right). Timer, bottom left; scale bar = 50 μm.
(AVI)

S3 Movie Movie of a 3D reconstruction of GFP-Act88F integration into wild-type sarcomeres. Movie of an x,y,z-axis reconstruction from a confocal image of Fln-Gal4 driving UASp-GFP-Act88F in a control *w*^*1118*^ background. GFP labeling is restricted to a box-like pattern marking radial thin filament addition and actin integration into thin filaments after 56 h APF. GFP, green; phalloidin stained actin, red.
(AVI)

S4 Movie Movie of a 3D reconstruction of GFP-Act88F integration into *bru1*^*M3*^ mutant sarcomeres. Movie of an x,y,z-axis reconstruction from a confocal image of Fln-Gal4 driving UASp-GFP-Act88F in a *bru1*^*M3*^ background. GFP labeling is altered in comparison to wild-type, revealing a lack of GFP-Act88F integration into thin filaments added radially after 56 h APF and abnormal actin integration across the entire thin filament with enrichment at the z-disc and M-line. GFP, green; phalloidin stained actin, red.
(AVI)

S5 Movie Movie of a 3D reconstruction of Mhc^Wee-P26^-GFP integration into wild-type sarcomeres. Movie of an x,y,z-axis reconstruction from a confocal image of Mhc-weeP26-GFP in a control background. GFP signal is restricted to two dots flanking the M-line, reflecting the first bipolar myosin filaments assembled into pre-myofibrils. GFP, green; phalloidin stained actin, red.
(MP4)

S6 Movie Movie of a 3D reconstruction of Mhc^Wee-P26^-GFP integration into *bru1*^*M3*^ mutant sarcomeres. Movie of an x,y,z-axis reconstruction from a confocal image of Mhc-weeP26-GFP in a *bru1*^*M3*^ background. GFP signal is no longer restricted to the pre-myofibril myosin elements, and instead integrated across the sarcomere. GFP, green; phalloidin stained actin, red.
(MP4)

S1 Raw Images Raw images. (PDF)

## Abbreviations

APF: after puparium formation
CELF: CUG-BP- and ETR-3-like factor
DE: differentially expressed
DLM: dorsal-longitudinal myofiber
DVM: dorsal-ventral myofiber
GO: gene ontology
IFM: indirect flight muscle
PSF: point spread function
RRM: RNA recognition motif
SP: sarcomere protein
TEM: transmission electron microscopy
URE: untranslated regulatory element
