# Supplementary figures and images for: Bruno 1/CELF regulates splicing and cytoskeleton dynamics to ensure correct sarcomere assembly in Drosophila flight muscles
Source: PLoS Biol. 2024 Apr 29;22(4):e3002575. doi: 10.1371/journal.pbio.3002575 (PMC11081514; doi:10.1371/journal.pbio.3002575)

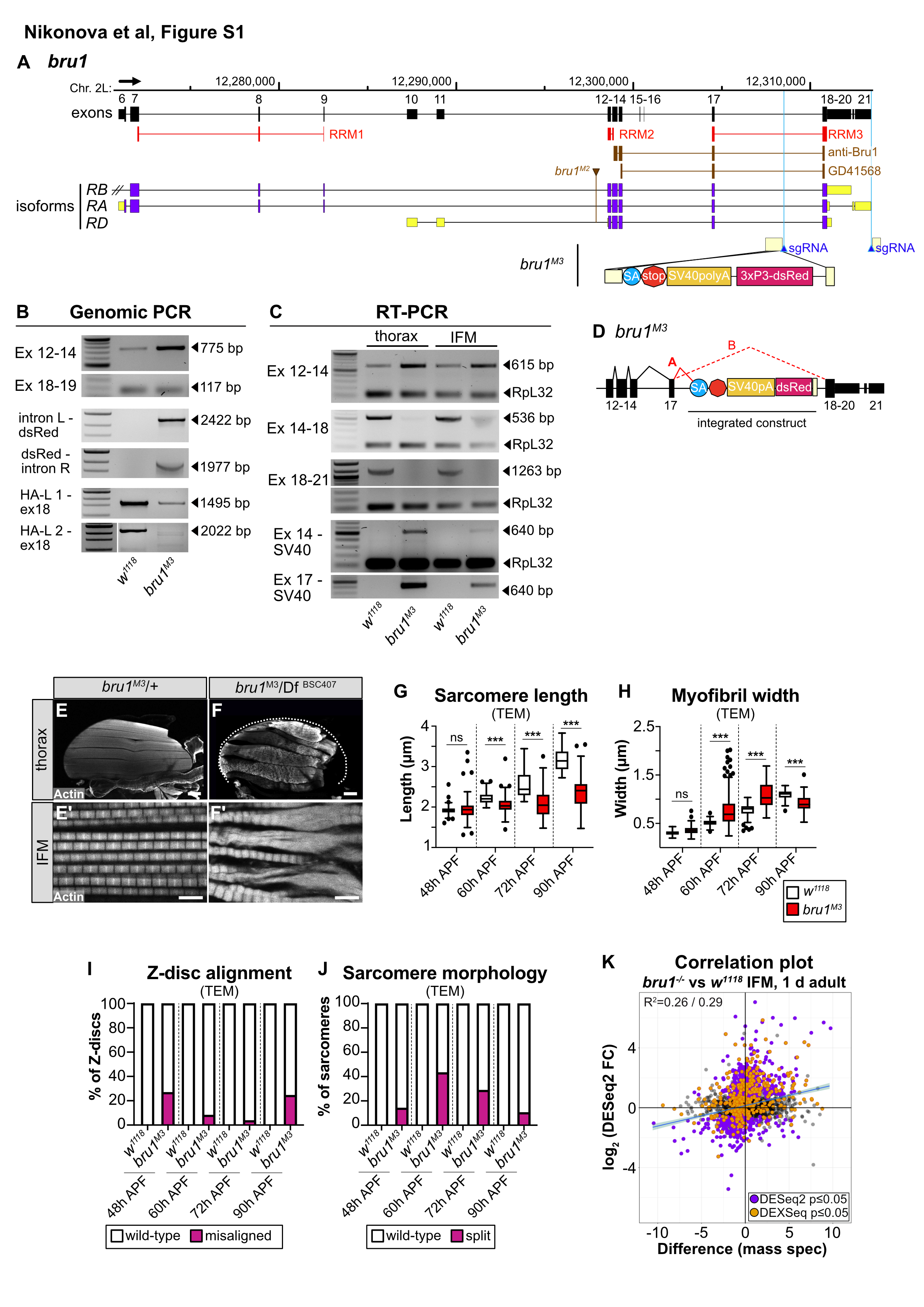

Supplement: S1 Fig — (A) Diagram of the C-terminal region of the bruno1 (bru1) locus and mRNA isoforms RA, RB, and RD (exons, purple; UTRs, yellow). Location of the RNA recognition motif domains (RRM, light red), target region of anti-Bru1 antibody (brown), target region of bru1-IR GD41568 hairpin (brown), location of bru1M2 construct insertion site (brown), and the sgRNAs (blue) used for CRISPR-mediated generation of the bru1M3 hypomorph allele are marked. Transgenic construct is inserted upstream of exon 18 and contains a strong splice acceptor (SA, light blue), a triple frame stop (stop, red), an SV40 polyadenylation signal (orange) and a selectable 3xP3-dsRed marker (crimson) flanked by homology arms (light tan). Exon numbering according to the annotation FB2021-05. (B) Whole-fly genomic PCR verifying dsRed cassette insertion in the bru1 locus. Identity of amplified region marked on the left, band size noted on the right. Primer sequences available in S4 Table. (C) RT-PCR to test expression of bru1 mRNA in whole-thorax and dissected IFM. Identity of amplified region marked on the left, band size noted on the right. RpL32 used as internal control. (D) Diagram of the bru1M3 allele. Splicing from exon 17 is redirected into the splice acceptor (SA) of the inserted construct (red line, A), leading to early termination of the bru1 mRNA and truncation of RRM3. Splicing from exon 17 to exon 18 is strongly reduced (dotted red line, B), and signal from 3′-UTR exon 21 is not detectable. (E, F) Confocal projections of 1d adult hemithoraxes showing IFM from bru1M3/+ and bru1M3/Df(2L)BSC407. Deficiency BSC407 covers the complete bru1 locus. Thorax boundaries in (F), dashed line; phalloidin stained actin, gray; scale bar = 100 μm. (E’–F’) Single-plane confocal images of 1 d adult IFM myofibrils. Scale bar = 5 μm. (G, H) Quantification of sarcomere length (G) and myofibril width (H) from TEM data shown in Fig 1F. Boxplots are shown with Tukey whiskers, outlier data points marked as black dots. Sign [file pbio.3002575.s001.tiff]

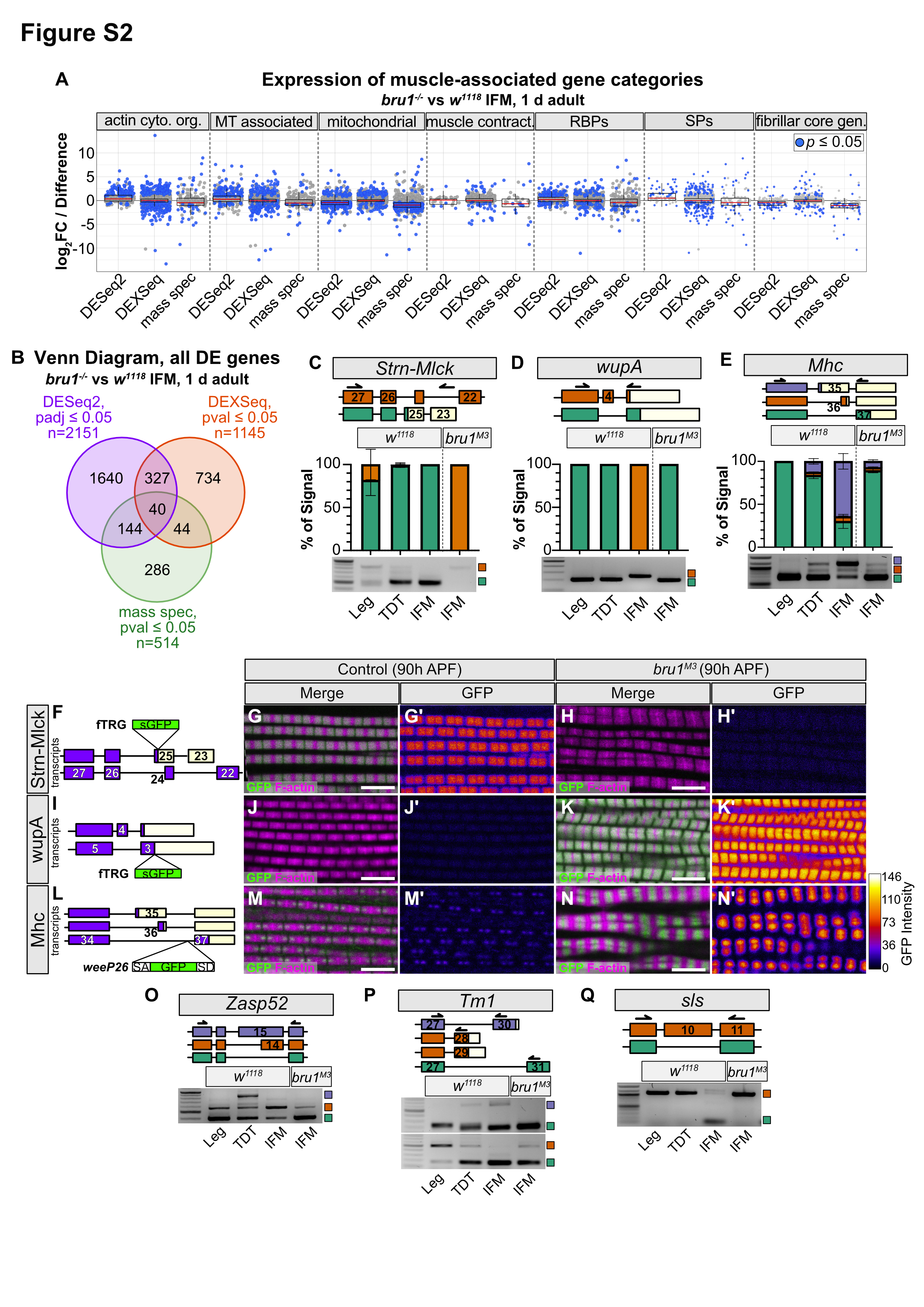

Supplement: S2 Fig — (A) Boxplot of gene (DESeq2), exon (DEXSeq) and protein level (mass spec) expression changes between 1 d adult bru1-/- and w1118 IFM in select categories of genes including GO term “actin cytoskeleton organization,” microtubule associated genes, mitochondrial genes, GO term “muscle contraction,” RNA-binding proteins (RBPs), sarcomere proteins (SPs), and fibrillar core genes. Blue dot denotes p ≤ 0.05. (B) Venn diagram of the overlap between all significantly DE genes (purple), exons (orange), and proteins (green) between bru1-/- versus w1118 IFM in 1 d adults. (C–E) Semi-quantitative RT-PCR verification of alternative splice events in Strn-Mlck (C), wupA (D), and Mhc (E). Top: scheme of alternative isoforms with primer locations. Exon numbering in accordance with the FB2021_05 annotation. Color coding of depicted isoforms consistent with bottom panel; 3′-UTR regions in light beige. Middle: Quantification of relative expression level of splice events in tubular leg and jump (tergal depressor of the trochanter, TDT) and fibrillar IFM in control flies and in bru1M3 IFM. Error bars = SD. Bottom: representative RT-PCR gel image. (F–N’) Misexpression of GFP-tagged sarcomere proteins in bru1M3 IFM. (F, I, L) Diagrams of reporter GFP incorporation into tagged transcripts of Strn-Mlck (F), wupA (I), and Mhc-weeP26-GFP (L). Exons, magenta; 3′-UTR, tan; SA, splice acceptor; SD, splice donor; sGFP, superfold GFP. (G–N) Intensity matched single-plane confocal images from control and bru1M3 IFM at 90 h APF showing incorporation of Strn-Mlck-IsoR-GFP (G, H), wupA-GFP (J, K), and Mhc-weeP26-GFP (M, N). Strn-Mlck isoform R with sGFP tagged exon 25 is strongly expressed in wild-type IFM (G’) but absent from bru1M3 (H’). WupA with an sGFP tagged exon 3 is normally absent from wild-type IFM (J’) but gained in bru1M3 (K’). Expression of the Mhc isoform containing exon 37 and tagged in weeP26-GFP is normally restricted to early IFM development (M–M’), but is altered in bru1M3 (N–N’). GFP [file pbio.3002575.s002.tiff]

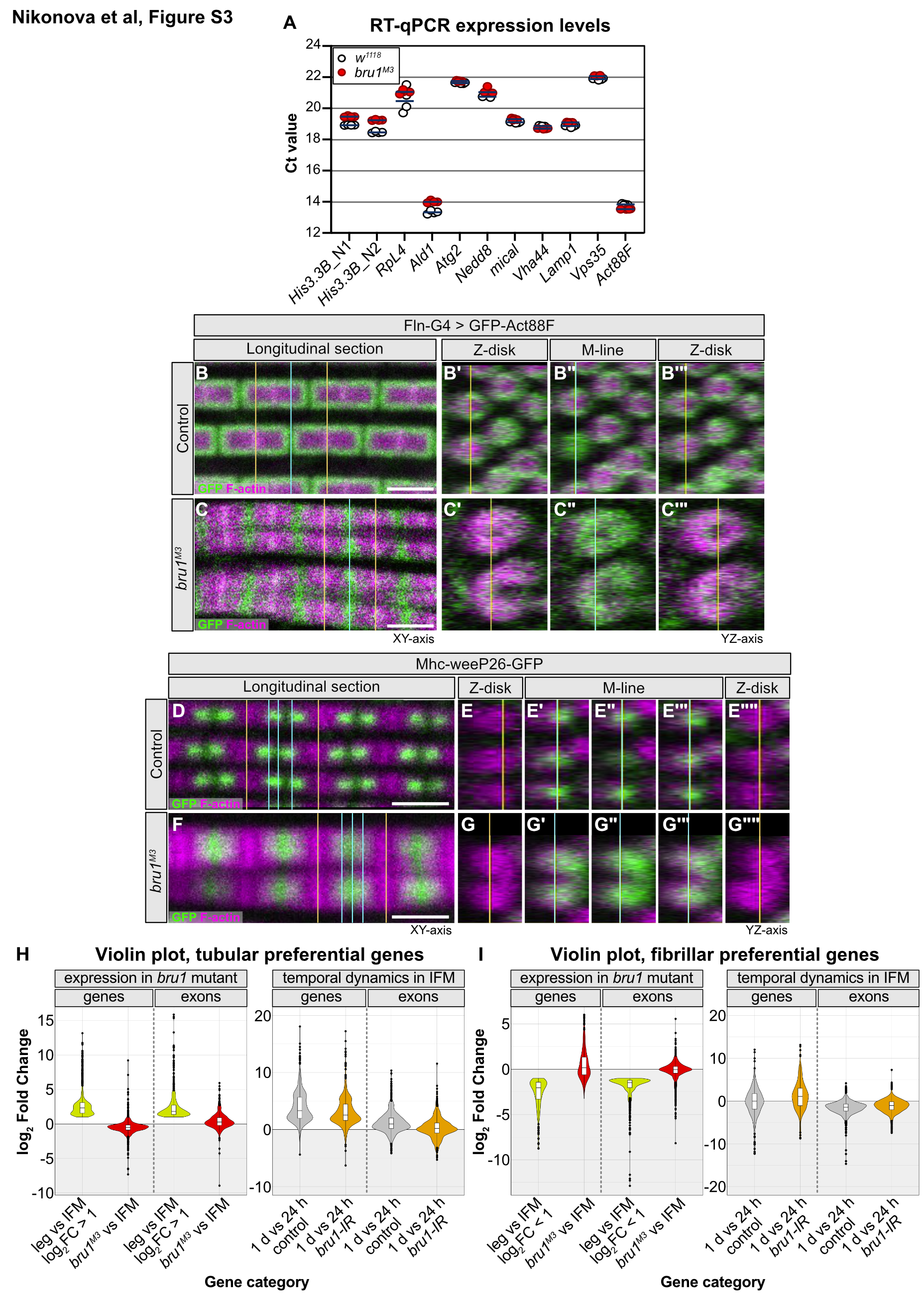

Supplement: S3 Fig — (A) mRNA expression level raw CT values assayed by RT-qPCR for different genes in bru1M3 (red dots) and w1118 (white dots) IFM, including Act88F, Vps35, Lamp1, Vha44, mical, Nedd8, Atg2, Ald1, and His3.3B (2 independent primer sets: N1 and N2, were used). Some genes, such as His3.3B and Ald1 are changed in bru1M3, and not suited as a normalization standard. (B–C”‘) Single plane confocal images of Fln-Gal4 driven pUAS-GFP-Actin88F incorporation into control (B–B’”) and bru1M3 (C–C’”) at 90 h APF. Longitudinal sections (B, C) represent the XY-axis, while vertical lines mark the exact position of orthogonal slices at the z-disc (yellow line) and M-line (cyan line). Orthogonal view (B’–B’”, C’–C’”) represents YZ-axis of (B, C), respectively. GFP, green; phalloidin stained actin, magenta; scale bar = 5 μm. (D–G”‘) Single-plane confocal images of Mhc-weeP26-GFP expression in control (D–E””) and bru1M3 (F–G””) at 90 h APF. Mhc-weeP26-GFP labels a specific isoform of Mhc that is only expressed during early IFM development. Longitudinal (D, F) and orthogonal sections (E–E””, G–G””) are shown as above at the z-disc (yellow line) and M-line (blue lines). GFP, green; phalloidin stained actin, magenta; scale bar = 5 μm. (H) Violin plots of changes in expression of tubular-preferential genes and exons. Left plot shows changes in tubular-preferential gene and exon expression in tubular leg versus fibrillar wild-type IFM (yellow) and bru1M3 versus wild-type IFM (red). Tubular-preferential was defined as all genes or exons with a log2FC > 1 and an adjusted p-value < 0.05 in the leg versus IFM comparison. Some but not the majority of tubular genes and exons are up-regulated in bru1M3 IFM. Right plot shows how the same tubular-preferential gene/exon sets change expression with time in IFM when comparing 1 d adult IFM to 24 h APF IFM in control (gray) or bru1-IR (orange) IFM. (I) Violin plots of changes in expression of fibrillar-preferential genes and exons. Left plot shows changes in [file pbio.3002575.s003.tiff]

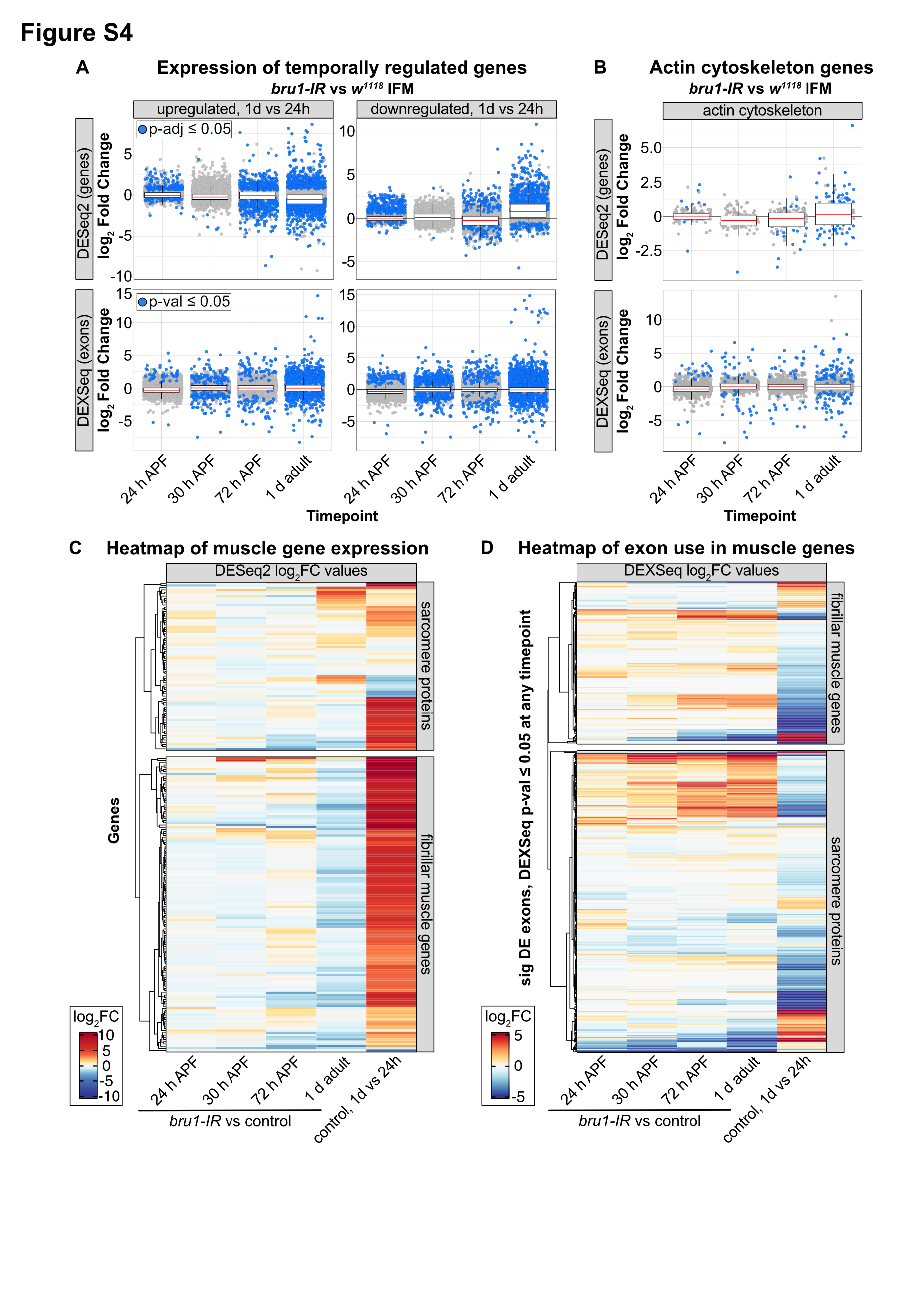

Supplement: S4 Fig — (A) Top: Boxplot of changes in gene expression across the bru1-IR time course (bru1-IR versus control) for temporal-switch genes that are normally up-regulated (log2FC > 0, p-adj ≤ 0.05) or down-regulated (log2FC < 0, p-adj ≤ 0.05) in control IFM from 24 h APF to 1 d adult. Bottom: Boxplot of changes in exon use across the bru1-IR time course for temporal-switch exons that are normally up-regulated (log2FC > 0, p-value ≤ 0.05) or down-regulated (log2FC < 0, p-value ≤ 0.05) in control IFM from 24 h APF to 1 d adult. Blue dot denotes p ≤ 0.05. (B) Boxplot of changes in gene expression (DESeq2) and exon use (DEXSeq) in GO term “actin cytoskeleton” genes in bru1-IR versus control IFM at 24 h, 30 h, 72 h APF, and in 1 d adult. Blue dot denotes p ≤ 0.05. (C) Heatmap of gene level-expression changes in all sarcomere protein and fibrillar muscle genes at all time points in bru1-IR versus control IFM. The fifth column shows the temporal change in use of the same genes in wild-type IFM from 24 h APF to 1 d adult. (D) Heatmap of all exons significantly DE (DEXSeq, p-val ≤ 0.05) at any time point in bru1-IR versus control IFM. The fifth column shows the temporal change in use of the same exons in wild-type IFM from 24 h APF to 1 d adult. Underlying data can be found in S2 Table, and the RNA-Seq data tables as listed in S6 Table. (TIFF) [file pbio.3002575.s004.tiff]

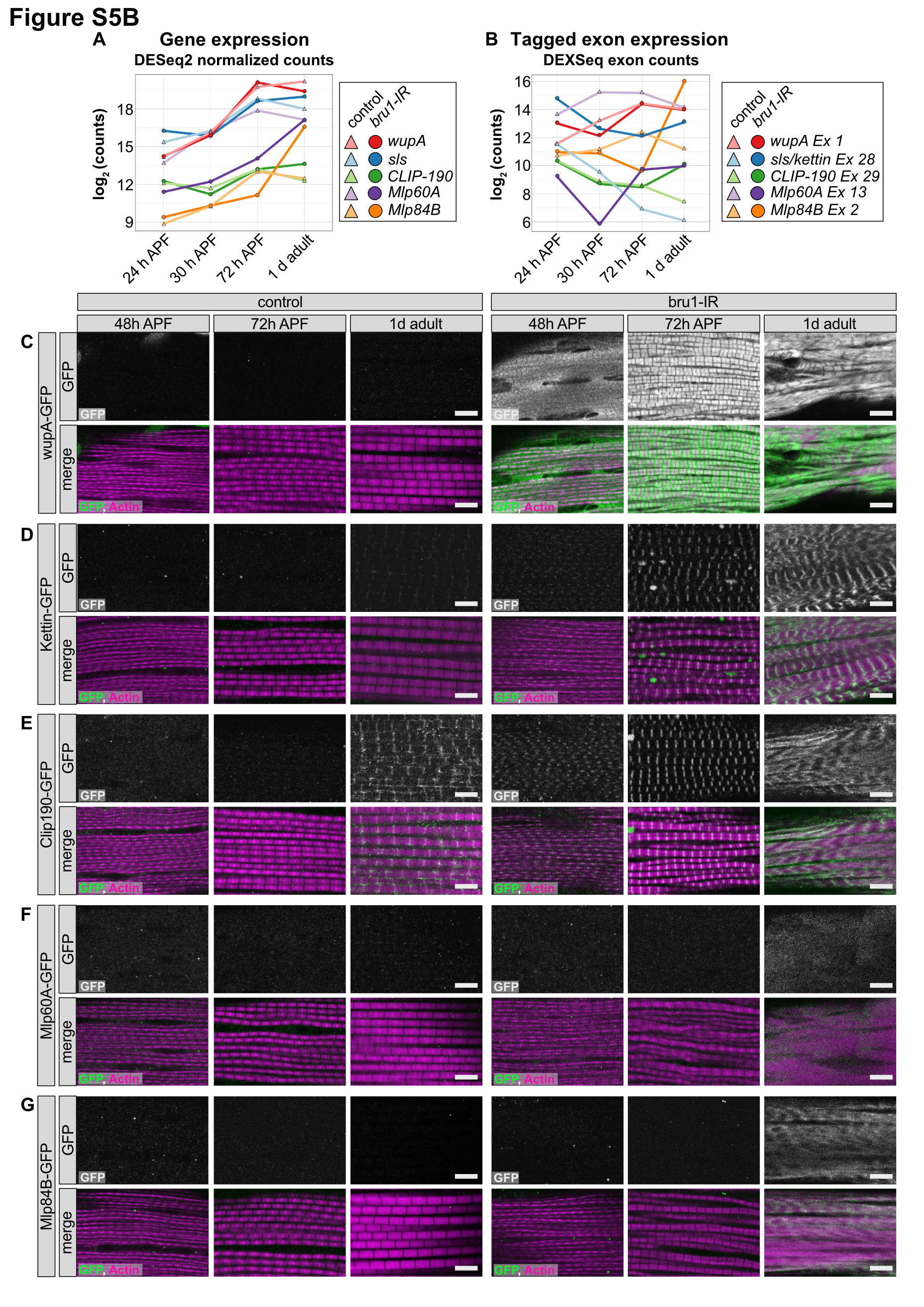

Supplement: S5 Fig — (A) Plot of mRNA-Seq based gene-level expression of wupA (maroon), sls (blue), CLIP-190 (green), Mlp60A (purple) and Mlp84B (orange) in bru1-IR (dark colors) and control (light colors) IFM at 24 h, 30 h, and 72 h APF and in 1 d adult. log2(counts) of DESeq2 count values normalized across all 4 time points are plotted. Gene expression levels of wupA, Kettin, and Clip190 are consistent between control and bru1-IR across the mRNA time course. Both Mlp60A and Mlp84B encode a single protein isoform that shows strong up-regulation at the gene level in bru1-IR IFM from 72 h APF to 1 d adult. (B) Plot of mRNA-Seq based exon-level expression of wupA exon 1 (maroon), sls exon 38 (blue), CLIP-190 exon 29 (green), Mlp60A exon 13 (purple), and Mlp84B exon 2 (orange) in bru1-IR (dark colors) and control (light colors) IFM at 24 h, 30 h, and 72 h APF and in 1 d adult. There are differences in the use of the isoform containing the exon where the GFP tag is inserted in wupA, Kettin, and Clip190. log2(counts) of DEXSeq normalized count values are plotted. The selected exons contain the GFP-tag visualized in (C–G). (C–E) Expression of select splice-isoforms of wupA (C), sls (D), and CLIP-190 (E) visualized by GFP-tag fluorescence (grayscale) in intensity-matched, single-plane confocal micrographs of IFM from control (left) and bru1-IR (right) flies at 48 h and 72 h APF and 1 d adult. wupA-GFP, which labels a termination used preferentially in tubular muscle [14] (see also S2D, S2I, S2J’, and S2K’), is already visible in bru1-IR but not control IFM at 48 h and 72 h APF (C), confirming its missplicing throughout development in bru1-IR IFM. Kettin encodes a short isoform of sls that is preferentially expressed in tubular muscle [162]. The GFP-tag in sls labels the Kettin isoform. Kettin-GFP is not expressed in control IFM, but is observed in bru1-IR IFM from 72 h APF (D). A GFP tag inserted in exon 29 of Clip-190 is only expressed weakly in adult IFM in control, but is already expressed [file pbio.3002575.s005.tiff]

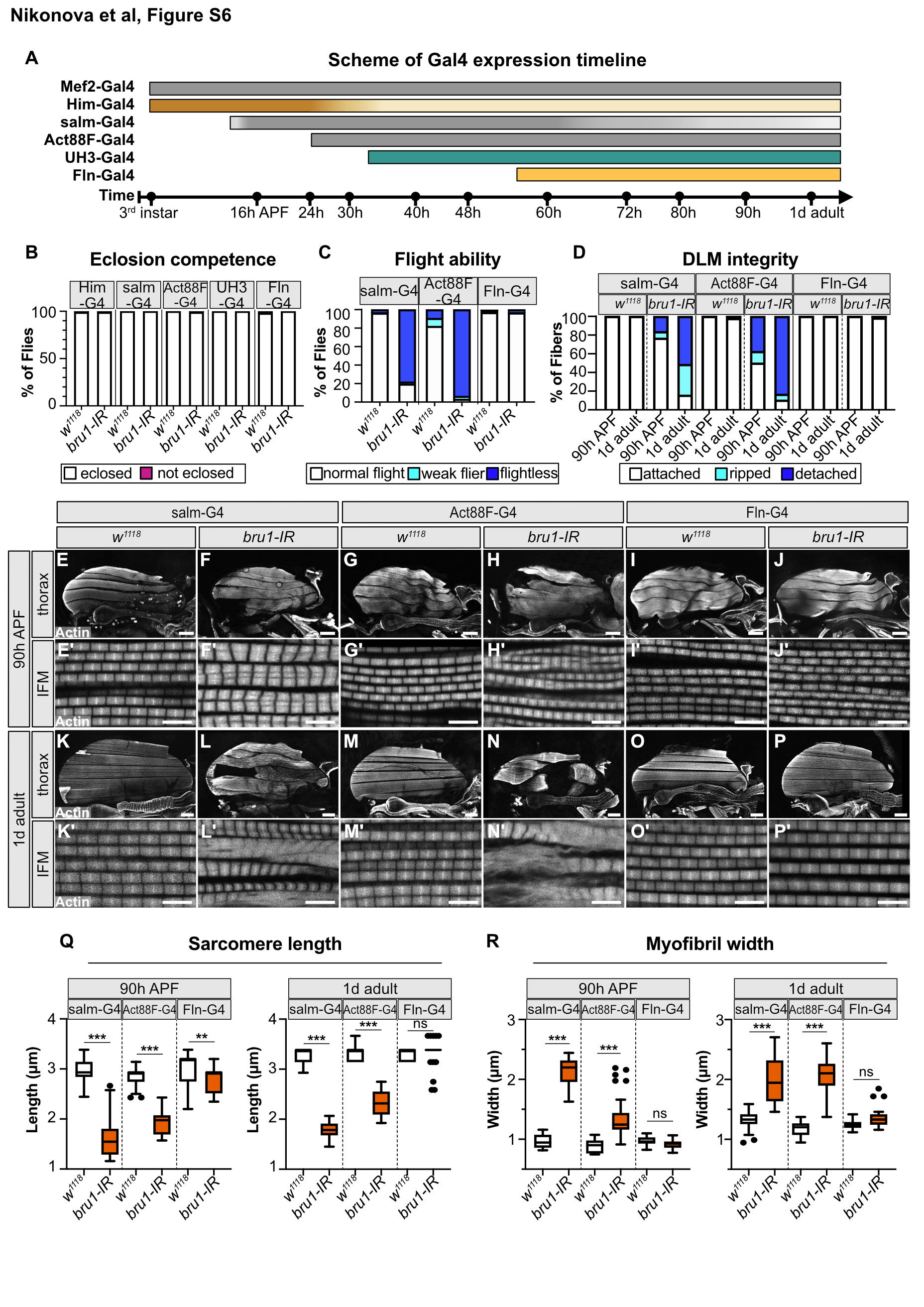

Supplement: S6 Fig — (A) Scheme of temporal Gal4 expression during IFM myogenesis. All Gal4 drivers tested in this study are listed on the left and ordered by expression time point. Colored bars depict the time range when each Gal4 driver is expressed. Gal4 drivers used for RNAi and rescue experiments depicted in main figures have a distinct color (Him-Gal4, tan; UH3-Gal4, turquoise; Fln-Gal4, yellow). Gradient color of the bar indicates the strength of temporal expression. Key time points in IFM myogenesis are marked at the bottom. (B) Quantification of the percent of flies that eclose from pupal cases in Gal4 controls and bru1-IR. No eclosion defect was noted for any of the bru1-IR lines tested. (C) Quantification of flight ability in Gal4 controls and bru1-IRsalm, bru1-IRAct88F and bru1-IRFln knockdown flies. N > 45 flies for each genotype. (D) Quantification of myofiber ripping and detachment phenotypes in Gal4 control and bru1-IRsalm, bru1-IRAct88F, and bru1-IRFln knockdown flies at 90 h APF and in 1 d adult. N > 40 fiber for each genotype and time point. (E–P) Confocal projections of hemi-thoraxes showing DLMs of salm-Gal4, Act88F-Gal4, and Fln-Gal4 driven bru1-IR at 90 h APF (E–J) and 1 d adult (K–P). The myofibers of salm-Gal4 and Act88F-Gal4 driven bru1-IR are already ripped at 90 h APF (F, H), while Fln-Gal4 driven bru1-IR myofibers remain intact (J, P). Scale bar = 100 μm. (E’–P’) Single-plane confocal images of genotypes as in (E–P) showing myofibril and sarcomere phenotype of bru1-IR at 90 h APF (E’–J’) and 1 d adult (K’–P’). Scale bar = 5 μm. (Q, R) Quantification of sarcomere length (Q) and myofibril width (R) in (E’–P’). Boxplots are shown with Tukey whiskers, outlier data points marked as black dots. Significance determined across the time course by ANOVA and post hoc Tukey (ns, not significant; **P < 0.01; ***P < 0.001). Underlying data can be found in S6 Fig Source Data as listed in S6 Table. (TIFF) [file pbio.3002575.s006.tiff]

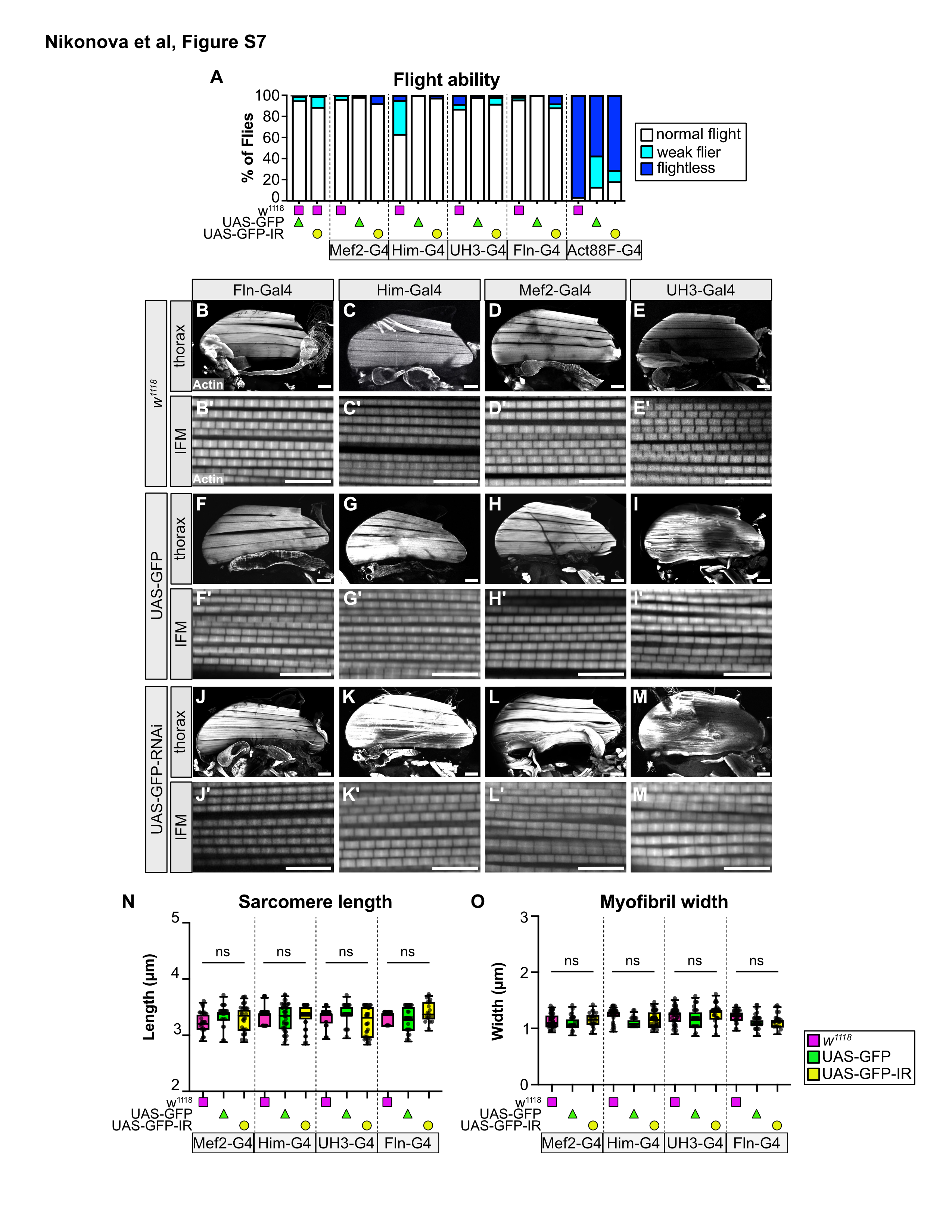

Supplement: S7 Fig — (A) Quantification of flight ability in Gal4 drivers crossed to w1118 (magenta square), UAS-GFP (green triangle), or UAS-GFP-RNAi (yellow circle) at 27°C. High levels of Gal4 expression in Act88F-Gal4 at warm temperatures interfere with flight ability, but do not affect sarcomere length or width. N > 86 flies for each genotype. (B–M) Confocal projections of IFM myofiber structure (10× objective) and (B’–M’) single-plane confocal images of sarcomere structure (60× objective) for Fln-Gal4 (B, F, J), Him-Gal4 (C, G, K), Mef2-Gal4 (D, H, L), and UH3-Gal4 (E, I, M) crossed to w1118 (B–E), UAS-GFP (F–I), or UAS-GFP-RNAi (J–M). IFM myofibers are attached and sarcomeres have normal structure in all genotypes. (N, O) Quantification of sarcomere length (N) and myofibril width (O) in (B’–M’). Boxplots are shown with Tukey whiskers overlayed with all data points marked as black dots. Significance was determined by ANOVA and post hoc Tukey (ns, not significant). Sarcomere length and width are consistent in Gal4-alone as well as Gal4 driver crossed to UAS-GFP or UAS-GFP-RNAi. Underlying data can be found in S7 Fig Source Data as listed in S6 Table. (TIFF) [file pbio.3002575.s007.tiff]

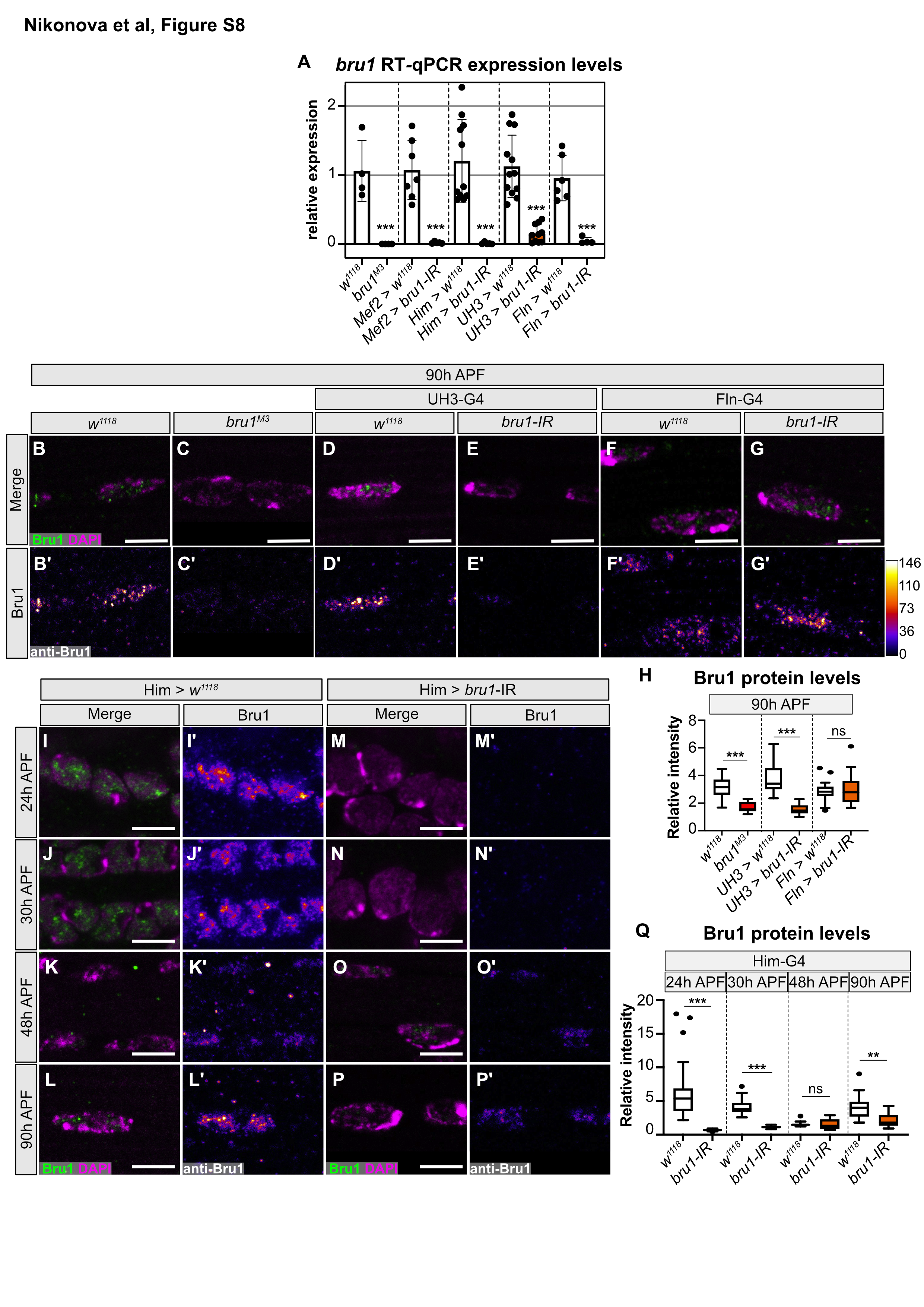

Supplement: S8 Fig — (A) RT-qPCR verification of bru1 gene expression levels in mutant and knockdown conditions in 1 d adult IFM. Expression is shown relative to the matched control, either control w1118 or a Gal4 driver crossed to w1118. bru1 levels were strongly and significantly reduced in bru1M3, bru1-IR, bru1-IRHim, bru1-IRUH3, and bru1-IRFln. (B–G) Single-plane confocal images of IFM nuclei stained with rabbit anti-Bru1 in control, bru1M3, bru1-IRUH3, and bru1-IRFln at 90 h APF. Bru1 signal is absent in bru1M3 (C–C’) and bru1-IRUH3 (E–E’) IFM, but can still be detected in bru1-IRFln IFM (G–G’). Images were acquired using same settings and pseudo-colored based on intensity (B’–G’). Bru1, green; DAPI, magenta; scale bar = 5 μm. (H) Quantification of Bru1 relative signal intensity based on fluorescence levels in (B–G). Boxplots are shown with Tukey whiskers, outlier data points marked as black dots. Significance determined by ANOVA and post hoc Tukey in comparison to matched control (ns, not significant; ***P < 0.001). (I–P) Single-plane confocal images of IFM nuclei stained with rabbit anti-Bru1 in control and bru1-IRHim at 24 h, 30 h, 48 h, and 90 h APF. Bru1signal is absent from bru1-IRHim IFM at 24 h (M–M’) and 30 h (N–N’) APF, but can be detected at 48 h (O–O’) and 90 h (P–P’) APF. Images were acquired using same settings and pseudo-colored based on intensity (I’–P’). Bru1, green; DAPI, magenta; scale bar = 5 μm. (Q) Quantification of Bru1 relative signal intensity based on fluorescence levels in (I–P). Data visualized as in (H). Significance determined by ANOVA and post hoc Tukey in comparison to matched control (ns, not significant; **P < 0.01; ***P < 0.001). Underlying data can be found in S8 Fig Source Data as listed in S6 Table. (TIFF) [file pbio.3002575.s008.tiff]

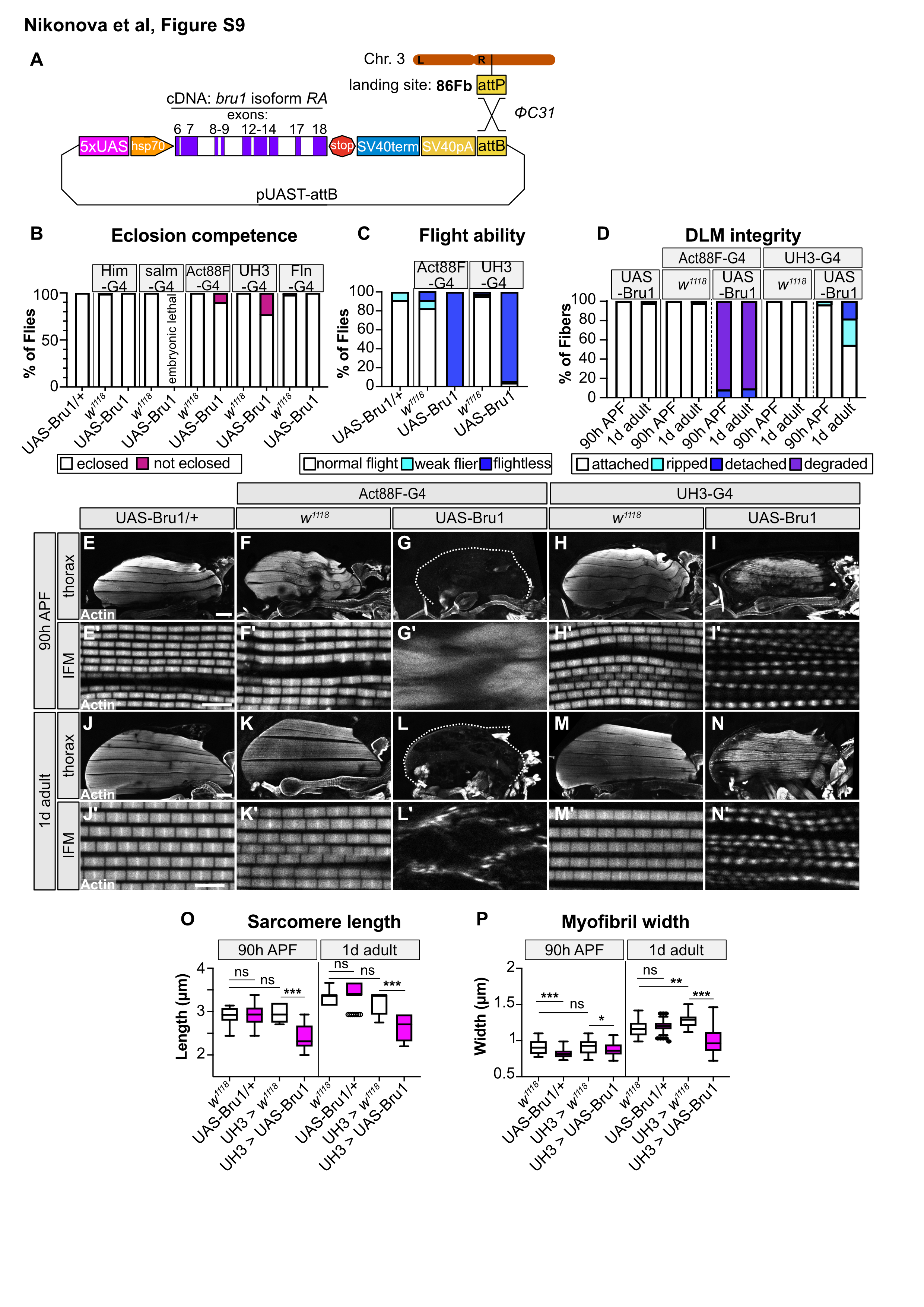

Supplement: S9 Fig — (A) Diagram of the UAS-Bru1-RA (UAS-Bru1) expression construct integrated into the attP-86Fb landing site on chromosome 3R. The construct contains a 5× UAS-hsp70 promoter region, full-length bru1-RA coding sequence, and an SV40 terminator and polyadenylation sequence. (B) Quantification of the percent of flies that eclosed from control and UAS-Bru1 overexpression with Him-Gal4, salm-Gal4, Act88F-Gal4, UH3-Gal4, and Fln-Gal4. Overexpression with salm-Gal4 is embryonic lethal. (C) Quantification of flight ability in control and UAS-Bru1 overexpression with Act88F-Gal4 and UH3-Gal4. N > 30 flies for each genotype. Overexpression of Bru1 with Act88F-Gal4 or UH3-Gal4 caused loss of flight ability. (D) Quantification of myofiber tearing and detachment phenotypes at 90 h APF and 1 d adult in control and UAS-Bru1 overexpression with Act88F-Gal4 and UH3-Gal4. N > 40 fibers for each genotype and time point. Overexpression of Bru1 with Act88F-Gal4 leads to detached and severely degraded myofibers, while overexpression with UH3-Gal4 leads to a progressive hypercontraction phenotype. (E–N) Confocal projections of hemi-thoraxes from control and Act88F-Gal4 and UH3-Gal4 driven UAS-Bru1 at 90 h APF (E–I) and 1 d adult (J–N). Myofibers of Act88F-Gal4 Bru1 overexpression are fully degraded (G, L). Dashed line outlines the thorax boundaries in (G, L). UH3-Gal4 mediated overexpression of Bru1 results in abnormally short, thin and trapezoidal-shaped sarcomeres. Scale bar = 100 μm. (E’–N’) Single-plane confocal images showing myofibril and sarcomere phenotypes at 90 h APF (E’–I’) and 1 d adult (J’–N’). Scale bar = 5 μm. (O, P) Quantification of sarcomere length (O) and myofibril width (P) in (E’–N’). Boxplots are shown with Tukey whiskers, outlier data points marked as black dots. Significance determined for each time point by ANOVA and post hoc Tukey (ns, not significant; *P < 0.05; **P < 0.01; ***P < 0.001). Underlying data can be found in S9 Fig Source Data as listed in S6 Table. (TIF [file pbio.3002575.s009.tiff]

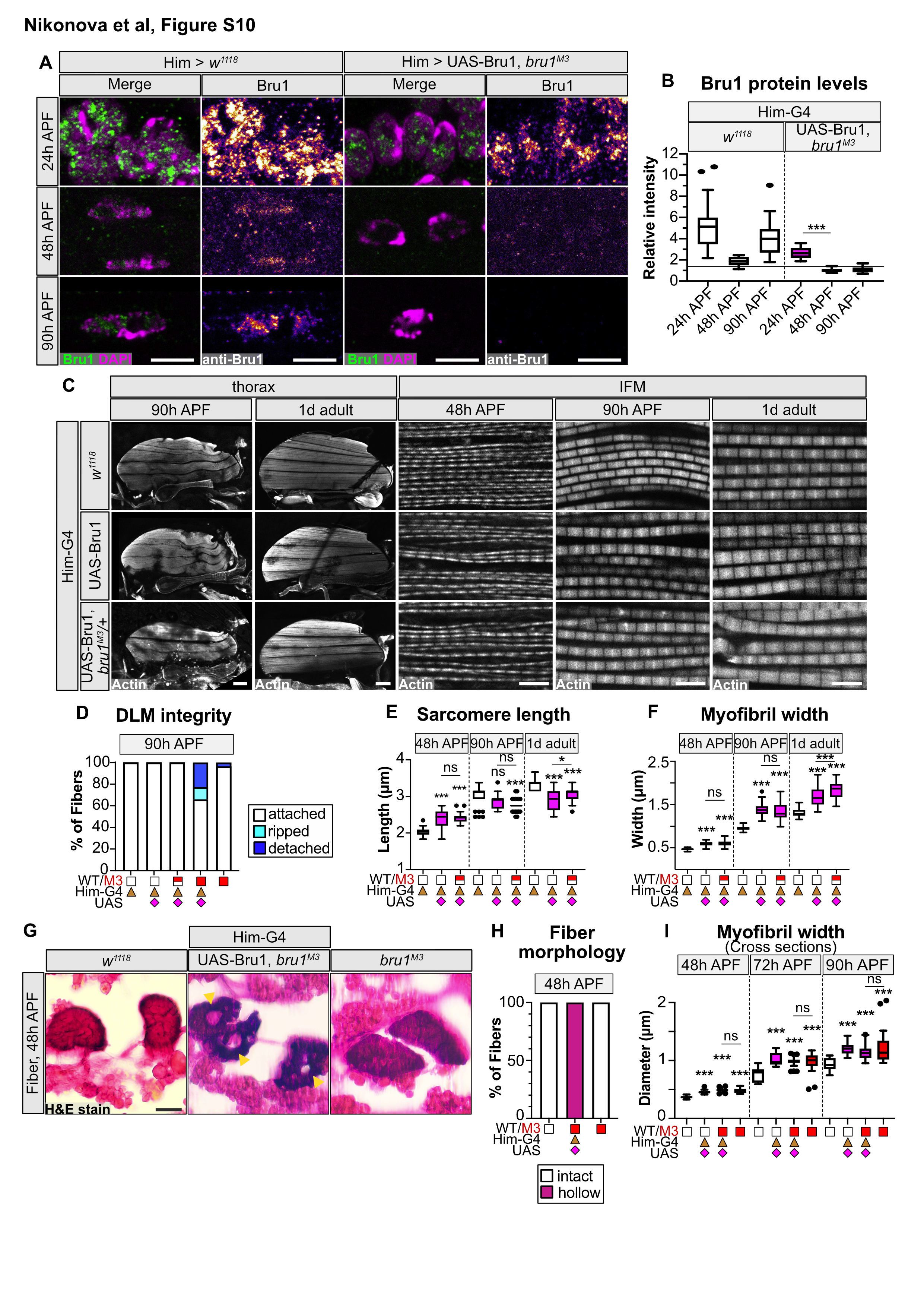

Supplement: S10 Fig — (A) Single-plane confocal images of IFM nuclei stained with rabbit anti-Bru1 in control and Him-Gal4 overexpression of UAS-Bru1 at 24 h, 48 h, and 90 h APF. Bru1 signal is detected at 24 h APF, but not at 48 h or 90 h APF. Images were acquired using same settings and pseudo-colored based on intensity. Bru1, green; DAPI, magenta; scale bar = 5 μm. (B) Quantification of Bru1 relative signal intensity based on fluorescence levels in (A). Horizontal line denotes the mean value of Bru1 signal intensity in bru1M3. (C) Confocal projections of hemithorax and single plane images of IFM myofibrils at 48 h and 90 h APF and 1 d adult in control, Him-Gal4 driving UAS-Bru1, and Him-Gal4 driving UAS-Bru1 in a heterozygous mutant background (bru1M3/+). Phalloidin stained actin, gray; scale bar = 100 μm (hemithorax), or 5 μm (myofibrils). (D) Quantification of DLM fiber integrity at 90 h APF. Genotypes denoted by symbols: top row, bru1 allele presence (wild-type bru1+/+, white square; heterozygous bru1+/-, half-red square, mutant bru1M3-/-, red square); middle row, Him-Gal4 driver presence (absent, empty; present, tan triangle); bottom row, UAS-Bru1 presence (absent, empty; present, magenta diamond). N > 40 fibers for each genotype. (E, F) Quantification of sarcomere length (E) and myofibril width (F) in (C). Genotypes denoted as in (D). Boxplots are shown with Tukey whiskers, outlier data points marked as black dots. Significance determined for each time point by ANOVA and post hoc Tukey (ns, not significant; *P < 0.05; ***P < 0.001). (G) Histological stain with hematoxylin and eosin (HE) in control, bru1M3, and Him-Gal4 rescue IFM myofibers at 48 h APF. Hole, yellow arrowheads; scale bar = 100 μm. (H) Quantification of myofiber morphology in (G). N > 10 for each genotype. (I) Quantification of myofibril width in (Fig 7Q–7T”). Data plotted and significance is determined as in (E, F). Underlying data can be found in S10 Fig Source Data as listed in S6 Table. (TIFF) [file pbio.3002575.s010.tiff]

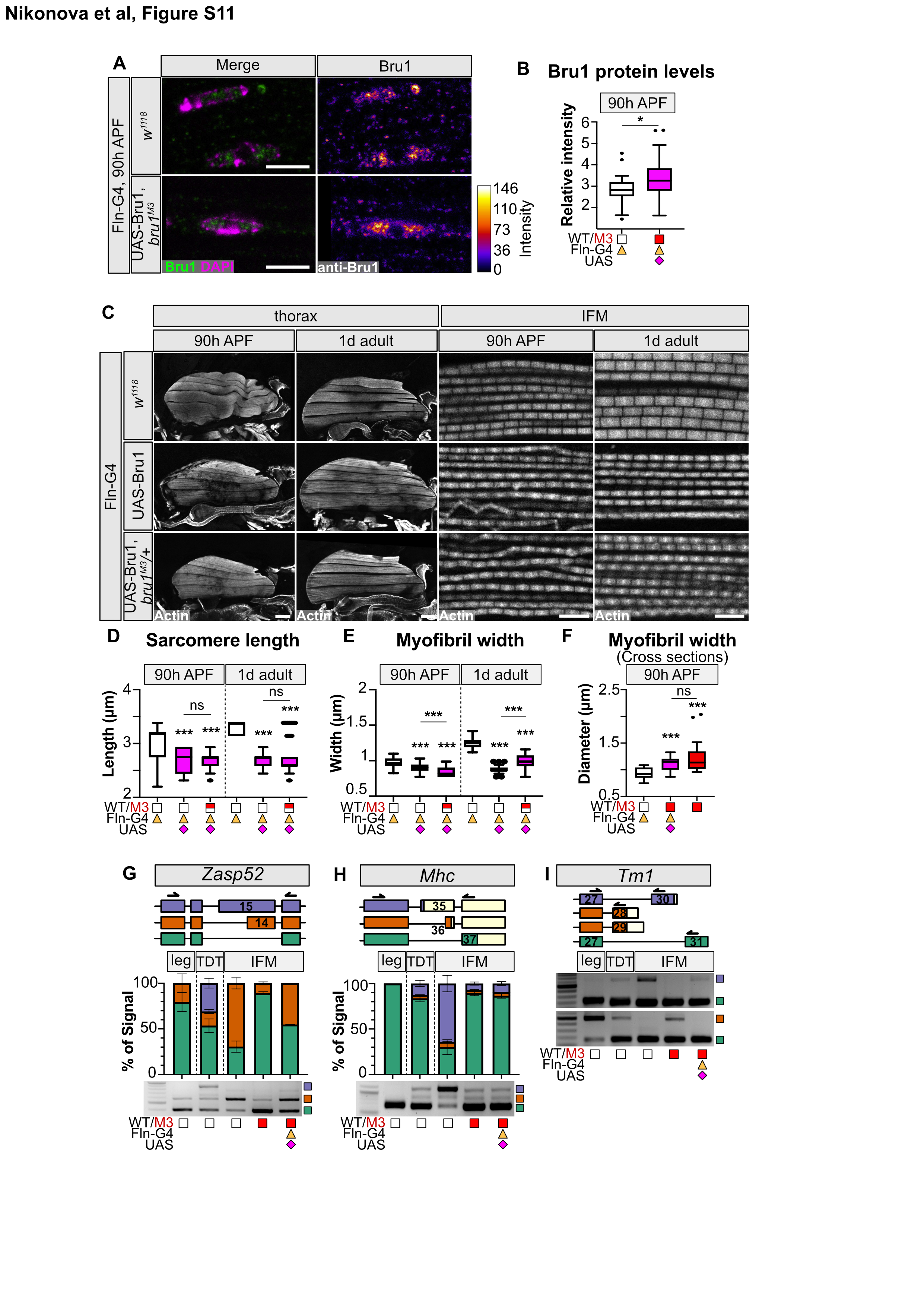

Supplement: S11 Fig — (A) Single-plane confocal images of IFM nuclei stained with rabbit anti-Bru1 in control and Fln-Gal4 rescue of bru1M3 at 90 h APF. Images were acquired using same settings and pseudo-colored based on intensity. Bru1, green; DAPI, magenta; scale bar = 5 μm. (B) Quantification of Bru1 relative signal intensity based on fluorescence levels in (A). Statistical significance determined by unpaired t test (*P < 0.05). (C) Confocal projections of hemithorax and single plane images of myofibrils at 90 h APF and in 1 d adult in control, Fln-Gal4 driving UAS-Bru1, and Fln-Gal4 driving UAS-Bru1 in a heterozygous mutant background (bru1M3/+). Phalloidin stained actin, gray; scale bar = 100 μm (hemithorax), or 5 μm (myofibrils). (D, E) Quantification of sarcomere length (D) and myofibril width (E) in (C). Genotypes marked by symbols as in S9 Fig. Boxplots are shown with Tukey whiskers, outlier data points marked as black dots. Significance determined for each time point by ANOVA and post hoc Tukey (ns, not significant; ***P < 0.001). (F) Quantification of myofibril width in (Fig 8R) at 90 h APF. Significant determined as in (D, E). (G, H) Semi-quantitative RT-PCR verification of alternative splice events in Zasp52 (G) and Mhc (H). Top: scheme of alternative isoforms with primer locations. Exon numbering in accordance with FB2021_05 annotation. Color coding of depicted isoforms consistent across top, middle, and bottom panels; 3′ UTR regions in light beige. Middle: Quantification of relative expression level of detectable events in control w1118 leg, jump (tergal depressor of the trochanter, TDT) and fibrillar IFM muscle, as well as bru1M3 and Fln-Gal4 rescue IFM. Error bars = SD. Bottom: representative RT-PCR gel image. (I) Semi-quantitative RT-PCR verification of alternative splice events in Tm1. Top: scheme of alternative isoforms. Bottom: representative RT-PCR gel image. Splice events in Tm1 were detected with distinct reverse primers, as isoforms do not share a common 3′-UTR. [file pbio.3002575.s011.tiff]

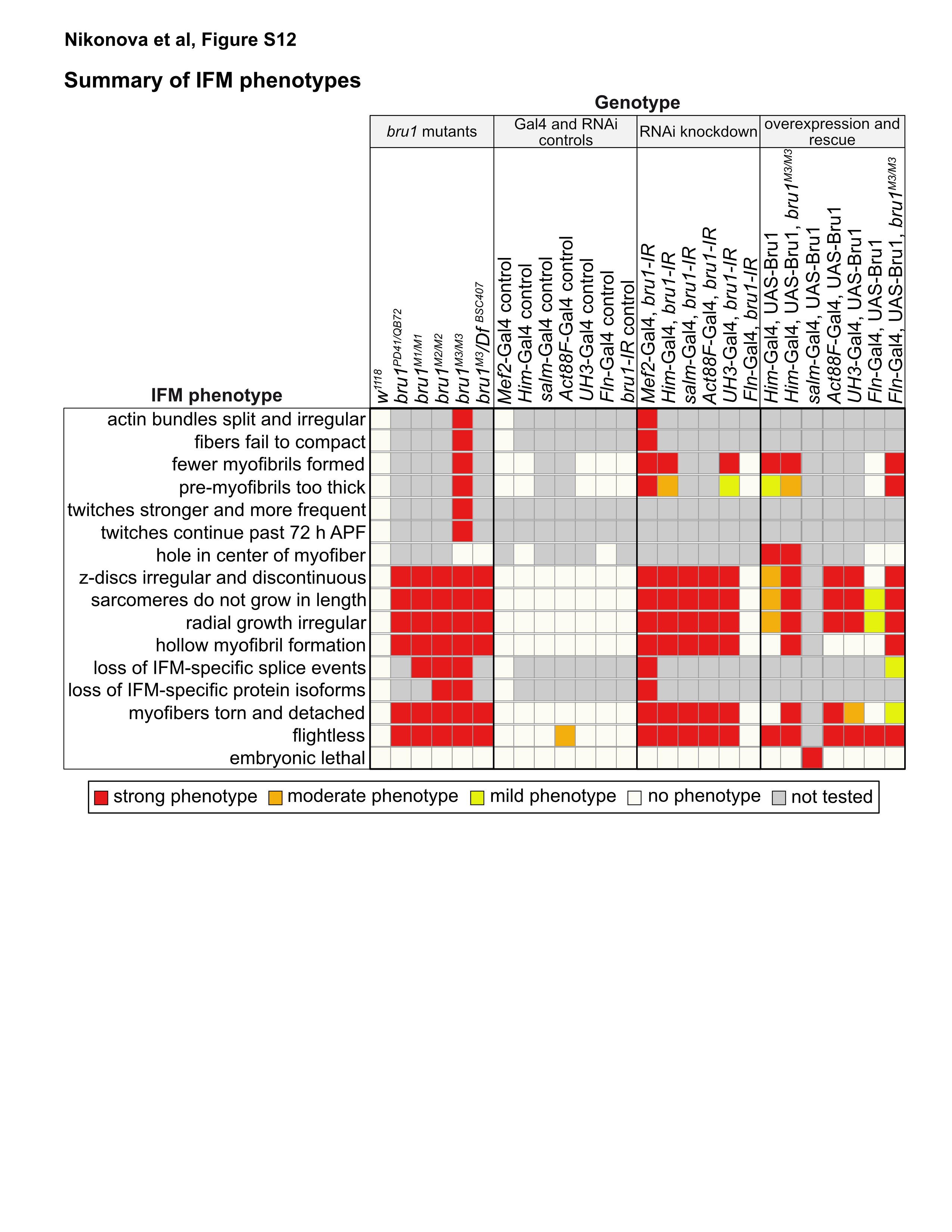

Supplement: S12 Fig — IFM phenotypes are summarized in table format for all genotypes used in this study, as well as bru1 mutant and RNAi phenotypes published previously [13,14,36,43,44]. Genotypes are as labeled. IFM phenotypes include early phenotypes in cytoskeletal rearrangement, myofiber compaction, and nascent myofibrils assayed in this study, as well as late pupal and adult phenotypes assayed in this and previous studies including flight ability, sarcomere, and myofibril structure; “loss of IFM-specific splice events” includes data from RT-PCR or mRNA-Seq, while “loss of IFM-specific protein isoforms” includes proteomics data and expression of GFP-tagged isoform reporters. Phenotypes were classified as strong (red), moderate (orange), mild (yellow), no phenotype (wild-type structure, white), or not tested/data not available (gray). Underlying data can be found throughout this manuscript and in references [13,14,36,43,44]. (TIFF) [file pbio.3002575.s012.tiff]
