# Supplementary material for: Bruno 1/CELF regulates splicing and cytoskeleton dynamics to ensure correct sarcomere assembly in Drosophila flight muscles
Source: PLoS Biol. 2024 Apr 29;22(4):e3002575. doi: 10.1371/journal.pbio.3002575 (PMC11081514; doi:10.1371/journal.pbio.3002575)
Supplement: S1 Raw Images — (PDF) [file pbio.3002575.s025.pdf]

Alternative splice events

in Fln Gal4 driven $bru1^{M3}$ rescue

Detected by RT-PCR

*Region in the red box in all gel images is included in the figure panel

Raw gel images supporting Figure 8 and S11 Figure

Strn-Mlck isoform R

Strn-Mlck 3'_F: GTTGGGTATCTACGATCTCACAGG
Strn-Mlck ex6_R: CACGAAGGACATTACCCAATCGG

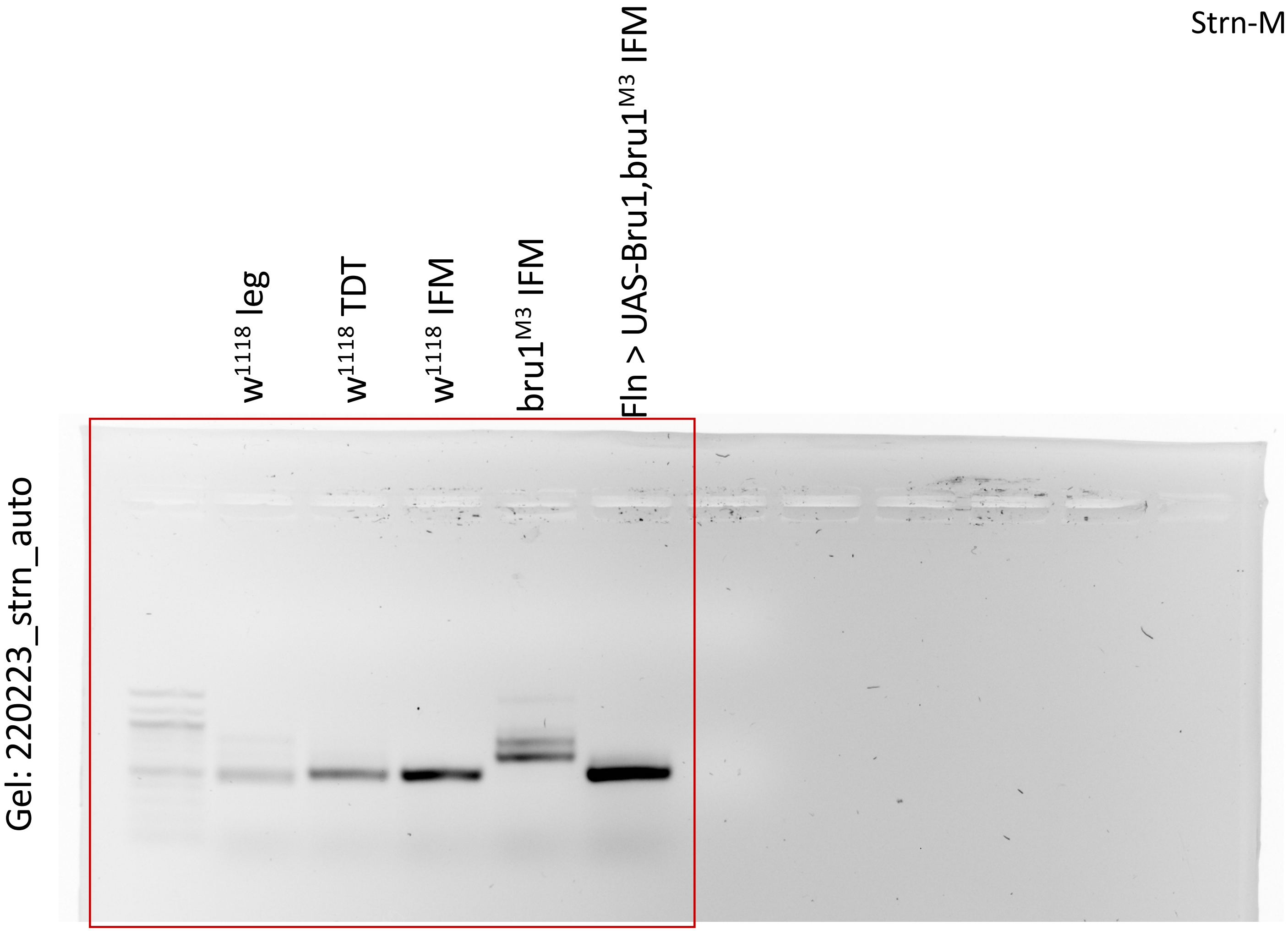

3'UTR– ex6: 466 bp

Raw gel image supporting Figure 8V

sls

Ex 9_F: CGCGCAGTATGTGCAAAAT
Ex 11_R: AAACCGTTCCACGAAAAGTG
From Oas et al., 2014

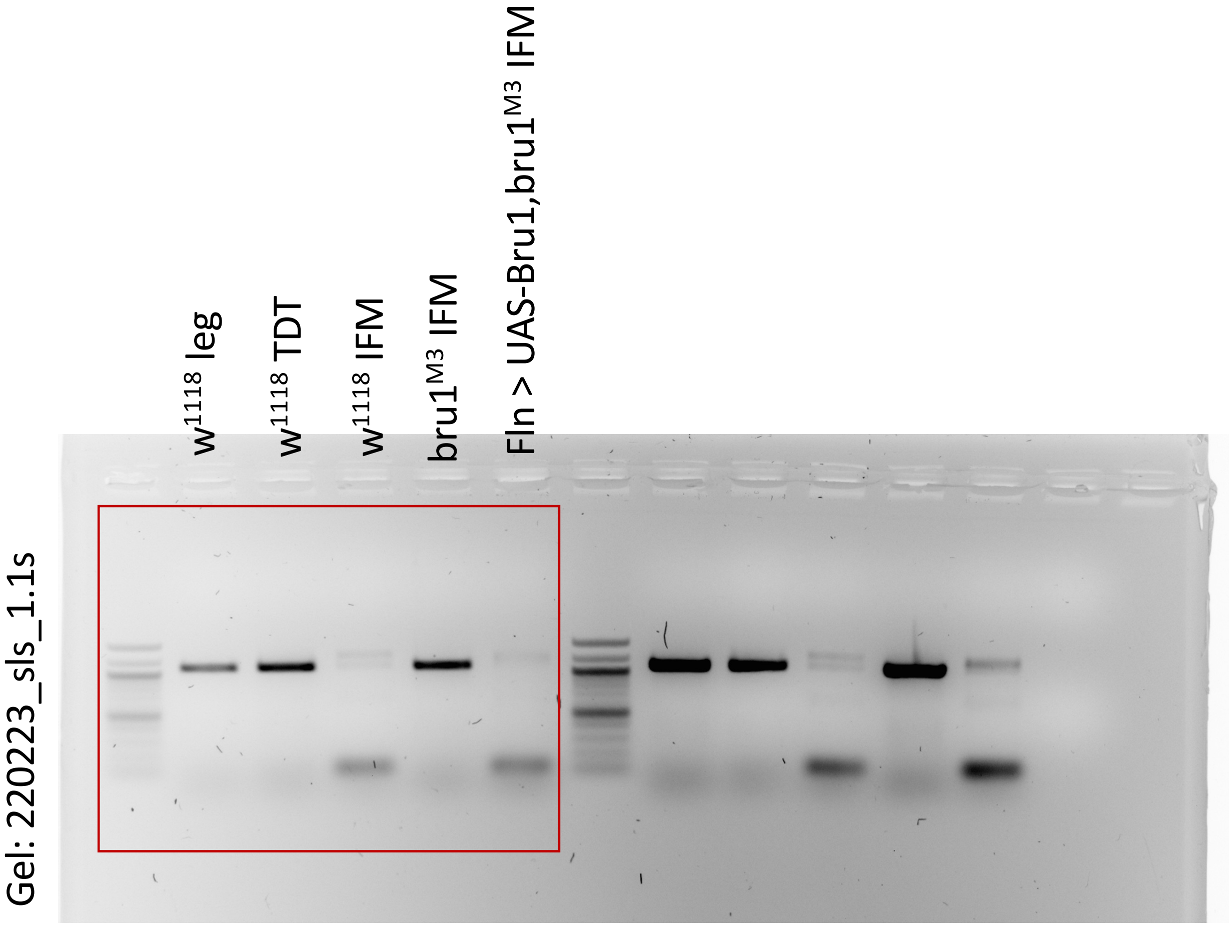

Tubular iso: 1075 bp
IFM iso: 106 bp

Raw gel image supporting Figure 8W

wupA

wupA ex5_F: CGCTGAGTTCAACTTCCGCAACC
wupA ex3_R: ATTGTTTAGGGCGGGAGTCACGG

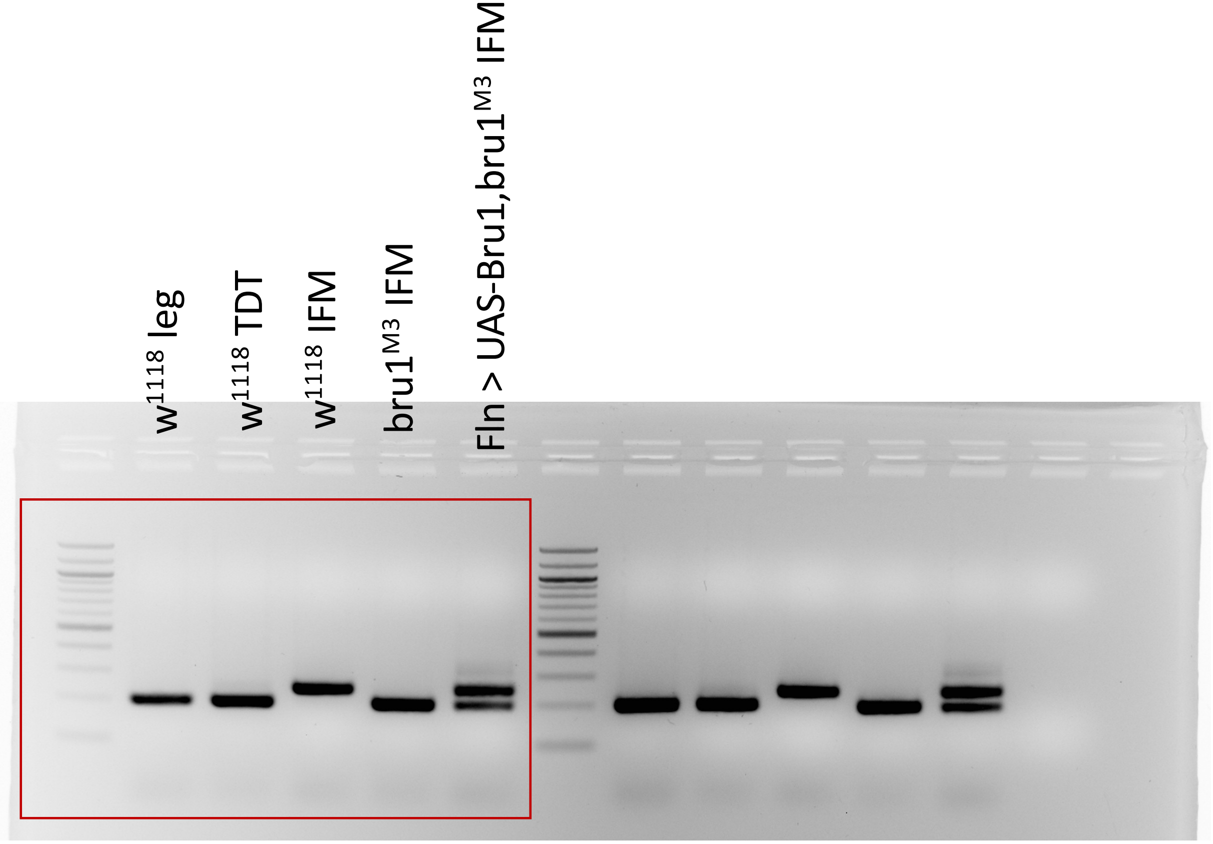

IFM isofom: 244 bp
Tubular isoform: 192 bp

Raw gel image supporting Figure 8X

Zasp52

Ex17_F: ATCGCTTCCGACGTTCTGAAG
Ex13_R: GTCGCAGTAGAGCTTGTTGTTG

From Oas et al., 2014

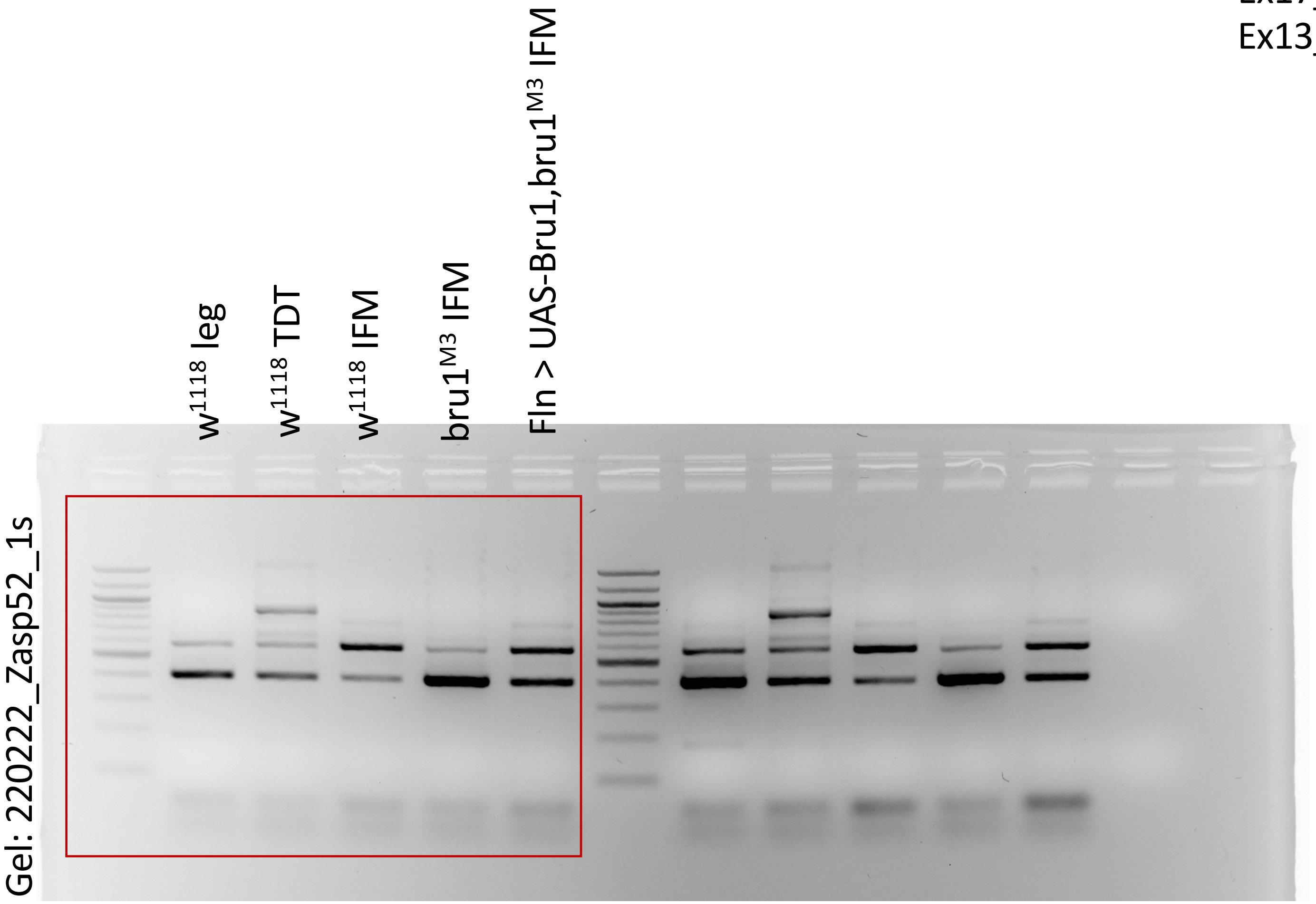

TDT iso: 800 bp
IFM iso: 575 bp
Tubular iso: 398 bp

Raw gel image supporting S11G Figure

Mhc

Ex34_F: GACGAACTCCTGAACGAAGC
Ex37_R: TCAGGAGCAAGGTCGAATCT
From Orfanos et al., 2012

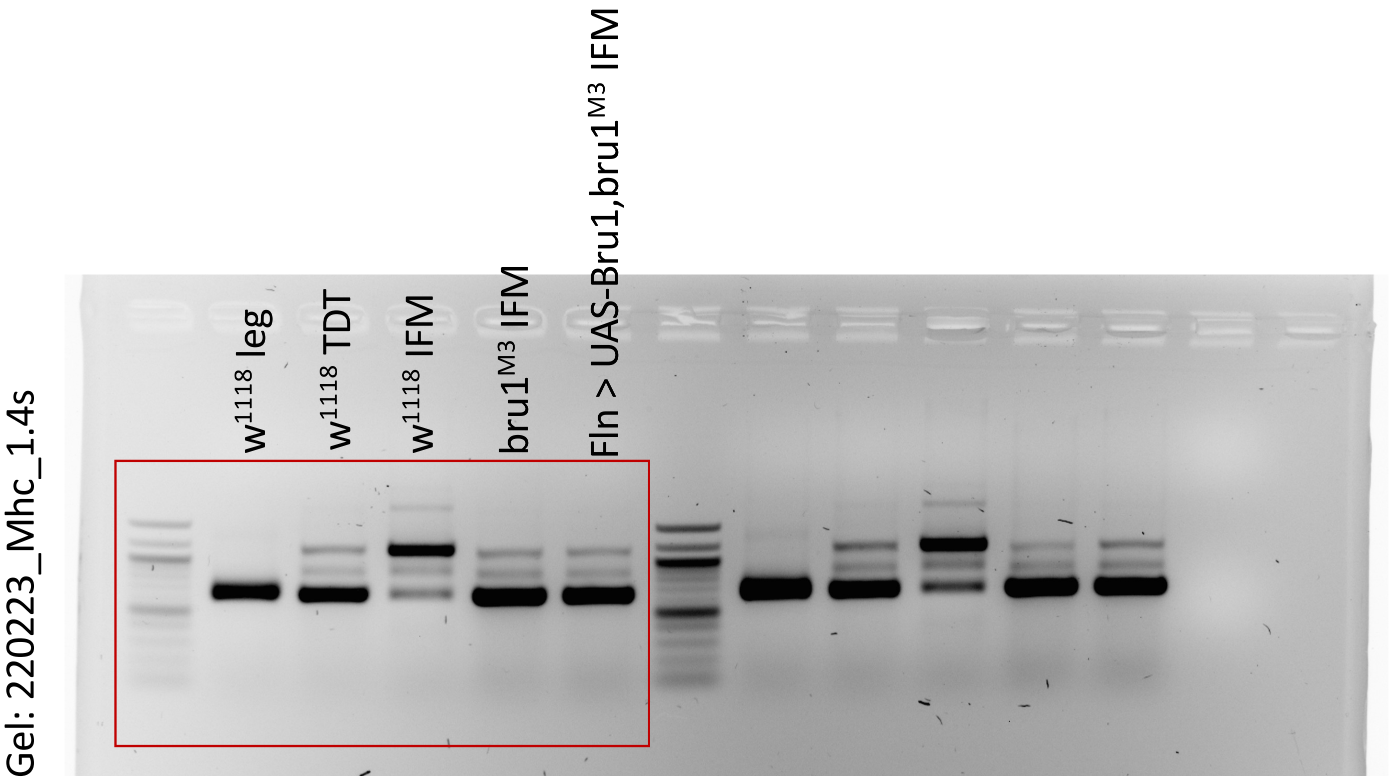

IFM isofom: 1158 bp
Tubular isoform: 656 bp

Raw gel image supporting S11H Figure

Tm1 Ex27-30

Ex27_F: TGAGTTCGCTGAACGCTCCG

Ex30_R: AGGTGCTGGTGCTCCTTCTGCC

Ex31_R: AGTCGGCGGCTTAGGGTTGCG

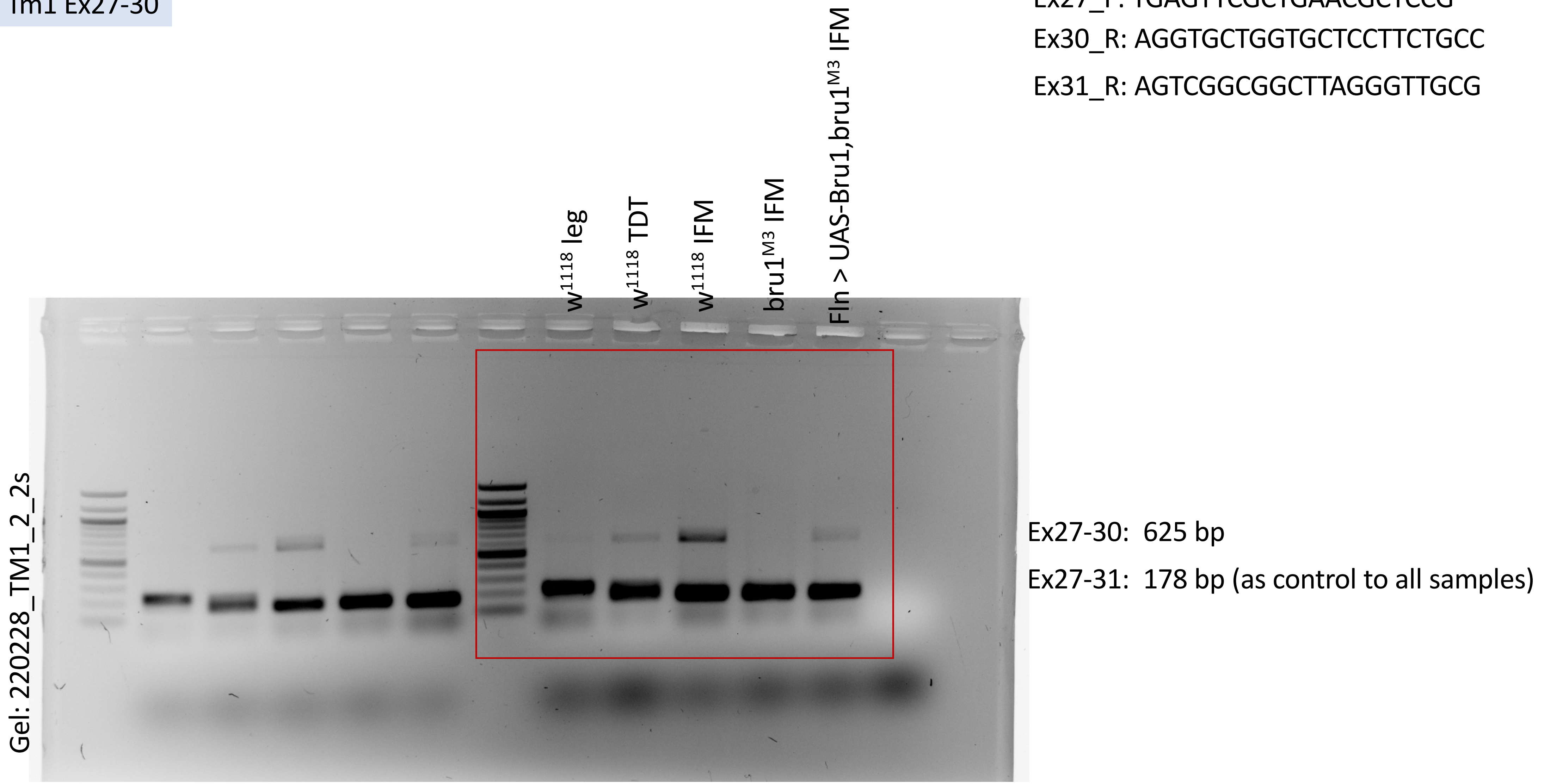

Raw gel image supporting S11I Figure

Tm1 Ex 27-28/29

Ex27 F: TGAGTTCGCTGAACGCTCCG

Ex28/29 R: AATCGTGAATTGGAATGCGC

Ex31 R: AGTCGGCGGCTTAGGGTTGCG

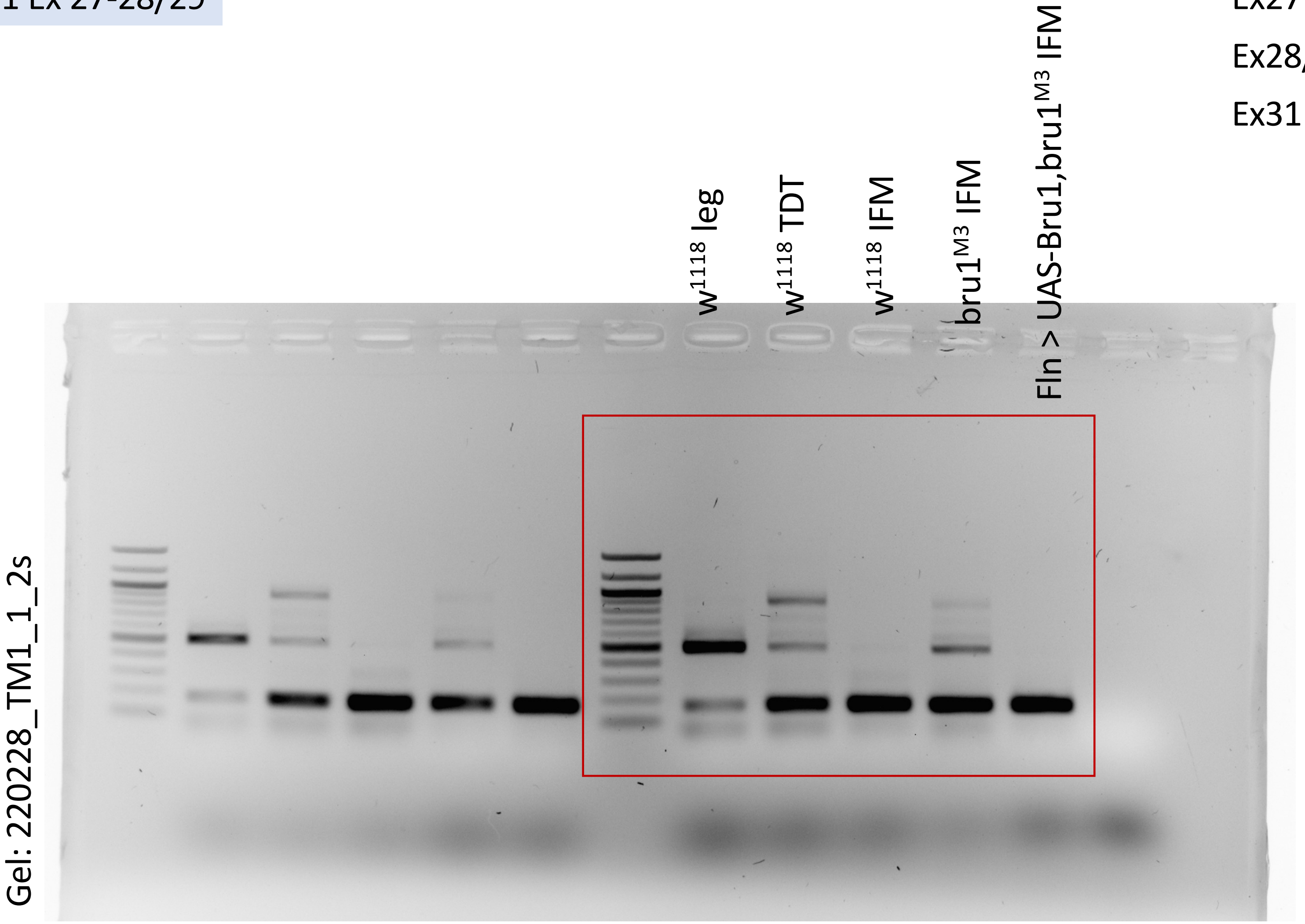

Ex27-28/29: 510 bp

Ex27-31: 178 bp (as control to all samples)

Raw gel image supporting S11I Figure

Verification of bru1M3 CRISPR allele

By genomic PCR and RT-PCR

Raw gel images supporting S1 Figure

Presence of native bru1 sequence

Ex12_F: TTTGAAGTCCACGGAGCC

Ex14_R: AGCCGTGTTGGTCAGTTGGG

Whole fly Genomic DNA

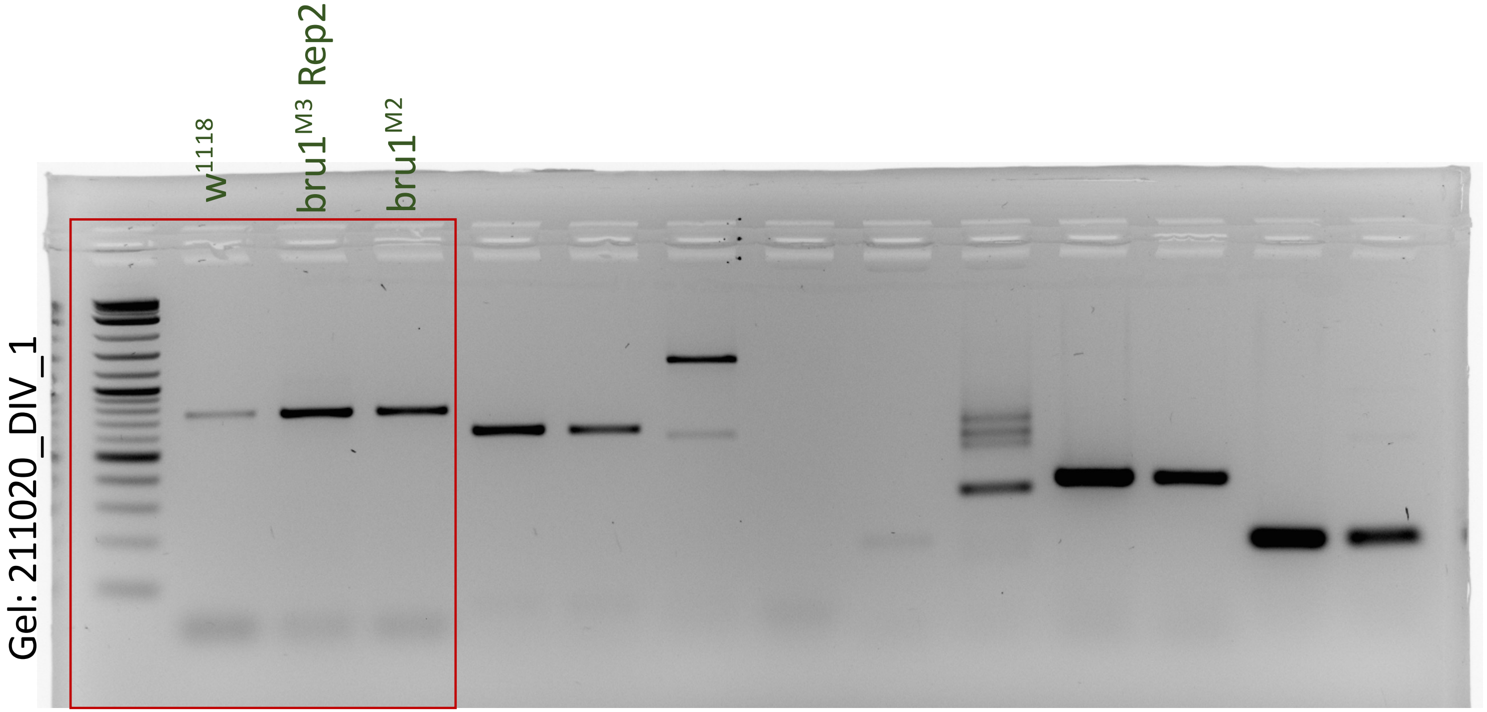

Ex 12- ex14: 775 bp

Note: bru1^{M3} Rep2 is a biological replicate

Raw gel image S1B Figure

Presence of native bru1 sequence

Ex18_F: TGCTTCGGGTTTGTCTCCTTCGAC

Ex19_R: CCTCTACTCGATGTGTCCGTTTAACCTT

Whole fly Genomic DNA

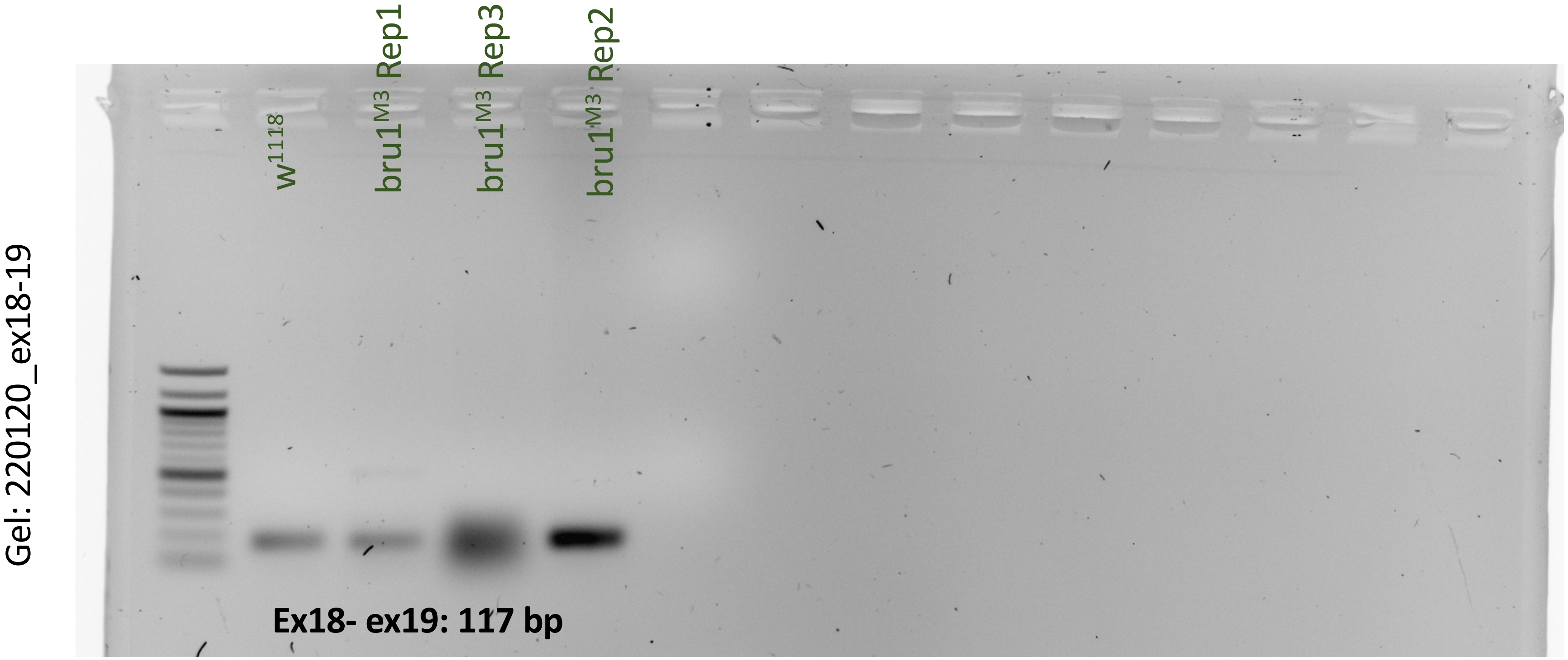

Note: $bru1^{M3}$ Rep1, 2 and 3 are biological replicates

Raw gel image S1B Figure

dsRed cassette

bru1 intron Left_F: cgttcgtgcccttgagagg

SV40 terminator_R: gactagttgatcataatcagcca

Whole fly Genomic DNA

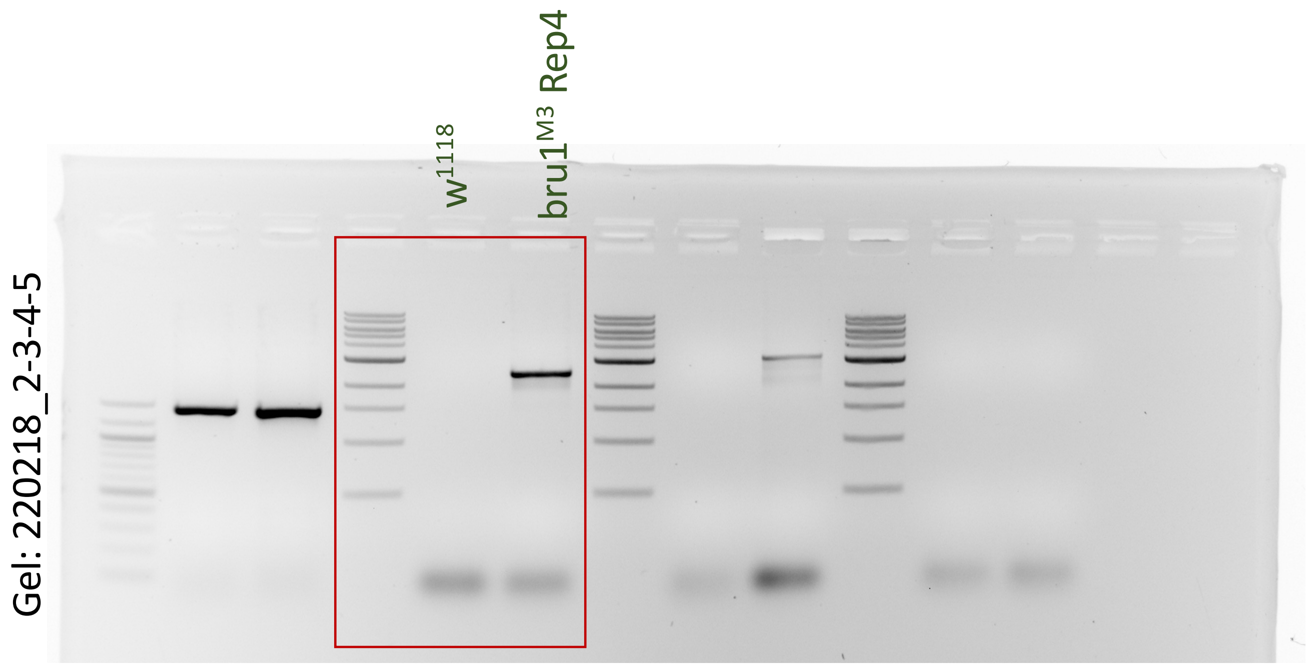

Intron L – dsRed(SV40): 2422 bp

Note: bru1^{M3} Rep4 is a biological replicate

Raw gel image S1B Figure

dsRed cassette

dsRed_F: ccacaaggccctgaagctga

bru1 intron Right_R: TATGTTAGGCGGGTTGAAGC

Whole fly Genomic DNA

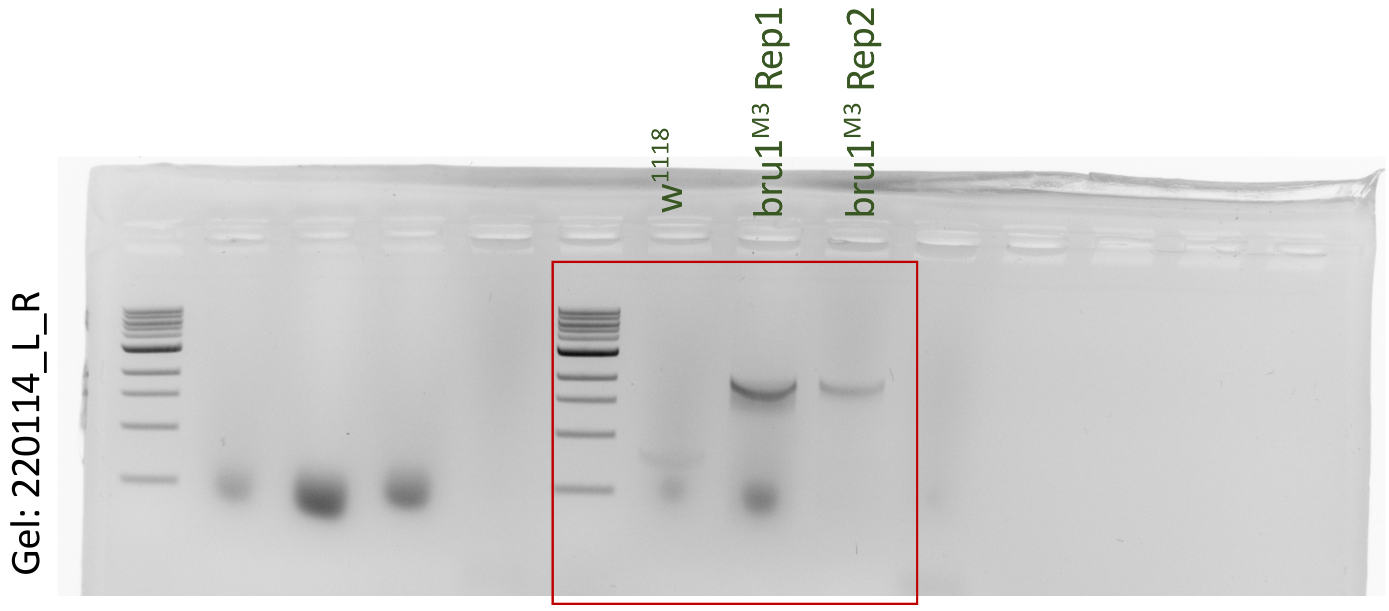

dsRed – intron R: 1977 bp

Note: $bru1^{M3}$ Rep1 and 2 are biological replicates

Raw gel image S1B Figure

dsRed cassette localization

bru1M3 homology arm L1_F: gccaactcatacatcaaggtg

Ex18_R: AGTAGGGCTTCGAGTCCTTG

Whole fly Genomic DNA

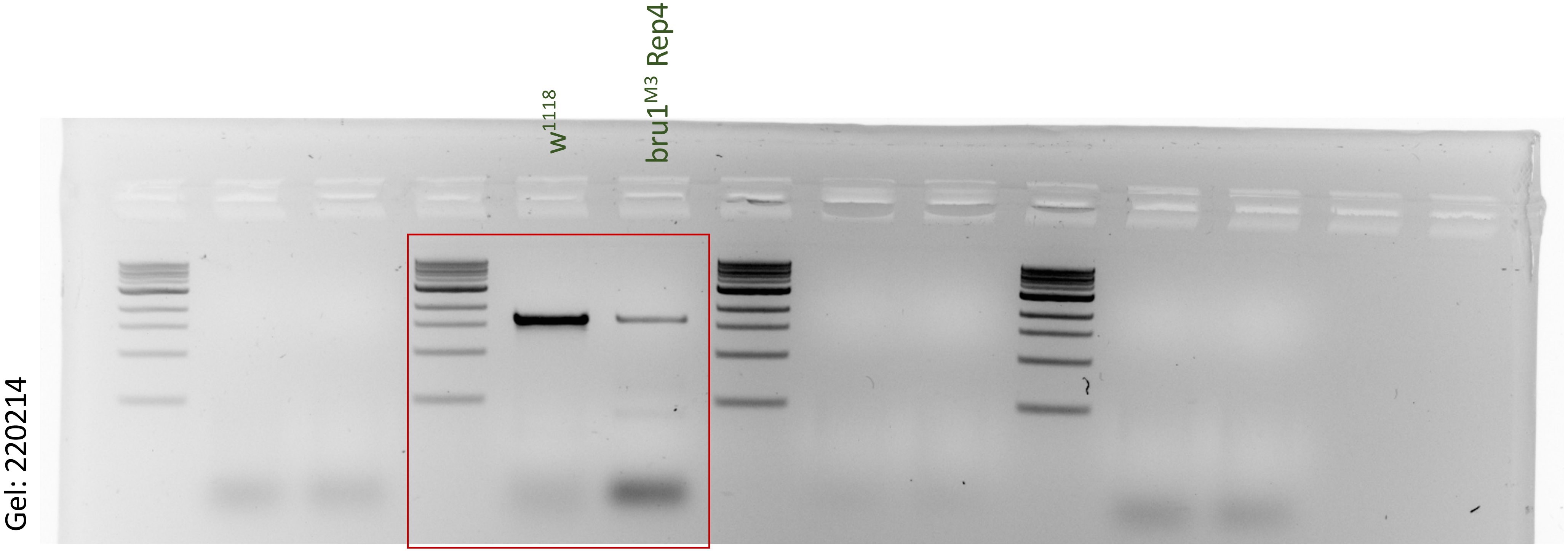

HA-L1 – ex18: 1495 bp

Raw gel image S1B Figure

dsRed cassette localization

bru1M3 homology arm L2_F: ctatttataagtggtacgcc

Ex18_R: AGTAGGGCTTCGAGTCCTTG

Whole fly Genomic DNA

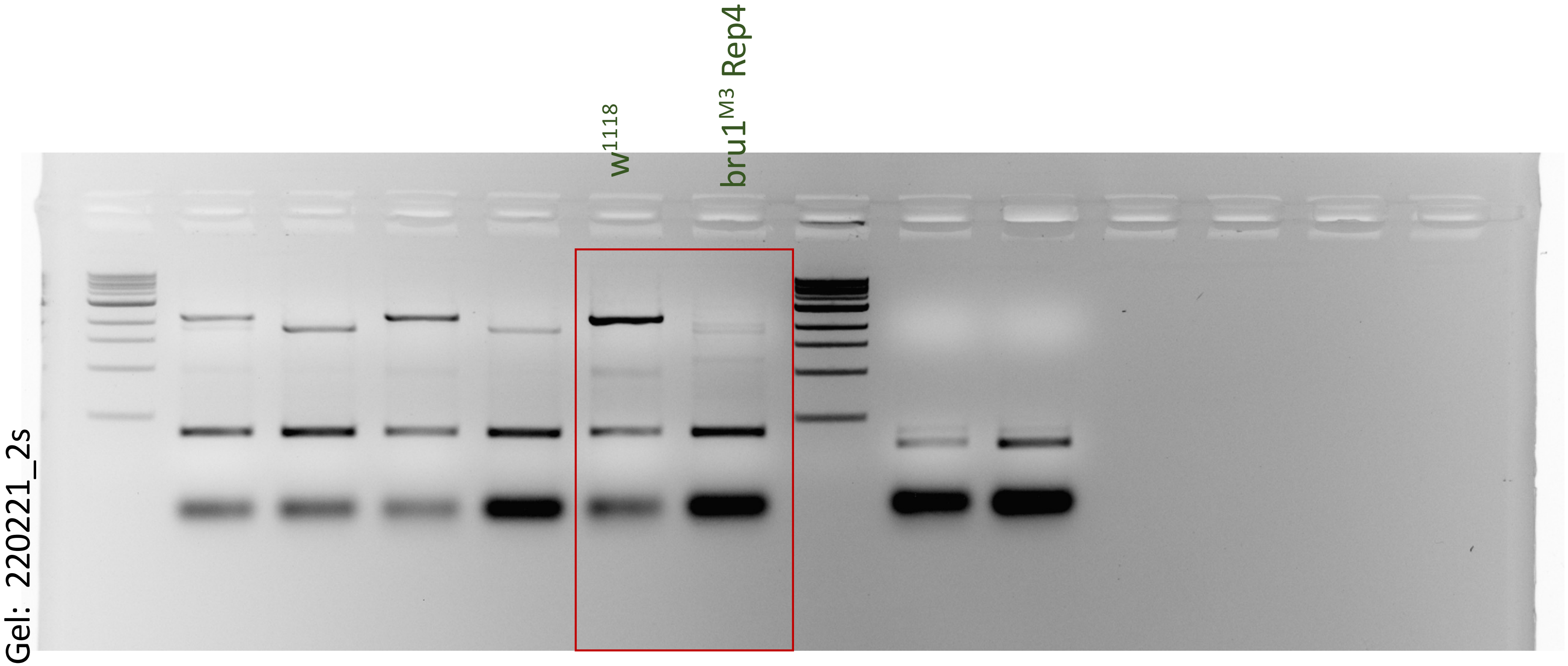

HA-L 2 - ex18: 2022 bp

Raw gel image S1B Figure

Presence of native bru1 transcripts

Ex12_F: TTTGAAGTCCACGGAGCC
Ex14_R: AGCCGTGTTGGTCAGTTGGG

RT-PCR

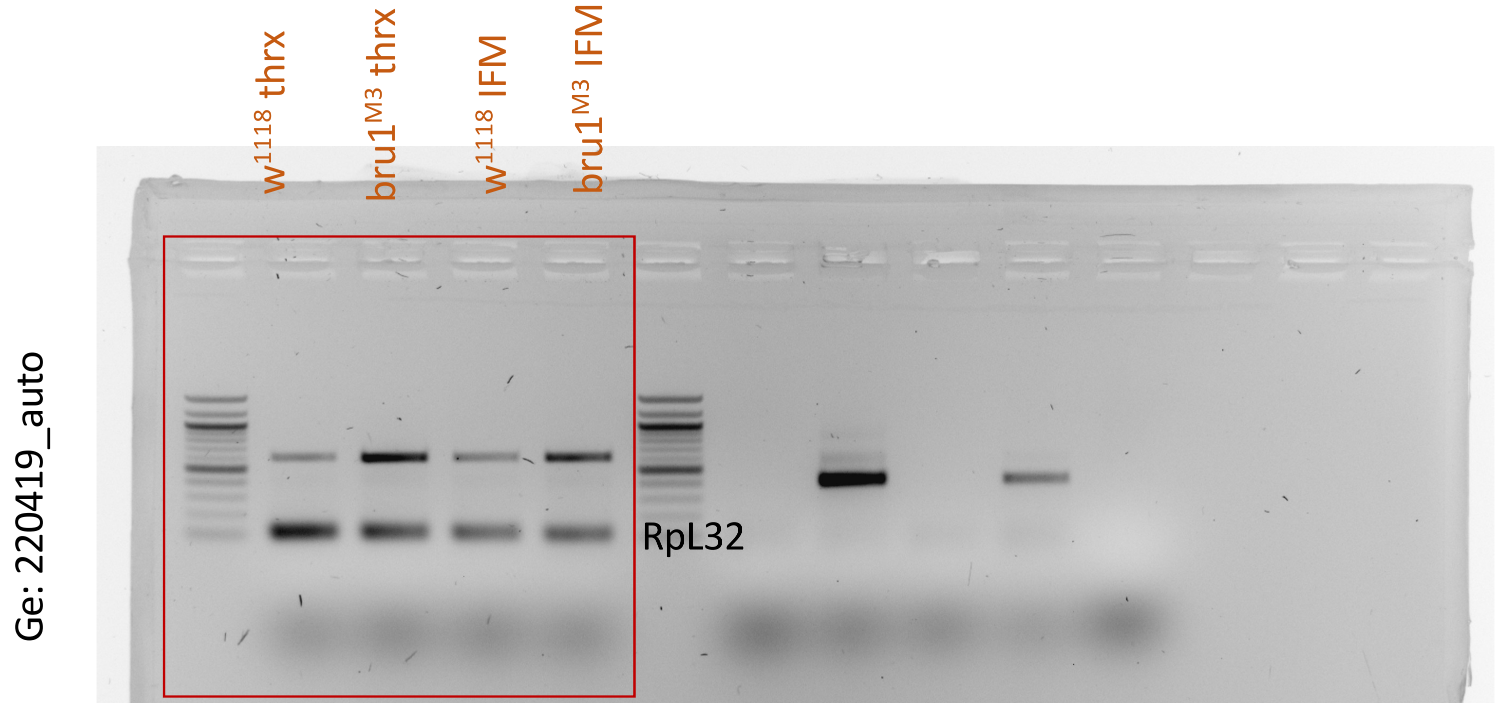

Ex12 - ex14: 615 bp

Raw gel image S1C Figure

Presence of native bru1 transcripts

Ex14_F: CCAGAATCTAGCGGCCATT
Ex18_R: AGTAGGGCTTCGAGTCCTTG

RT-PCR

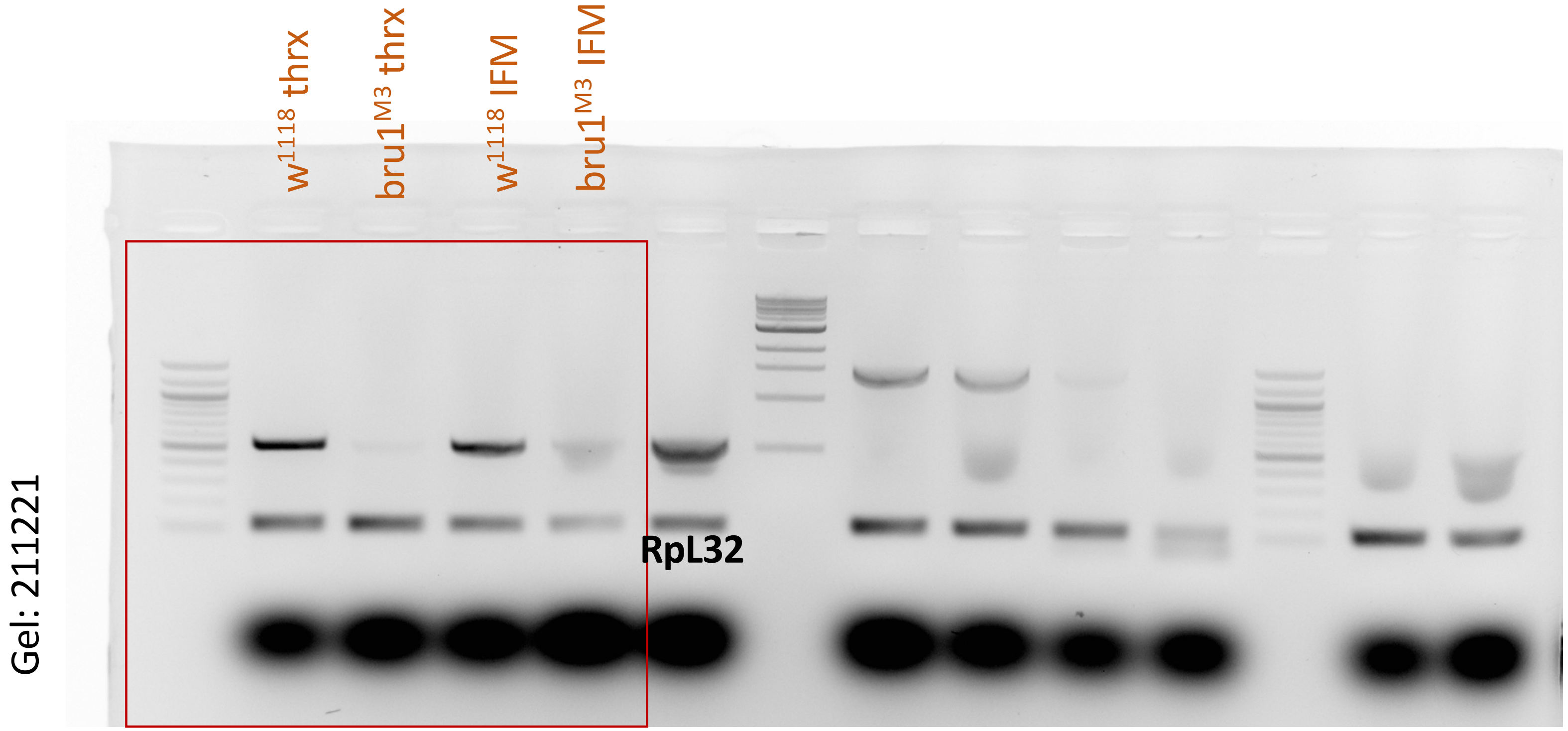

Ex14 - ex18: 536 bp

Raw gel image S1C Figure

Presence of native bru1 transcripts

Ex18_F: TGCTTCGGGTTTGTCTCCTTCGAC
Ex21_R: TCAAGATGTGCAACGACAAATAGCC

RT-PCR

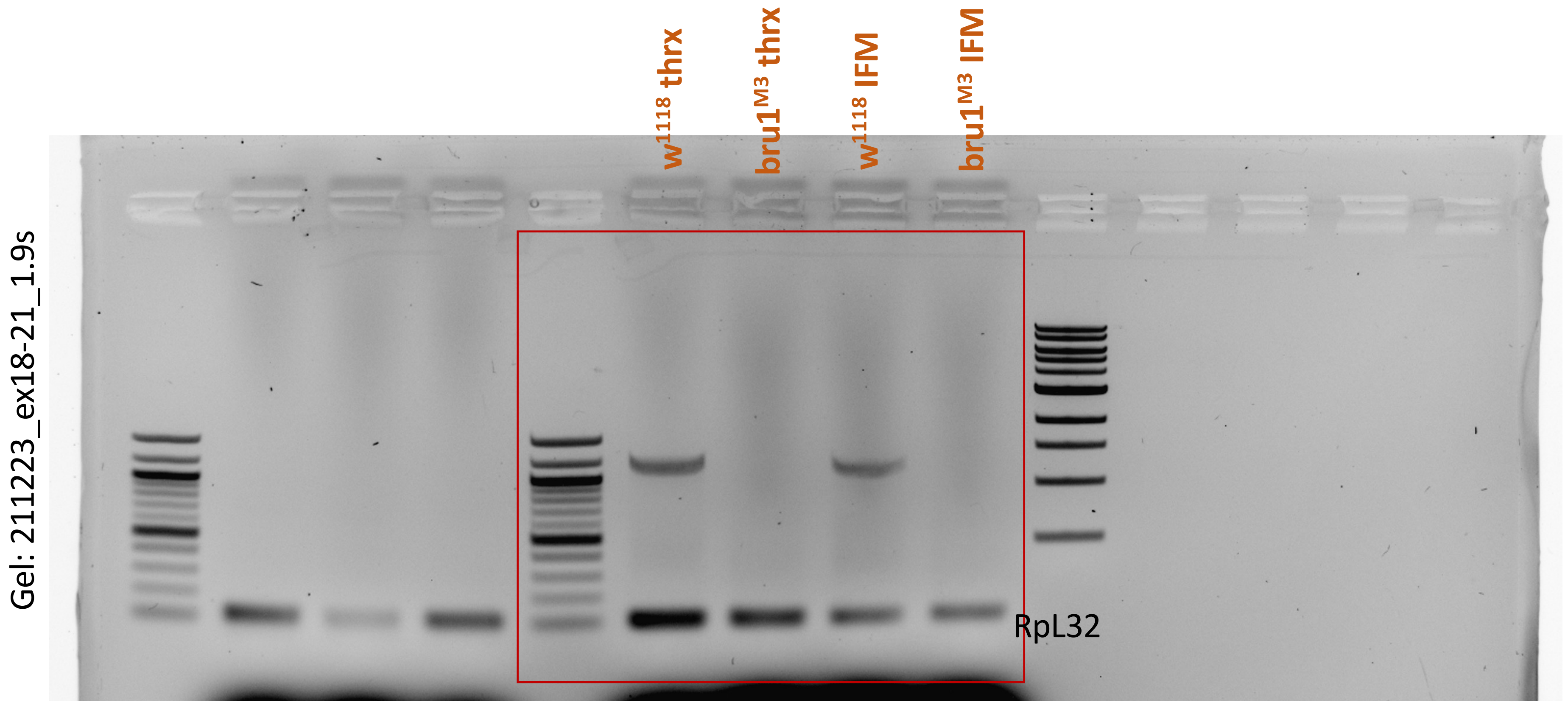

Ex18 – ex21: 1263 bp

Raw gel image S1C Figure

Splicing into dsRed cassette

Ex14_F: CCAGAATCTAGCGGCCATT

RT-PCR

SV40 terminator_R: gactagttgatcataatcagcca

dsRed: MS286

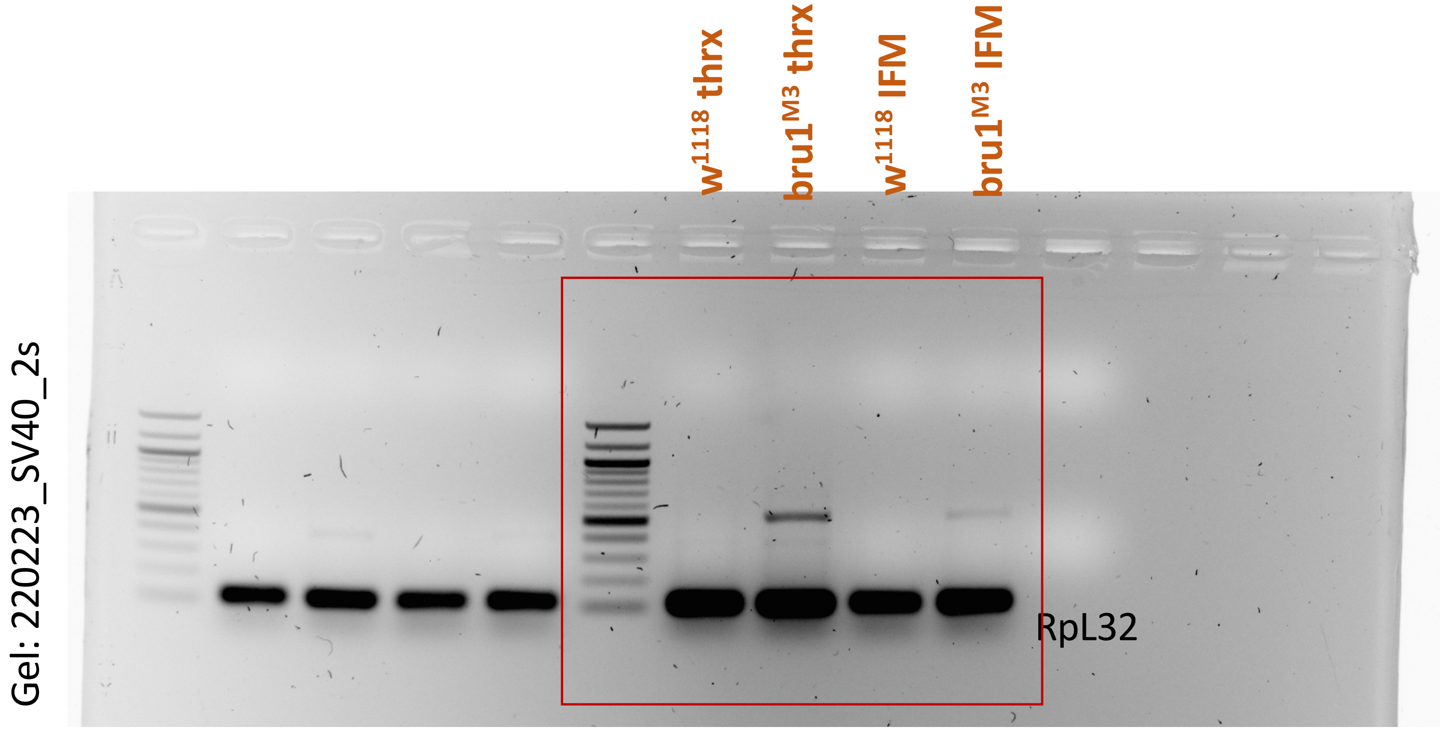

Ex14- SV40 terminator: 640 bp

Raw gel image S1C Figure

Splicing into dsRed cassette

Ex17_F: ATCGGCATATATGTCAACAGC

RT-PCR

SV40 terminator_R: gactagttgatcataatcagcca

Ge: 220419_auto

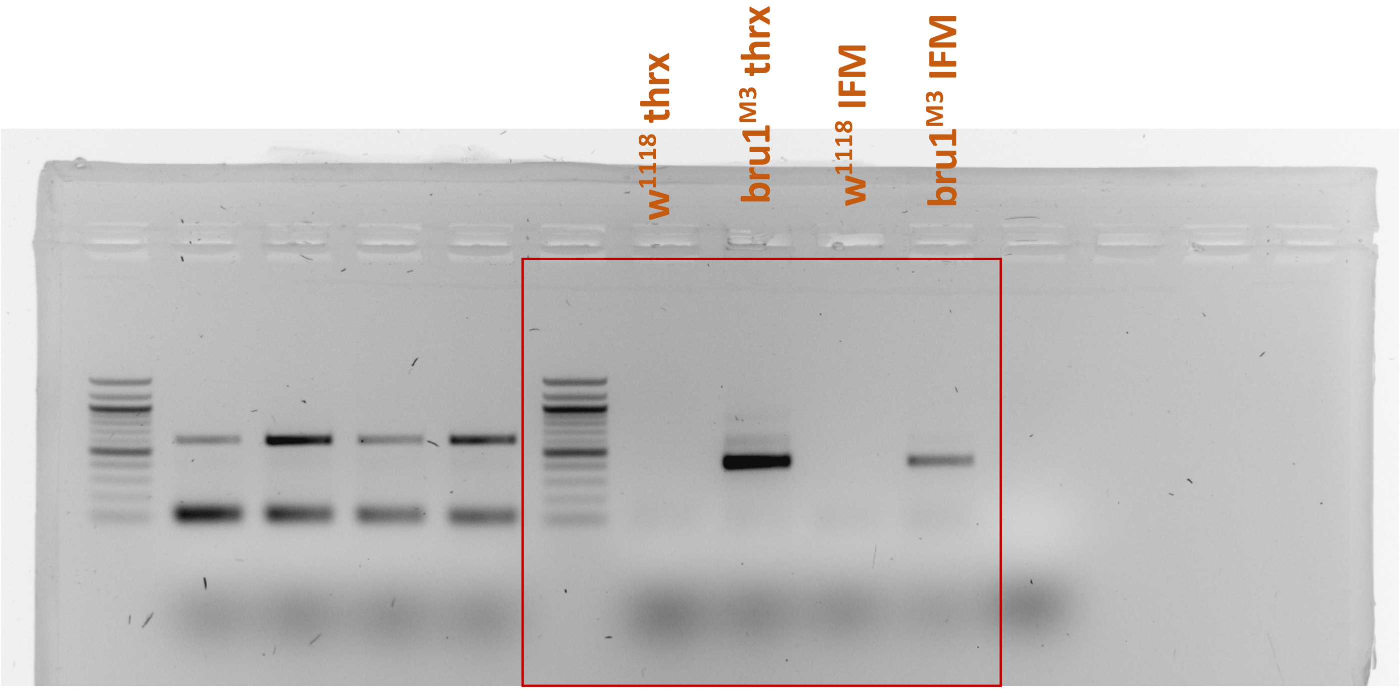

Ex17- SV40 terminator: 497 bp

Raw gel image S1C Figure

Alternative splice events

in bru1^{M3}

Detected by RT-PCR

Raw gel images supporting S2 Figure

Strn-Mlck isoform R

Strn-Mlck 3'_F: GTTGGGTATCTACGATCTCACAGG
Strn-Mlck ex6_R: CACGAAGGACATTACCCAATCGG

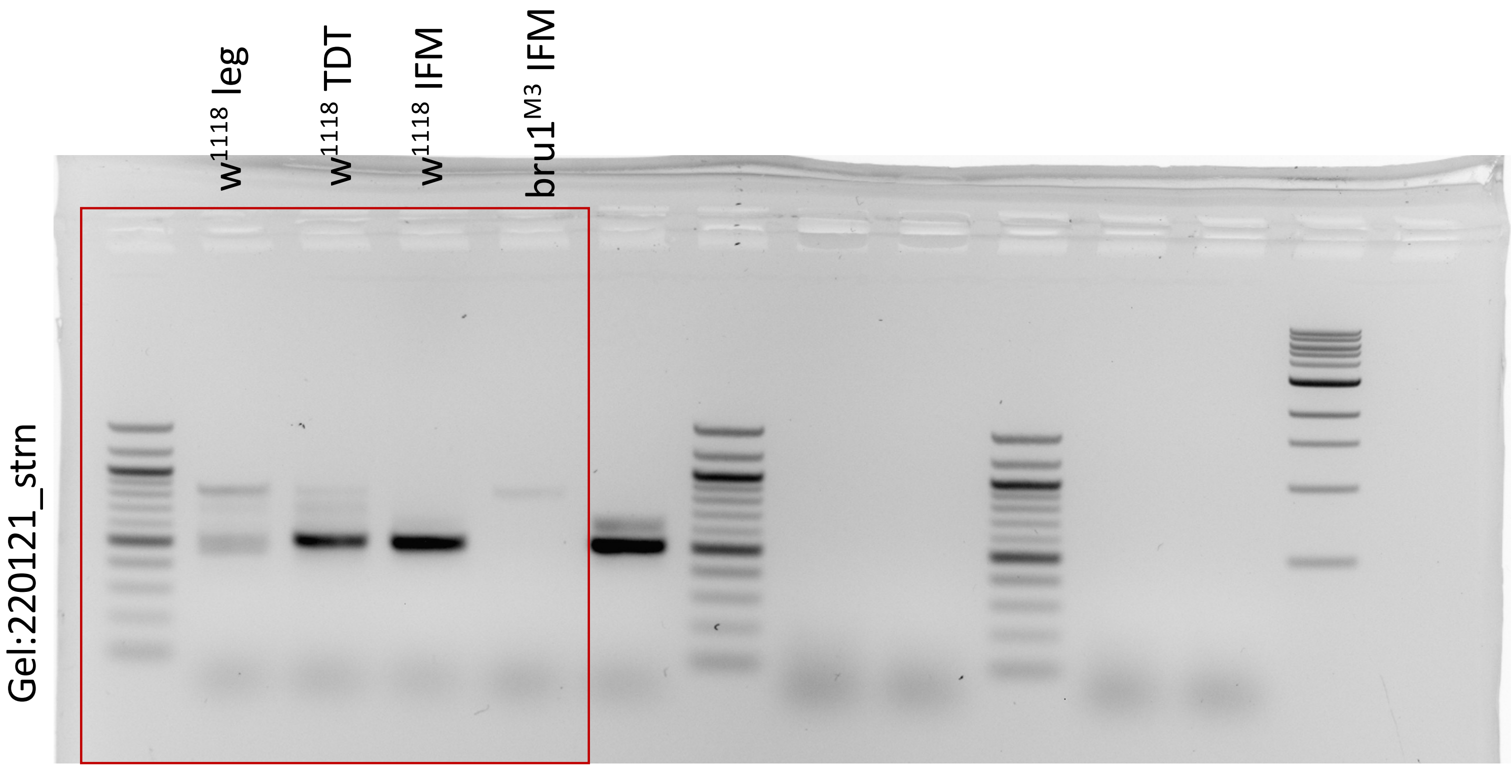

3'UTR– ex6: 466 bp

Raw gel image S2C Figure

wupA

wupA ex5_F: CGCTGAGTTCAACTTCCGCAACC
wupA ex3_R: ATTGTTTAGGGCGGGAGTCACGG

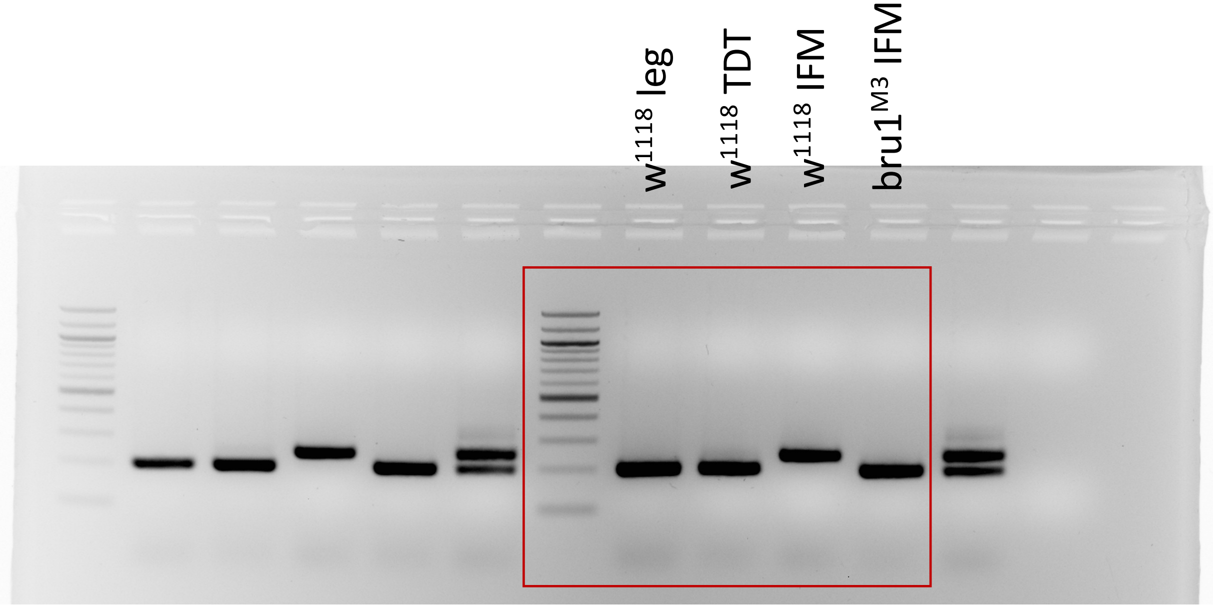

IFM isofom: 244 bp
Tubular isoform: 192 bp

Raw gel image S2D Figure

Mhc

Ex34_F: GACGAACTCCTGAACGAAGC
Ex37_R: TCAGGAGCAAGGTCGAATCT
From Orfanos et al., 2012

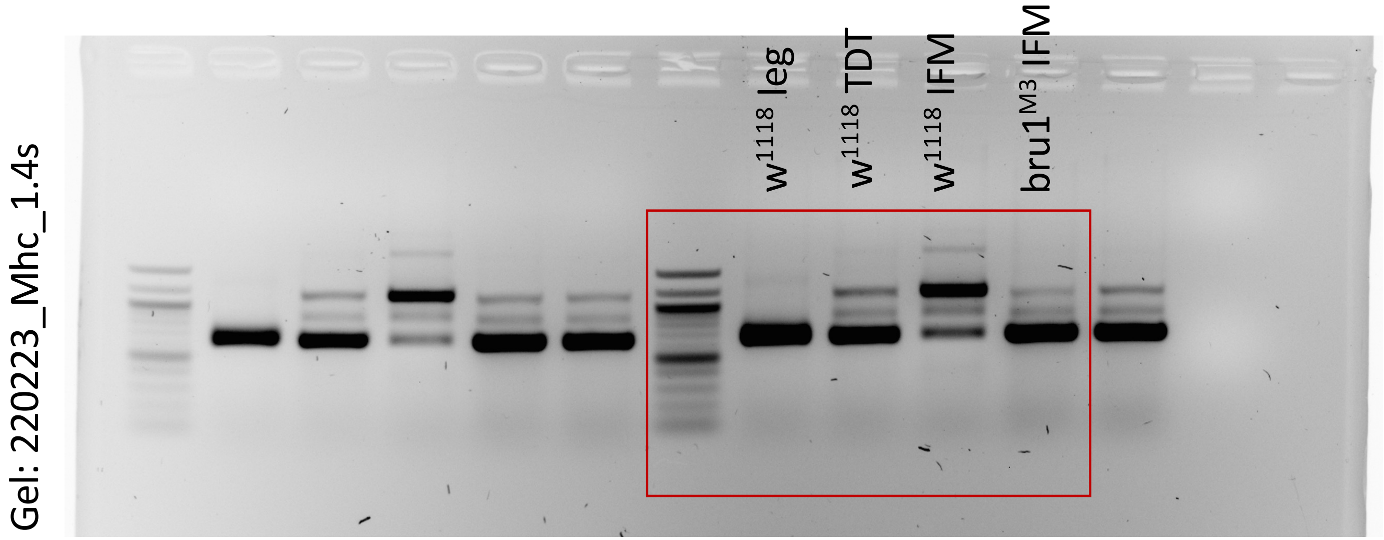

IFM isofom: 1158 bp
Tubular isoform: 656 bp

Raw gel image S2E Figure

Zasp52

Ex17_F: ATCGCTTCCGACGTTCTGAAG
Ex13_R: GTCGCAGTAGAGCTTGTTGTTG

From Oas et al., 2014

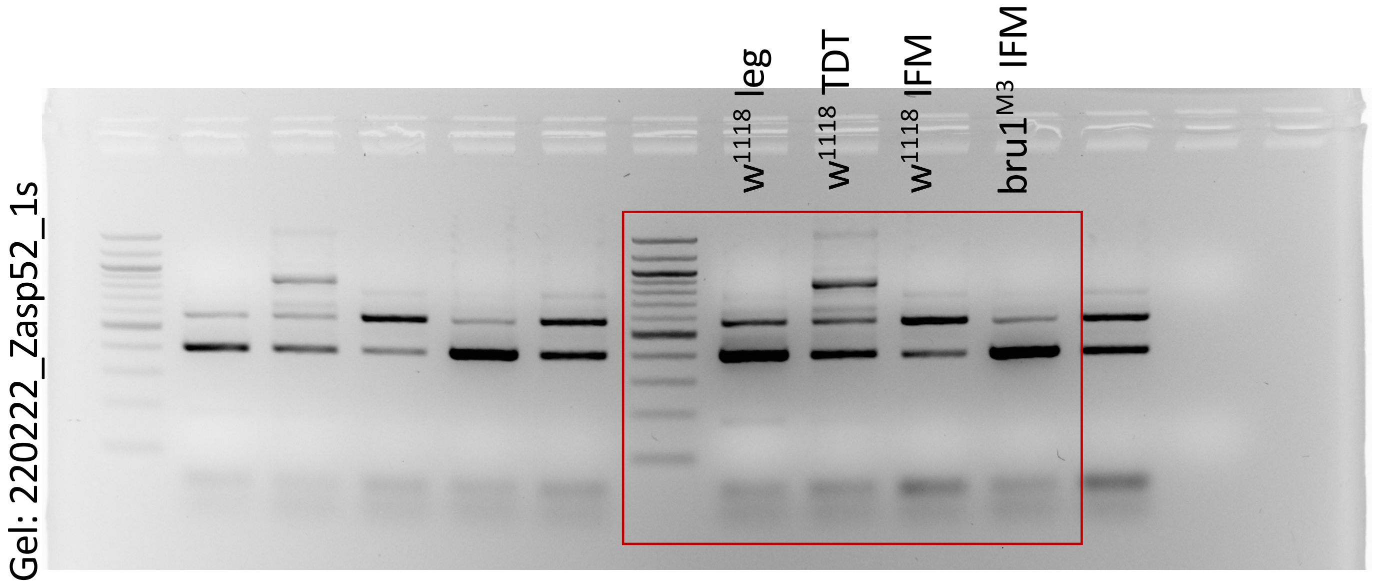

TDT iso: 800 bp
IFM iso: 575 bp
Tubular iso: 398 bp

Raw gel image S2O Figure

Tm1 Ex27-30

Ex27_F: TGAGTTCGCTGAACGCTCCG

Ex30_R: AGGTGCTGGTGCTCCTTCTGCC

Ex31_R: AGTCGGCGGCTTAGGGTTGCG

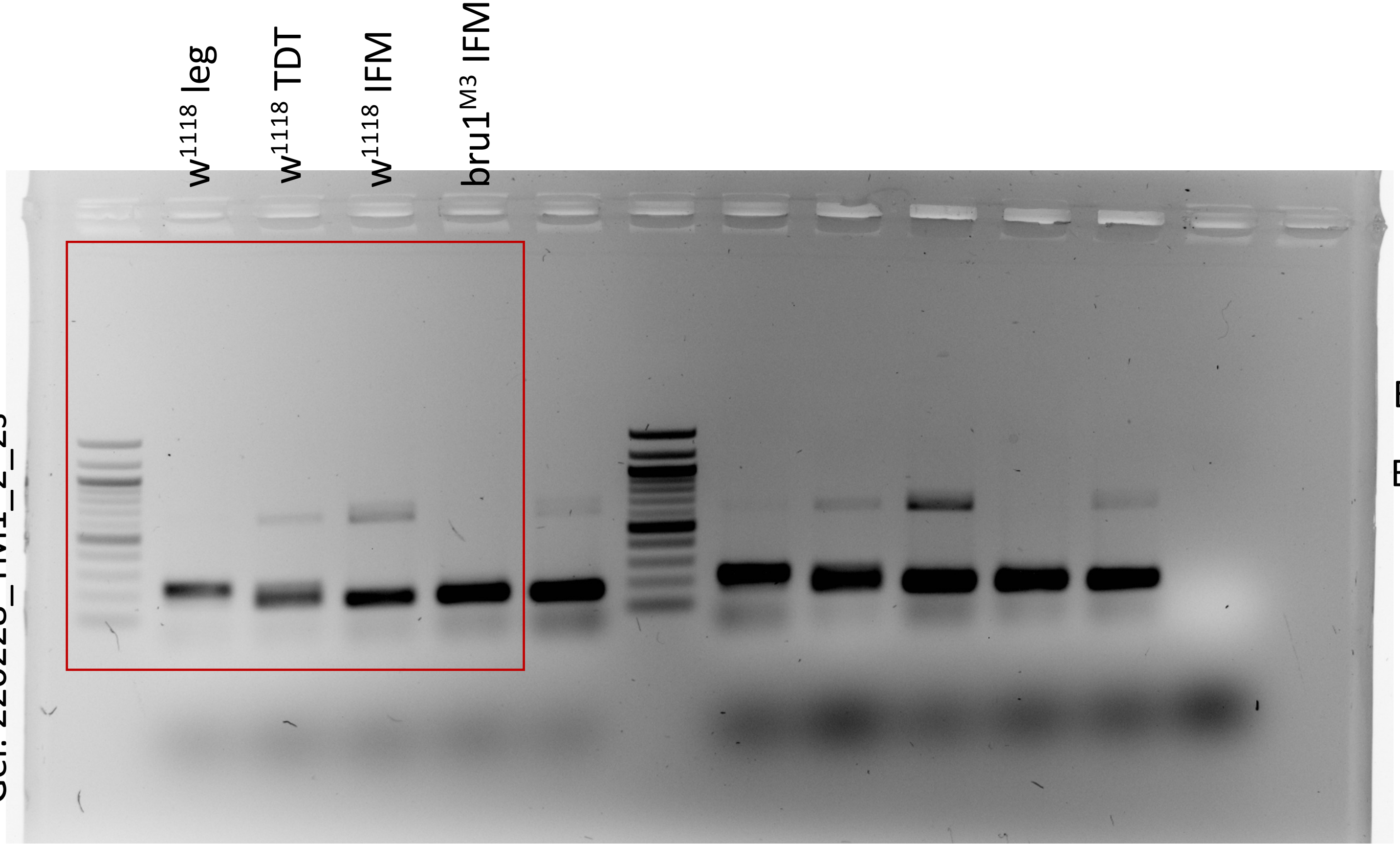

Ex27-30: 625 bp

Ex27-31: 178 bp (as control to all samples)

Raw gel image S2P Figure

Tm1 Ex 27-28/29

Ex27 F: TGAGTTCGCTGAACGCTCCG

Ex28/29 R: AATCGTGAATTGGAATGCGC

Ex31 R: AGTCGGCGGCTTAGGGTTGCG

w^{1118} leg

w^{1118} TDT

w^{1118} IFM

$bru1^{M3}$ IFM

Gel: 220228_TM1_1_2s

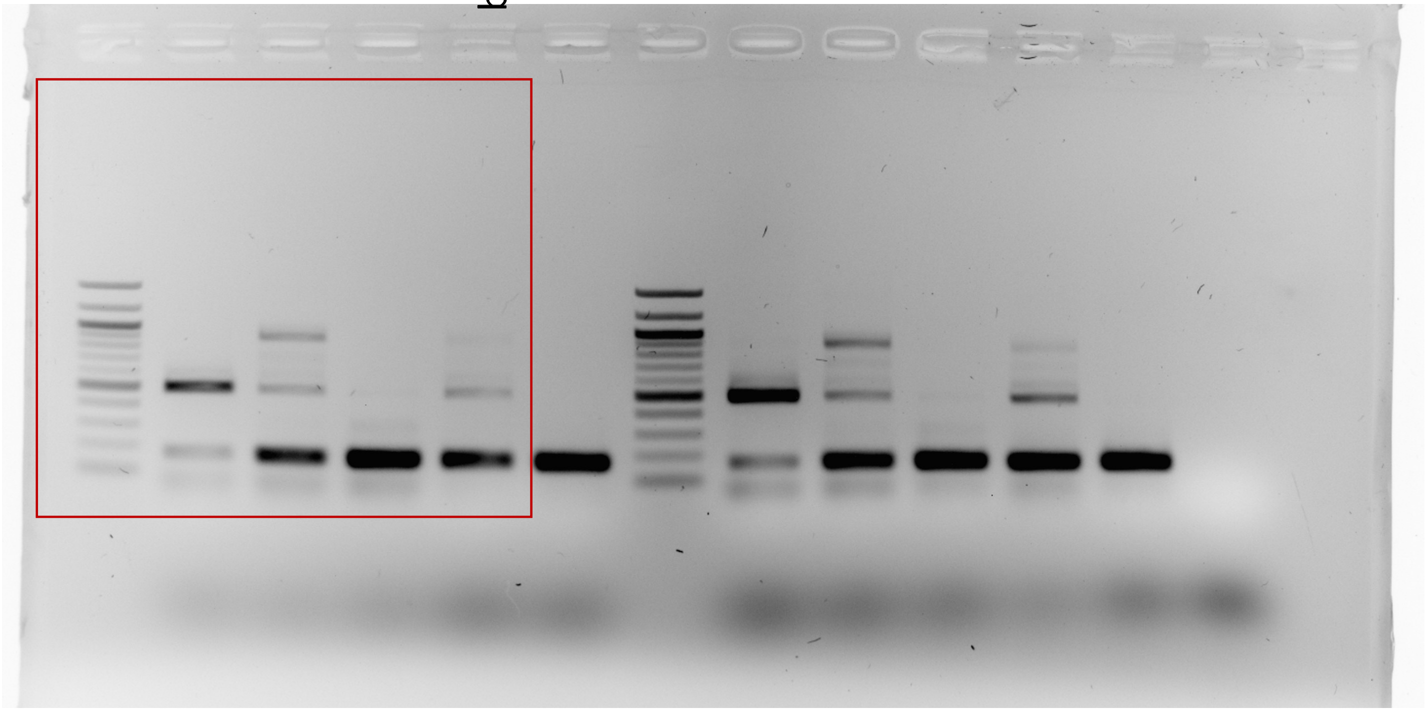

Ex27-28/29: 510 bp

Ex27-31: 178 bp (as control to all samples)

Raw gel image S2P Figure

sls

Ex 9_F: CGCGCAGTATGTGCAAAAT
Ex 11_R: AAACCGTTCCACGAAAAGTG

From Oas et al., 2014

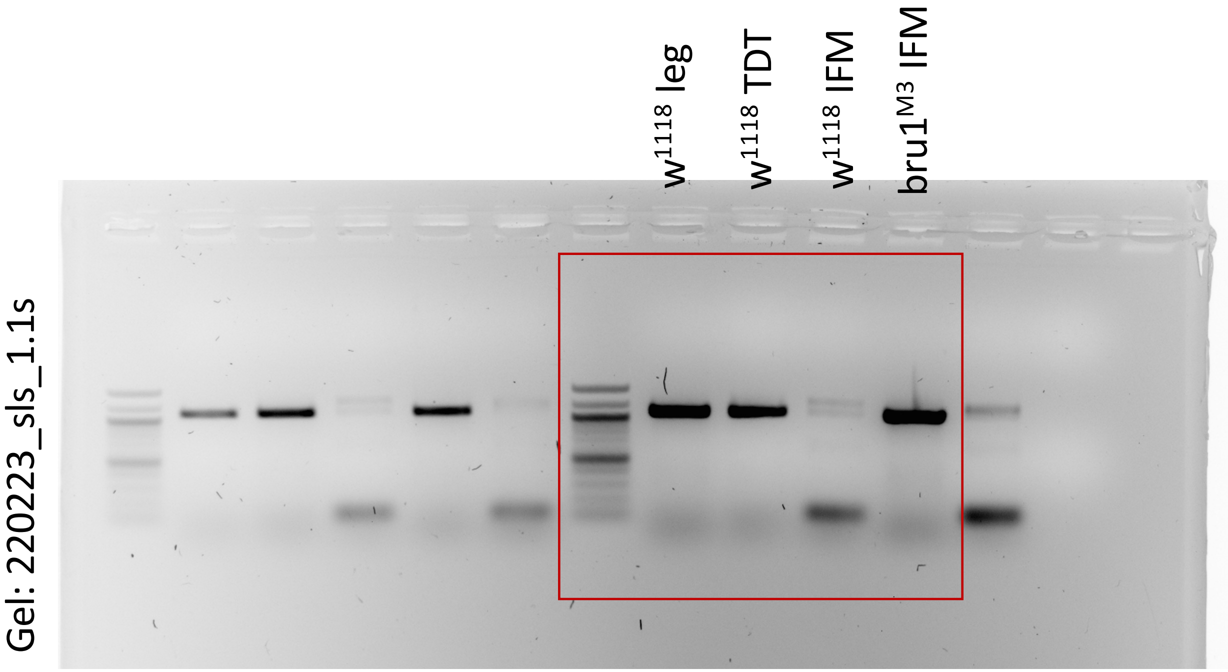

Tubular iso: 1075 bp
IFM iso: 106 bp

Raw gel image S2Q Figure
